# Randomized Controlled Trial of RTS,S/AS02_D_ and RTS,S/AS01_E_ Malaria Candidate Vaccines Given According to Different Schedules in Ghanaian Children

**DOI:** 10.1371/journal.pone.0007302

**Published:** 2009-10-02

**Authors:** Seth Owusu-Agyei, Daniel Ansong, Kwaku Asante, Sandra Kwarteng Owusu, Ruth Owusu, Naana Ayiwa Wireko Brobby, David Dosoo, Alex Osei Akoto, Kingsley Osei-Kwakye, Emmanuel Asafo Adjei, Kwadwo Owusu Boahen, Justice Sylverken, George Adjei, David Sambian, Stephen Apanga, Kingsley Kayan, Johan Vekemans, Opokua Ofori-Anyinam, Amanda Leach, Marc Lievens, Marie-Ange Demoitie, Marie-Claude Dubois, Joe Cohen, W. Ripley Ballou, Barbara Savarese, Daniel Chandramohan, John Owusu Gyapong, Paul Milligan, Sampson Antwi, Tsiri Agbenyega, Brian Greenwood, Jennifer Evans

**Affiliations:** 1 Kintampo Health Research Centre, Health Research Unit, Kintampo, Ghana; 2 London School of Hygiene and Tropical Medicine, London, United Kingdom; 3 School of Medical Sciences, Kwame Nkrumah University of Science and Technology, Kumasi, Ghana; 4 GlaxoSmithKline Biologicals, Rixensart, Belgium; 5 Bill and Melinda Gates Foundation, Seattle, Washington, United States of America; 6 PATH Malaria Vaccine Initiative, Bethesda, Maryland, United States of America; 7 Health Research Unit, Ghana Health Service, Ministry of Health, Accra, Ghana; 8 Kumasi Centre for Collaborative Research, Kumasi, Ghana; Queensland Institute of Medical Research, Australia

## Abstract

**Background:**

The target delivery channel of RTS,S candidate malaria vaccines in malaria-endemic countries in Africa is the World Health Organisation Expanded Program on Immunization. As an Adjuvant System, age de-escalation and schedule selection step, this study assessed 3 schedules of RTS,S/AS01_E_ and RTS,S/AS02_D_ in infants and young children 5–17 months of age in Ghana.

**Methodology:**

A Phase II, partially-blind randomized controlled study (blind to vaccine, not to schedule), of 19 months duration was conducted in two (2) centres in Ghana between August 2006 and May 2008. Subjects were allocated randomly (1∶1∶1∶1∶1∶1) to one of six study groups at each study site, each defining which vaccine should be given and by which schedule (0,1-, 0,1,2- or 0,1,7-months). For the 0,1,2-month schedule participants received RTS,S/AS01_E_ or rabies vaccine at one center and RTS,S/AS01_E_ or RTS,S/AS02_D_ at the other. For the other schedules at both study sites, they received RTS,S/AS01_E_ or RTS,S/AS02_D_. The primary outcome measure was the occurrence of serious adverse events until 10 months post dose 1.

**Results:**

The number of serious adverse events reported across groups was balanced. One child had a simple febrile convulsion, which evolved favourably without sequelae, considered to be related to RTS,S/AS01_E_ vaccination. Low grade reactions occurred slightly more frequently in recipients of RTS,S/AS than rabies vaccines; grade 3 reactions were infrequent. Less local reactogenicity occurred with RTS,S/AS01_E_ than RTS,S/AS02_D_. Both candidate vaccines were highly immunogenic for anti-circumsporozoite and anti-Hepatitis B Virus surface antigen antibodies. Recipients of RTS,S/AS01_E_ compared to RTS,S/AS02_D_ had higher peak anti-circumsporozoite antibody responses for all 3 schedules. Three dose schedules were more immunogenic than 2 dose schedules. Area under the curve analyses for anti-circumsporozoite antibodies were comparable between the 0,1,2- and 0,1,7-month RTS,S/AS01_E_ schedules.

**Conclusions:**

Both candidate malaria vaccines were well tolerated. Anti-circumsporozoite responses were greater with RTS,S/AS01_E_ than RTS,S/AS02_D_ and when 3 rather than 2 doses were given. This study supports the selection of RTS,S/AS01_E_ and a 3 dose schedule for further development in children and infants.

**Trial Registration:**

ClinicalTrials.gov NCT00360230

## Introduction

*Plasmodium falciparum* malaria is a major cause of human suffering and represents an important economic burden to sub-Saharan African countries [1], [2]. A safe and effective vaccine that prevents *P. falciparum* malaria would be an important addition to current control methods.

The RTS,S malaria vaccine candidate (GlaxoSmithKline, Rixensart, Belgium), is formulated with proprietary Adjuvant Systems which enhance the ability of the vaccine to induce a strong immune response. The AS02 Adjuvant System contains an oil-in-water emulsion with monophosphoryl lipid A (MPL) and QS21, a natural saponin molecule purified from the bark of the South American tree *Quillaja saponaria*. The AS01 Adjuvant System is based on liposomes and contains the same amounts of MPL and QS21 as AS02. Both preclinical studies and field studies in adults have suggested that the AS01 formulation is more immunogenic than the AS02 formulation [3]–[5].

AS01 and AS02 were initially developed as the adult formulations AS02_A_ and AS01_B_ (0.5 mL dose). For compatibility with standard auto-disable Expanded Program on Immunization (EPI) syringes, a 0.5 mL variant of the paediatric 0.25 mL dose of RTS,S/AS02_A_ (RTS,S/AS02_D_) and of RTS,S/AS01_B_ (RTS,S/AS01_E_) were developed.

A large study with the paediatric dose (0.25 mL) of the adult formulation RTS,S/AS02_A_, conducted in Mozambican children aged 1–4 years, demonstrated the vaccine to have an acceptable safety profile and to be efficacious against clinical malaria (vaccine efficacy of 35%) and severe malaria disease (vaccine efficacy of 49%) over a period of 18 months [6]. Subsequently, non-inferiority of the RTS,S/AS02_D_ formulation compared to a paediatric dose of RTS,S/AS02_A_ was demonstrated with respect to anti-circumsporozoite (CS) and anti-Hepatitis B Virus surface antigen (HBs) antibodies in children aged 3 to 5 years from Mozambique [7]. Both vaccines were shown to have a similar safety profile. More recently, a similar safety profile of RTS,S/AS02_D_ and RTS,S/AS01_E_ was demonstrated in children aged 18 months to 4 years in Gabon [8]; a trend towards better anti-CS and anti-HBs responses with RTS,S/AS01_E_ was observed. Following administration of RTS,S/AS02_D_ at 8, 12 and 16 weeks of age to infants in Tanzania, RTS,S/AS02_D_ had a promising safety profile, met pre-specified non-inferiority seroconversion rates for co-administered EPI antigens, and reduced the incidence of malaria infection (vaccine efficacy 65% over 6 months) [9]. In children aged 5 to 17 months in Kenya and Tanzania, proof-of-concept of the RTS,S/AS01_E_ candidate vaccine was recently demonstrated in a trial in which vaccine efficacy against malaria disease was 53%, over an average follow up period of 8 months [10].

The trial reported here is one of several age de-escalation, Adjuvant System and schedule selection steps undertaken prior to the conduct of a phase 3 RTS,S trial. The schedules under investigation were selected on the basis that they can be integrated into the existing EPI vaccination programme. Two or three vaccine doses one month apart (0,1- and 0,1,2-month schedule) could be administered together with two or three doses of DTP respectively, while a third delayed dose could be administered together with measles and yellow fever vaccines at 9 months of age (0,1,7-month schedule).

## Methods

The protocol for this trial [http://clinicaltrials.gov/: NCT00360230] and supporting CONSORT checklist are available as supporting information; see Checklist S1 and Protocol S1.

### Participants

The trial was conducted at two study centres in Ghana: a collaboration between the Kumasi Centre for Collaborative Research (KCCR), Kumasi and the School of Medical Sciences (SMS), Kwame Nkrumah University of Science and Technology (KNUST), Kumasi hosted at the Agogo Presbyterian Hospital and the Kintampo Health Research Centre (KHRC), Ghana Health Service, Ministry of Health, Kintampo.

The two centers are about 200 km away from each other. The recruitment area for the KCCR/SMS center was the Agogo town, while a more rural population was recruited at KHRC. Literacy rates are similar in both populations. The main activity is farming. The main population groups around KHRC comprise people from the Bono and Mo tribes and there is a large population from northern Ghana that have permanently migrated into Kintampo District. The majority of the population around KCCR/SMS is Akan with a small group of migrants from the north of the country. The study occurred at the same time in the two sites. Although data for health indicators such as HIV prevalence and malaria transmission were not collected as part of the study, the climate and basic health indicators are fairly similar with HIV rates under 4% in both areas. Malaria transmission intensity is intense, perennial (269 infectious bites/person/year and a prevalence of malaria parasitaemia among children less than 5 years of about 50% throughout the whole year in Kintampo in 2004) (Owusu-Agyei S., personal communication). Impregnated bednets were distributed at screening to potential study participants, regardless of whether children were then enrolled in the trial.

The protocol was approved by the Food and Drugs Board, Ghana; the Ghana Health Service Ethical Review Committee, Accra, Ghana; the KHRC Institutional Ethics Committee, the KHRC Scientific Review Committee; the Committee on Human Research Protection and Ethics, SMS, KNUST, Kumasi, Ghana; the London School of Hygiene and Tropical Medicine (LSHTM) ethics committee, London, UK and the Western Institutional Review Board, Washington, USA. The trial was undertaken according to the International Conference on Harmonization, Good Clinical Practice guidelines and was monitored by GlaxoSmithKline (GSK) Biologicals. The study was overseen by a formally constituted Data Safety Monitoring Board (DSMB) operating under a charter. The DSMB reviewed safety data from a RTS,S/AS trial in older children [8] prior to authorising the start of this study, and from a subset of children post dose 1 and post dose 2, prior to progression to the next vaccination dose within this trial. A Local Safety Monitor was designated at each site whose overall role was to support the clinical investigator and to act as a link between the investigator and the DSMB.

At the KCCR/SMS a register of potentially eligible subjects was made from children attending the immunisation clinics in the town of Agogo. KHRC has a Health and Demographic Surveillance System (KHDSS) in place that registers births, deaths and migrations to and from the area. The KHDSS was used to compile a list of potentially eligible children from an electronic database held at KHRC. In both study sites information was distributed to parents/legally accepted representatives of potential participants through meetings organized in the community, followed by several steps of family and individual based information sessions, with supporting information material. Reinforcement and checking of understanding was done with the support of pre-established documents addressing frequently asked questions. The whole process happened in the potential participant's own language, in the presence of an impartial literate member of the potential participant's community if parents/legally accepted representatives could not read or write.

To qualify for enrolment children had to be free of obvious health problems as established by medical history, clinical examination and laboratory blood markers for safety at screening.

### Procedures and interventions (Table 1)

Access of the study population to general clinical care according to national recommendations was facilitated, in collaboration with the government services. Hospital services were open for care on a continuous basis. Access to care was facilitated through the reimbursement of transport to the hospital and direct or phone access to field based study staff for the organisation of transport if needed. Malaria episodes were treated as recommended by the Ministry of Health of Ghana with oral artesunate and amodiaquine and IV or IM quinine if hospital admission was needed.

Both in Kintampo and Agogo, study vaccination sessions were organised in specially dedicated rooms with separated space for vaccine preparation and administration, located in the vicinity of standard EPI clinics, the hospital and research center. Recipients of candidate vaccine were administered lyophilised RTS,S reconstituted with 0.5 mL of either AS02_D_ or AS01_E_ Adjuvant Systems. Both candidate and control vaccines were administered intramuscularly into the deltoid muscle of the left arm on a 0,1-, 0,1,2- or 0,1,7-month schedule. In KHRC, children on the 0,1,2-month schedule received rabies vaccine as a control (Rabipur®; Chiron Behring GmbH). In the KCCR/SMS, children on the 0,1,2-month schedule, received the RTS,S/AS02_D_ experimental vaccine as an active comparator. Randomisation to each of the other study groups was balanced between the two study sites. Vaccinees were observed for 60 minutes following each vaccination.

Volunteers were followed daily for the solicited adverse events (AEs) of pain, swelling, fever (defined as an axillary temperature ≥37.5°C), drowsiness, loss of appetite and irritability/fussiness for a total of 7 days following each vaccination. Unsolicited non-serious AEs were collected for 30 days following each dose. Serious AEs (SAEs) were recorded throughout the study period. Blood draws for safety evaluation and measurement of humoral responses were taken at scheduled time points during the study (see Table 1).

**Table 1:** Outline of study design.

		Month -1	Month 0	Days 0+6	Month 1	Month 2	Month 3	Month 4	Month 5	Month 6	Month 7	Month 8	Month 9	Month 10	Month 19
**Schedule: 0,1-month**															
**Vaccination**			X		X										
**Serology**	CS	X				X					X			X	X
	HBs	X				X									X
**Safety BS**		X		X		X					X			X	X
**Schedule: 0,1,2-month**															
**Vaccination**			X		X	X									
**Serology**	CS	X					X				X			X	X
	HBs	X					X								X
**Safety BS**		X		X			X				X			X	X
**Schedule: 0,1,7-month**															
**Vaccination**			X		X						X				
**Serology**	CS	X									X	X		X	X
	HBs	X										X			X
**Safety BS**		**X**		**X**							**X**	**X**		**X**	**X**

Double blind (observer blind) phase: screening (Month -1, Month 0, Days 0 and 6) and primary study (Months 1–10).

Single blind phase: extended follow up: Month 19.

Schedule 0,1-month: RTS,S/AS01E (N = 90*), RTS,S/AS02D (N = 90*).

Schedule 0,1,2-month : RTS,S/AS01E (N = 90*), RTS,S/AS02D (N = 45**), Rabies (N = 45***).

Schedule 0,1,7-month: RTS,S/AS01E (N = 90*), RTS,S/AS02D (N = 90*).

FU = follow-up; BS = blood sample; *KHRC = 45, KCCR/SMS = 45; **KCCR/SMS only; ***KHRC only.

### Objectives

The study was a phase II, controlled, randomised, partially-blinded study of 19 months duration of the safety and immunogenicity of two candidate malaria vaccine formulations, RTS,S/AS02_D_ and RTS,S/AS01_E_, when given according to three different immunisation schedules in 5–17 month old children in Ghana.

### Endpoints: safety

The primary safety outcome measure was the occurrence of SAEs from the time of first vaccination until 10 months post Dose 1. A SAE was defined per protocol as any untoward medical occurrence that was fatal, life-threatening, required hospitalisation, led to disability or incapacity, or was judged by investigator's as being medically important enough to be reported as serious. In order to maximize data capture about seizures, all seizures occurring within 30 days of vaccination had to be reported as SAEs. Data on seizures occurring within 7 days post vaccination were collected in a standard way according to Brighton collaboration guidelines [11]. Secondary safety endpoints included the occurrence of unsolicited AEs after each vaccination over a 30 day follow-up period, solicited local (pain, swelling) and general (measured fever, irritability/fussiness, drowsiness, loss of appetite) reactions over a 7 day period (day of vaccination and 6 subsequent days) following each vaccination. Grade 3 general reactions were defined as: fever, an axillary temperature ≥39.0°C; irritability/fussiness, crying that could not be comforted/prevented normal activity; drowsiness that prevented normal activity; loss of appetite, not eating at all. Grade 3 solicited local injection site reactions were defined as: pain, cried when limb moved/spontaneously painful; swelling, exceeding 20 mm in diameter. Safety laboratory blood assessments for haematological (haemoglobin, white blood cells [WBC], platelets), renal (creatinine), and hepatic (alanine aminotransferase [ALT]) parameters were conducted at specific time points during the study (see Table 1). Tertiary endpoints included an assessment of SAEs up to 19 months post Dose 1.

For each AE/SAE, investigators had to assess whether (yes or no) there was a reasonable possibility that the AE may have been caused by the investigational product, using clinical judgment and taking into account the natural history of the underlying diseases, concomitant therapy, other risk factors, the temporal relationship of the event to the investigational product and available information on experimental or marketed products. All solicited injection site reactions were considered causally related to vaccination.

### Endpoints: immunogenicity

Anti-CS and anti-HBs antibodies were assessed prior to vaccination and on several occasions until 19 months post dose 1 (see Table 1). Antibody levels against the CS protein tandem repeat epitope were measured by a standard, validated ELISA using theR32LR antigen that contains the sequence [NVDP(NANP)_15_]_2_LR [7]. Antibody responses against the HBs were quantified by a GSK validated sandwich ELISA. Briefly, 96-well microplates were coated with native HBs and after washing and blocking steps, dilutions of serum samples, controls and standard were added to the plate. After washing, a recombinant horseradish peroxidase conjugated HBs was added as secondary reagent. After a final washing step and a colorimetric reaction with 3,3′,5,5′ tetramethylbenzidine stopped by addition of sulphuric acid, the plates were read in an ELISA reader. Titres were calculated using the reference standard curve with a 4 parameter logistic fitting algorithm and expressed in mIU/mL. The cut-off was set at 3.3 mIU/mL.

### Sample size

For any comparison between two study groups, the study had 80 percent power to detect an approximately 3 fold increase of events, for an event occurring at a frequency of ten percent in the comparator group, by Fisher exact test (alpha 5%).

For the secondary immunogenicity endpoints, anti-CS titres, a sample size of 75 evaluable subjects per group would have 90% power to demonstrate equivalence of the two vaccine regimens under comparison (i.e. *x* versus *y*, 95% CI of the GMT ratio *x*/*y* is within the range 0.33 to 3.0) at any time point assuming a log standard deviation of 0.9 in both groups, alpha = 0.025.

### Randomisation and blinding

Subjects were allocated sequentially to treatment numbers in the order that they presented for vaccination. Treatment numbers were assigned to vaccines with a randomisation list generated using a standard SAS® (Statistical Analysis System) programme. Subjects were allocated randomly (1∶1∶1∶1∶1∶1) to one of six study groups at each study site, each defining which vaccine should be given and by which schedule (0,1-, 0,1,2- or 0,1,7-months). For the 0,1,2-month schedule this meant RTS,S/AS01_E_ or rabies vaccine at KHRC and RTS,S/AS01_E_ or RTS,S/AS02_D_ at KCCR/SMS. For the other schedules at both study sites, this meant either RTS,S/AS01_E_ or RTS,S/AS02_D_.

During the primary phase of the study, i.e. up to month 10, the study was partially blinded, whereby investigators involved in endpoint evaluation and parents/guardians were blinded to the vaccine administered, but not to the schedule. The vaccine administration occurred in a separate room in the presence of a vaccination team that was not involved in any other part in the study, as described in detailed standard procedures. From months 10 to 19, during the extended follow-up period, the study was single-blind as parents/guardians remained blind to the study vaccine.

### Statistical methods

Analysis was carried out according to a DSMB approved report and analysis plan established before unblinding of trial data.

#### Safety

The proportion of subjects with a SAE, classified by the MedDRA preferred term level, reported from study start until study conclusion was tabulated with exact 95% confidence interval (CI). The percentage of subjects with at least one solicited local and general AE reported within 7 days post each vaccination was also tabulated, with exact 95% CI. The proportion of subjects who reported an unsolicited AE within 30 days post each vaccination, classified by the MedDRA preferred term level, was tabulated with exact 95% CI. Similar tables were generated for Grade 3 solicited and unsolicited AEs and for the relationship of the event to vaccination; per protocol, all solicited local AEs were considered to be related to vaccination. Biochemistry (ALT and creatinine) and haematology (haemoglobin, WBC, platelets) values outside of the reference ranges were described up to 19 months post Dose 1; frequency distribution of results by predefined toxicity grades were tabulated by group.

#### Immunogenicity

The percentage of subjects seropositive for anti-CS antibody (anti-CS antibody titres ≥0.5 EU/mL) with 95% CI was determined at each blood sampling time point (Table 1). Antibody titres were summarised by GMT with 95% CI. The area under the curve (AUC) of anti-CS responses over time was estimated over the vaccination period to month 7 (AUC7) and over the whole study duration to month 19 (AUC19), by trapezoidal rule using the consecutive blood samples. Month 2 data from the 0,1-month schedule was used to estimate the AUC for the 0,1,7-month schedule. Standardised AUC (sAUC), calculated by dividing AUC by follow-up time, was also calculated.

The seroprotective level for anti-HBs antibody was ≥10 mIU/mL. The percentage of subjects with seroprotective levels of anti-HBs with 95% CI was determined at each blood sampling time point (Table 1). Antibody titres were summarised by GMT with 95% CI at each blood sampling time point.

## Results

### Participant flow

**Figure 1:**
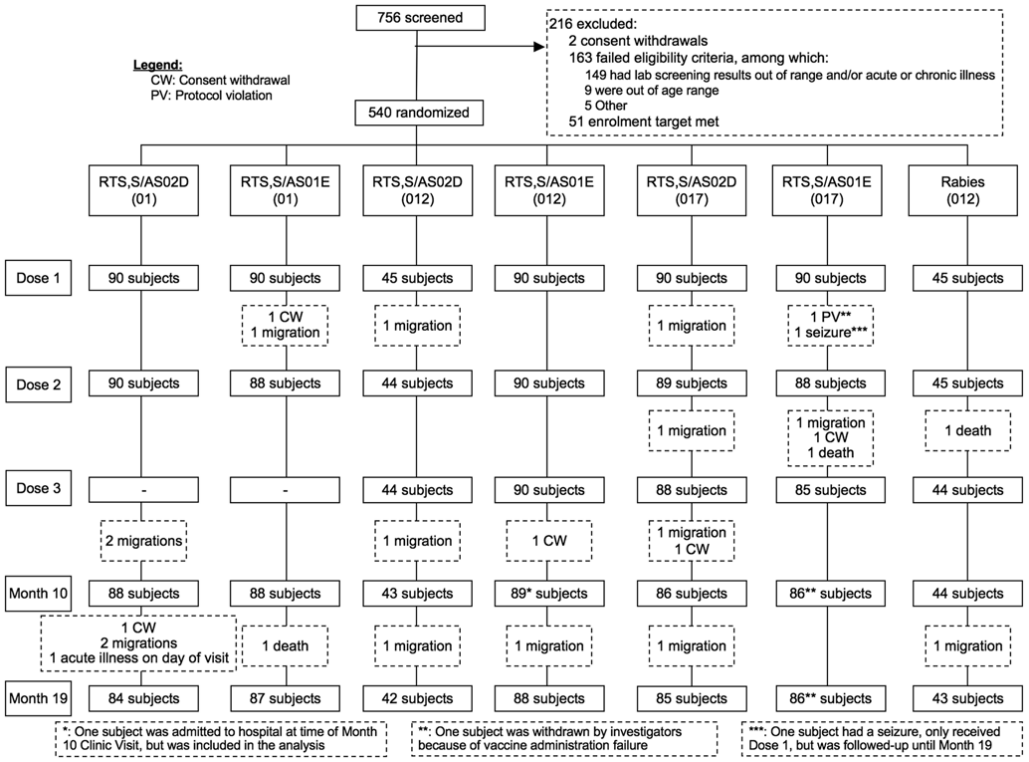
CONSORT diagram for study participants.

In total, 756 subjects were screened of whom 540 were enrolled into the study and evaluated for safety; 90 in each of the RTS,S/AS02_D_ (0,1- and 0,1,7-month) and RTS,S/AS01_E_ (0,1-, 0,1,2- and 0,1,7-month) groups and 45 in each of the RTS,S/AS02_D_ (0,1,2-month) and rabies vaccine groups (Figure 1). 531 subjects received all vaccine doses as scheduled and 521 were evaluable for the ATP cohort for immunogenicity. Twenty-five subjects (4.6%) were prematurely withdrawn from the study, predominantly due to migration from the study area (15 subjects).

The first subject was enrolled in the study on 30 August 2006 and the last study visit was made on 30 May 2008.

### Baseline data

For the total vaccinated cohort and the ATP cohort for immunogenicity the mean age at study entry was 10.7 months (standard deviation [SD] 3.5). Each group was balanced for gender and age, overall and by study center (Table 2).

**Table 2:** Summary of demographic characteristics by site and overall (Total vaccinated cohort).

	KHRC			KCCR			Total		
Group	Age (months) mean±SD	Gender F∶M (%)	Age for weight z-score mean±SD	Age (months) mean±SD	Gender F∶M (%)	Age for weight z-score mean±SD	Age (months) mean±SD	Gender F∶M (%)	Age for weight z-score mean±SD
RTS,S/AS02_D_ (0,1-month) N = 90	10.7±3.4	42∶58	−1.0±1.1	10.3±3.4	49∶51	−1.2±1.1	10.5±3.4	46∶54	−1.1±1.1
RTS,S/AS01_E_ (0,1-month) N = 90	10.7±3.1	38∶62	−1.0±1.0	10.1±3.8	56∶44	−1.1±1.1	10.4±3.4	47∶53	−1.1±1.0
RTS,S/AS02_D_ (0,1,2-month) N = 45	-	-	-	11.2±3.3	51∶49	−1.5±1.2	11.2±3.3	51∶49	−1.5±1.2
RTS,S/AS01_E_ (0,1,2-month) N = 90	10.8±3.6	42∶58	−1.0±1.1	11.0±3.9	47∶53	−1.3±1.1	10.9±3.7	44∶56	−1.1±1.1
RTS,S/AS02_D_ (0,1,7-month) N = 90	11.2±3.3	51∶49	−1.0±1.1	9.7±3.9	47∶53	−1.2±1.1	10.4±3.6	49∶51	−1.1±1.1
RTS,S/AS01_E_ (0,1,7-month) N = 90	11.4±3.2	62∶38	−0.7±1.0	10.0±3.3	44∶56	−1.1±1.0	10.7±3.3	53∶47	−0.9±1.0
Rabies (0,1,2-month) N = 45	10.9±3.5	60∶40	−0.8±1.2	-	-	-	10.9±3.5	60∶40	−0.8±1.2

KHRC = Kintampo Health Research Centre; KCCR = Kumasi Centre for Collaborative Research, Agogo.

### Safety

#### Serious adverse events

11.1% [5.5–19.5] and 20.0% [12.3–29.8] of the subjects in the RTS,S/AS02_D_ (0,1-month) group, 21.1% [13.2–31.0] and 25.6% [16.9–35.8] in the RTS,S/AS01_E_ (0,1-month) group, 11.1% [3.7–24.1] and 24.4% [12.9–39.5] in the RTS,S/AS02_D_ (0,1,2-month) group, 14.4% [7.9–23.4] and 24.4% [16.0–34.6] in the RTS,S/AS01_E_ (0,1,2-month) group, 18.9% [11.4–28.5] and 26.7% [17.9–37.0] in the RTS,S/AS02_D_ (0,1,7-month) group, 15.6% [8.8–24.7] and 18.9% [11.4–28.5] in the RTS,S/AS01_E_ (0,1,7-month) group, and 13.3% [6.1–26.8] and 15.6% [6.5–29.5] in the Rabies (0,1,2-month) group reported at least one SAE from day of first vaccination to Month 10 (primary endpoint) and Month 19, respectively. No apparent imbalance between study groups in the occurrence of SAEs was observed, which was also true for the entire study duration, to Month 19 (not shown).

Four subjects died during the trial; one child from the RTS,S/AS01_E_ 0,1,7-month schedule group died at home 7 days after having received the second experimental vaccine dose. A diagnosis of bronchopneumonia was made by verbal autopsy. One child from the rabies vaccine group died in hospital from severe malaria with severe anaemia and sepsis 7 months after the last vaccine dose. One child from the RTS,S/AS01_E_ 0,1-month schedule group died in hospital of cerebral malaria 17 months after the last vaccine dose. Another child died in hospital of severe malaria, severe anaemia and convulsions, 18 months after enrolment. He was due to receive RTS,S/AS01_E_ (0,1,7-month schedule) but the first vaccination attempt failed, there was spillage of the attributed vaccine dose, and he received only a negligible amount of vaccine. After this failed attempt, he was not further vaccinated. None of the fatal SAEs were considered to be related to vaccination.

One SAE was considered to be related to vaccination: an 18 month old boy experienced a simple febrile seizure 14 hours after dose 3 of RTS,S/AS01_E_ at home. Earlier on the same day, fever had been recorded as part of the routine reactogenicity monitoring. A few hours after the episode of convulsion he was seen at the district hospital, and found to be well. Physical examination and laboratory assessment did not show any evidence of infectious disease. He was treated with paracetamol only. He was kept for observation for 2 days and went home thereafter.

Two children in the study were diagnosed with intussusception. The first case was a 14 month old boy who presented on the day following the first RTS,S/AS01_E_ vaccination (0,1-month schedule group) with mucoid bloody diarrhoea, vomiting and fever, and was treated with oral antibiotics. Five days later he returned with a mass in his epigastrium. The second case was a 7 month old boy who presented fifteen days after first RTS,S/AS02_D_ vaccination (0,1,7-month schedule group) with a 1 day history of bloody mucoid stool, abdominal pain and a mass in the epigastrium. Both children were referred to the tertiary level university hospital where the diagnosis of intussusception was confirmed and treated surgically. Both children recovered fully. On the basis of the absence of obvious pathogenic explanation for these vaccine components being causative of intussusceptions, as well as the fact that they occurred at different time intervals after vaccination and following vaccination with different vaccine formulations, the investigators considered the intussusceptions not to be related to vaccination. The DSMB concurred with this assessment.

#### Non-serious adverse events and reactogenicity

For each RTS,S vaccine formulation, reactogenicity seen after the first two doses in the three vaccine schedule groups was similar. Therefore the description of reactogenicity focuses on the data from the 0,1,2- and 0,1,7-month schedules and data from the 0,1-month schedule groups is not presented here.

**Figure 2:**
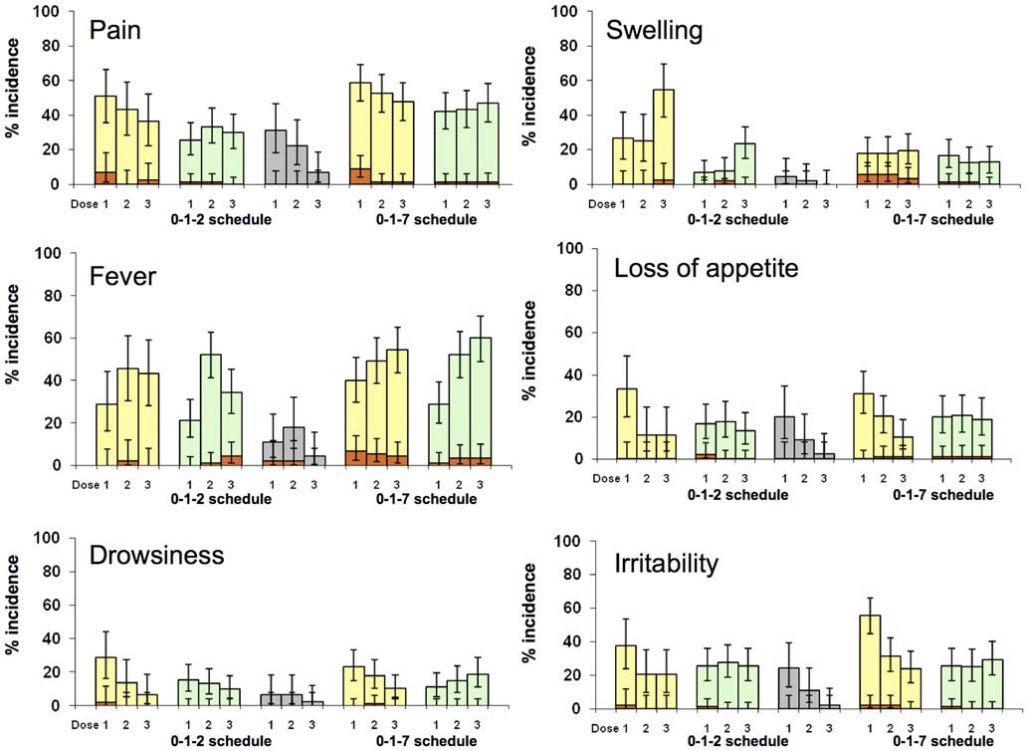
Percentage of solicited events post dose 1, 2 and 3 (Total Vaccinated Cohort). Yellow = RTS,S/AS02_D_; Green = RTS,S/AS01_E_; Grey = Rabies vaccine; Orange = Grade 3 Bars represent 95% confidence intervals.

Pain at the injection site was the most frequently reported solicited local symptom. It was reported with a higher frequency in the RTS,S/AS02_D_ compared to the RTS,S/AS01_E_ group (overall/dose: 0,1,2-month schedule, 43.6% vs 29.6% [rabies 20.1%]); 0,1,7-month schedule, 53.2% vs 44.1%, respectively). Although Grade 3 pain occurred with a low incidence, there was a trend towards a higher frequency of Grade 3 pain in the RTS,S/AS02_D_ compared to the RTS,S/AS01_E_ group (Figure 2).

A similar pattern was observed for swelling (overall/dose: 0,1,2-month, RTS,S/AS02_D_ 35.3% vs RTS,S/AS01_E_ 12.6% [rabies 2.2%]; 0,1,7-month, RTS,S/AS02_D_ 18.4% vs RTS,S/AS01_E_ 14.1%). Grade 3 swelling events were infrequent (Figure 2).

Fever was the most frequently reported solicited general symptom, occurring with a similar incidence in both the RTS,S/AS02_D_ and RTS,S/AS01_E_ vaccine groups and in both the 0,1,2-month (overall/dose: 39.1% vs 35.9%, respectively [rabies 11.2%]) and the 0,1,7-month (47.9% vs 46.8%, respectively) schedules (Figure 2). Grade 3 fever events were reported in the 0,1,2-month group following 0.8% and 1.9% of RTS,S/AS02_D_ and RTS,S/AS01_E_ doses respectively (rabies 1.5%), and in the 0,1,7-month group following 5.6% and 2.7% of RTS,S/AS02_D_ and RTS,S/AS01_E_ doses, respectively.

Unsolicited non-serious AEs were reported by a similar proportion of subjects who received RTS,S/AS02_D_ or RTS,S/AS01_E_ in the 0,1,2-month (53.4% vs 57.4% [rabies 59.7%]) and in the 0,1,7-month (93.3% vs 94.4%) groups. There was no trend in non-serious AEs Graded 3 for intensity across groups (0,1,2-month, 2.2% RTS,S/AS02_D_ vs 7.8% RTS,S/AS01_E_ [rabies 4.4%]; 0,1,7-month, 10.0% RTS,S/AS02_D_ vs 7.8% RTS,S/AS01_E_). There were no non-serious AEs estimated by the investigators to be related to vaccination.

Haematology (haemoglobin, WBC, platelets) and biochemistry (ALT, creatinine) values outside the normal range were infrequent. Three subjects had values graded 3. One subject in the RTS,S/AS02_D_ (0,1,7-month) group had elevated ALT at month 7 (416 U/mL). The child was found to be well, followed up clinically and the ALT level was normal at the next visit. One subject in the RTS,S/AS02_D_ (0,1,7-month) group had elevated ALT at month 7 (353 U/mL). He had a concomitant upper respiratory tract infection and was otherwise well. He was followed up clinically and the ALT level was normal at the next visit. One subject in the RTS,S/AS01_E_ (0,1,7-month) group had raised creatinine at month 8 (225 mg/dL). He was found to be well clinically, and at the next visit the creatinine concentration had reduced (67.1 mg/dL).

### Immunogenicity

All subjects were seropositive for anti-CS antibodies following 2 doses of either RTS,S/AS01_E_ or RTS,S/AS02_D_; low, background levels of anti-CS antibodies were found in rabies-vaccinated subjects.

In general, the levels of antibody responses in Kintampo were slightly higher than those in Agogo. For example, in the 0,1,7-month schedule groups, peak (month 8) anti-CS GMT following vaccination with RTS,S/AS02_D_ was 230 (95% CI 171, 309) in Agogo and 316 (95% CI 228, 437) in Kintampo, and following vaccination with RTS,S/AS01_E_ was 363 (95% CI 274, 481) in Agogo and 383 (95% CI 301, 488) in Kintampo (other data not shown). This trend was also observed for anti-hepatitis B antibodies (data not shown). The general conclusions from analysis of site-specific results (data not shown) were similar to those following review of pooled data from the two research centres as presented here.

Within each vaccination schedule group, the RTS,S/AS01_E_ formulation consistently yielded higher peak anti-CS responses as compared to RTS,S/AS02_D_. The highest anti-CS GMTs were seen with the 0,1,2-month schedule at month 3 (632 EU/mL [95% CI 554, 720]). With the same schedule, the RTS,S/AS02_D_ formulation induced anti-CS GMTs of 367 EU/mL (95% CI 293, 459). With the 0,1,7-month schedule at month 8, anti-CS GMTs were 373 EU/mL (95% CI 311, 447) for the RTS,S/AS01_E_ formulation and 272 EU/mL (95% CI 219, 339) for the RTS,S/AS02_D_ formulation. With the 0,1-month schedule at month 2, anti-CS GMTs were 483 EU/mL (95% CI 395, 591) for the RTS,S/AS01_E_ formulation and 318 EU/mL (95% CI 269, 377) for the RTS,S/AS02_D_ formulation.

At month 7, subjects having received two vaccine doses (i.e. the 0,1-month schedule) had lower anti-CS GMTs (AS02_D_: 35 EU/mL [95% CI: 26, 46]; AS01_E_: 53 EU/mL [95% CI: 41, 68]) than subjects having received 3 vaccine doses (0,1,2-month schedule) (AS02_D_: 78 EU/mL [95% CI: 58, 106]; AS01_E_: 162 EU/mL [95% CI: 134, 196]). This trend was also observed at month 19.

**Figure 3:**
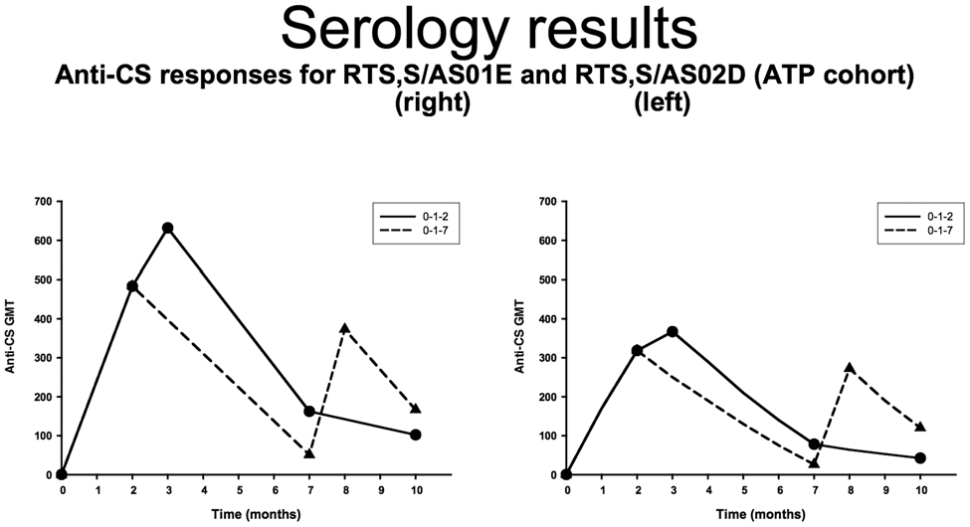
Anti-CS GMTs (EU/mL) responses over time (0,1,2- and 0,1,7-month schedules; ATP Cohort for Immunogenicity). Note : There was no month 2 blood sample in the 0,1,7-month schedule group. Month 2 data from the 0,1-month schedule group was used instead.

At month 19, similar anti-CS levels were found in both RTS,S/AS01_E_ schedule groups (0,1,7-month schedule 51 EU/mL [95% CI: 40, 66], 0,1,2-month schedule 46 EU/mL [95% CI: 37, 57]). In the RTS,S/AS02_D_ groups, the 0,1,7-month schedule induced higher month 19 anti-CS titres as compared to the 0,1,2-month schedule (44 EU/mL [95% CI: 33, 58] vs 20 EU/mL [95%CI: 14, 29] respectively) (Figure 3).

For all schedules, AUC was consistently higher for the RTS,S/AS01_E_ groups compared to the RTS,S/AS02_D_ groups (Table 3). For the RTS,S/AS01_E_ formulation, anti-CS sAUC19 over the whole study duration were comparable for the 0,1,2- and 0,1,7-month schedules and higher than for the 0,1-month schedule; the same trend, but less pronounced was observed for the RTS,S/AS02_D_ formulation. Anti-CS responses during the vaccination period (sAUC7) were high for all study groups with an apparent trend towards the highest values with the 0,1,2-month schedule in the RTS,S/AS01_E_ group.

**Table 3:** GMTs and sAUC for anti-CS antibodies and GMTs for anti-HBs antibodies (ATP Cohort for Immunogenicity).

Schedule	0,1-month		0,1,2-month			0,1,7-month	
	RTS,S/AS02_D_	RTS,S/AS01_E_	RTS,S/AS02_D_	RTS,S/AS01_E_	Rabies	RTS,S/AS02_D_	RTS,S/AS01_E_
**Anti-CS (EU/mL); GMTS [95% CI]**							
SCR	0.3 [0.3, 0.4]	0.3 [0.3, 0.4]	0.3 [0.2, 0.3]	0.3 [0.3, 0.4]	0.3 [0.3, 0.4]	0.3 [0.3, 0.3]	0.3 [0.3, 0.4]
M2	318 [269, 377]	483 [395, 591]					
M3	-	-	367 [293, 459]	632 [554, 720]	0.4 [0.3, 0.6]	-	-
M7	35 [26, 46]	53 [41, 68]	78 [58, 106]	162 [134, 196]	0.3 [0.3, 0.4]	26 [20, 34]	51 [40, 64]
M8	-	-	-	-	-	272 [219, 339]	373 [311, 447]
M10	20 [15, 27]	32 [23, 40]	43 [32, 60]	102 [83, 125]	0.3 [0.3, 0.4]	120 [92, 156]	167 [140, 198]
M19	10 [7], [14]	15 [11], [21]	20 [14, 29]	46 [37, 57]	0.5 [0.3, 0.7]	44 [33, 58]	51 [40, 66]
**Anti-CS sAUC; Gmean [95% CI]**							
sAUC7	181 [151, 218]	269 [218, 332]	225 [182, 279]	371 [324, 426]	-	166* [139, 198]	249* [204, 302]
sAUC19	83 [69, 101]	124 [101, 153]	113 [90, 143]	200 [173, 232]	-	141 [116, 173]	203 [172, 240]
**Anti-HBs (mIU/mL); GMTS [95% CI]**							
SCR	101 [68, 149]	108 [74, 158]	109 [61, 195]	82 [61, 111]	108 [64, 183]	88 [61, 128]	90 [61, 133]
M2	17043 [10467, 27751]	15107 [9508, 24001]					
M3			30000 [18799, 47874]	34935 [25178, 48474]	122 [63, 235]		
M8						96754 [72062, 129908]	103225 [83035, 128324]
M19	3510 [2398, 5137]	4478 [3155, 6357]	5112 [3350, 7803]	7106 [5161, 9784]	114 [58, 227]	17191 [12529, 23589]	13386 [9661, 18548]

SCR = screening visit; sAUC 7/19 = standardised area under the curve over the vaccination period to Month 7/Month 19.

* sAUC7 here represents a period during which only the first two vaccine doses were given, as for the 0,1-month schedule.

All the subjects in this study had previously received hepatitis B vaccination as part of their national EPI, with at least 84% of subjects having seroprotective levels of anti-HBs antibodies at screening. All children who received at least 2 RTS,S vaccine doses had seroprotective levels of anti-HBs antibodies, except one subject in the RTS,S/AS02_D_ 0,1-month schedule group. Seropositivity rates in the rabies group were 84%, illustrating a persistence of the response induced by the prior hepatitis B EPI vaccination.

Very high levels of anti-HBs GMTs were seen in subjects vaccinated with RTS,S vaccines; no significant difference was seen between RTS,S/AS01_E_ and RTS,S/AS02_D_ formulations within vaccine schedules (Table 3). One month post last dose and at month 19, the 0,1,7-month schedule generated the highest anti-HBs GMTs.

## Discussion

The development of a safe, effective malaria vaccine accessible to those who need it most is a critical global public health priority [12]. Evidence of immunogenicity, efficacy and a favourable safety profile of the RTS,S/AS02 candidate malaria vaccine has been demonstrated in adults [13]–[16] and subsequently in children and infants [7], [17]–[21].

Pre-clinical data suggested that the liposomal Adjuvant System formulation RTS,S/AS01 induces stronger immune responses as compared to RTS,S/AS02 [3]. In healthy human adults RTS,S/AS01, as compared to RTS,S/AS02, induced higher levels of anti-CS antibodies and CS-specific CD4 positive helper T cells expressing markers of activation and/or effector cytokines, and a trend towards higher protection against infection following experimental sporozoite challenge [4]. The good safety profile and high immunogenicity of the RTS,S/AS01 formulation was confirmed in malaria-exposed adults in Kenya [5].

Recently completed trials have compared the paediatric formulations of the candidate vaccines RTS,S/AS02_D_ and RTS,S/AS01_E_ in children aged 18 months to 4 years from Gabon [8], assessed RTS,S/AS02_D_ when coadministered with EPI antigens in infants from Tanzania [9] and established proof-of-concept of RTS,S/AS01_E_ in children aged 5 to 17 months from Kenya and Tanzania [10]. The trial reported here is the next in a series of age de-escalation steps towards the EPI age range, contributing to adjuvant and schedule selection. This study is the first comparative assessment of both candidate vaccines in young children aged 5 to 17 months and assessed three different vaccination schedules (0,1-, 0,1,2- and 0,1,7-months).

Overall, both the RTS,S/AS02_D_ and RTS,S/AS01_E_ formulations were shown to have a good safety profile and were well tolerated. One subject experienced an episode of simple febrile seizure considered to be related to vaccination following the third dose of RTS,S/AS01_E_ at month 7. In the RTS,S program to date 5315 doses of RTS,S/AS02 and 3149 doses of RTS,S/AS01 have been administered to 1864 and 1145 children under 6 years of age, respectively. Out of these one other case of simple febrile seizure related to vaccination has occurred [10]. Post vaccination febrile seizures are a well described complication of vaccine-related fever [11]. In the great majority of the cases, such as in these that have occurred in the RTS,S programme, it is benign, and resolves without sequelae. Two cases of intussusception (one in a RTS,S/AS02_D_ vaccinated child, one in a RTS,S/AS01_E_ vaccinated child) were reported in this study. Intussusception is a condition known to occur in this age group, including in Ghana, although precise incidence rates are unknown [22]. No other case of intussusception has been described in the RTS,S program to date.

The analysis of reactogenicity data indicated that although recipients of the malaria vaccine candidates reported more local and general symptoms than recipients of the rabies vaccine, grade 3 reactions were infrequent. A trend towards less local reactogenicity in recipients of the RTS,S/AS01_E_ vaccine formulation as compared to RTS,S/AS02_D_ recipients was found.

In a previous study in children aged 18 months to 4 years from Gabon, both candidate vaccines were shown to have a good safety profile and to be well tolerated [8]. In the study in Gabon, an increase in solicited local symptoms of pain and swelling at the injection site with subsequent doses of either vaccine was observed; this was not seen in this study. More recently, the RTS,S/AS02_D_ formulation coadministered to infants with other routinely delivered EPI immunisations, and RTS,S/AS01_E_ administered to children aged 5 to 17 months from Kenya and Tanzania, had favourable safety profiles [9], [10].

Both candidate vaccines were highly immunogenic for anti-CS antibodies. Following two doses of either RTS,S/AS01_E_ or RTS,S/AS02_D_, all subjects had seropositive levels of anti-CS antibodies. The peak anti-CS titres were higher than those observed in previous studies in children with RTS,S candidate vaccines [6], [7], [8], [20], [21]. This may be related to the fact that in this study all children had been previously immunised against hepatitis B. Indeed, it has previously been observed that prior HBV vaccination appears to promote the immune response to both HBs and CS antigen components of RTS,S/AS02 and RTS,S/AS01 [8]. Such high anti-CS and –HBs titres were also observed following administration of RTS,S/AS01_E_ to young children previously vaccinated with hepatitis B vaccine in another study [10].

The antibody response to the CS protein is believed to be an important component or marker of protective immunity although, to date, no protective threshold of CS antibody response has been determined. Future studies with the RTS,S candidate vaccines will investigate the priming effect of a neonatal dose of hepatitis B vaccine on the evolution of CS titres induced by the malaria vaccine.

Recipients of RTS,S/AS01_E_ consistently had higher peak anti-CS responses compared to recipients of RTS,S/AS02_D_, irrespective of vaccination schedule. Although responses in subjects who received just 2 doses (i.e. 0,1-month schedule) were high, the decline from peak GMTs was more important than in subjects on a 3 dose regimen. Anti-CS titres were higher at month 10 in children on the 0,1,7-month schedule as compared to the 0,1,2-month schedule, for both RTS,S vaccine groups. At month 19 this was still apparent in children vaccinated with RTS,S/AS02_D_ but in children vaccinated with RTS,S/AS01_E_ they were similar.

In an attempt to estimate antibody response over the whole timeframe of the study, while acknowledging the variation in the responses over time, AUCs standardised over time were evaluated. The evaluation over the whole study duration (AUC19) showed that mean responses with the RTS,S/AS01_E_ formulation were similar for both the 0,1,2- and 0,1,7-month schedules, which were higher than those seen with the two doses schedule. Although the anti-CS response following two doses was high, the peak, AUC7 and AUC19 in the 0,1,2-month schedules were higher. A two doses schedule is therefore probably not optimal.

Peak responses for anti-CS GMTs were observed in the 0,1,2-month schedules, which was an unexpected result as, in line with licensed Hepatitis B vaccine schedules, trials with a hepatitis B vaccine have shown that increasing the interval between the second and third dose enhances humoral response [23], [24]. Indeed, the highest peak responses for anti-HBs GMTs were observed in the 0,1,7-month schedules as compared to the 0,1,2-month schedule when considering the peak (one month post last dose) responses. It is not clear why the delayed third dose is associated with a marked increase in HBs response but not CS response and will need to be investigated further in delayed booster studies.

This study confirmed the previously demonstrated high immunogenicity of RTS,S based vaccines for anti-HBs antibodies, and that this is true also in subjects primed with hepatitis B vaccine.

In two similar Ghanaian populations, antibody responses were high though there was some variability between the two centers. Slightly higher antibody levels were found in KHRC participants, as compared to KCCR/SMS participants. Some variability magnitude of anti CS response between centers has previously been observed and the precise source of this heterogeneity is unclear. Future multicenter Phase 3 RTS,S studies will further characterize heterogeneity in vaccine immunogenicity and efficacy in different geographical locations.

This trial demonstrated that both formulations of the candidate malaria vaccines, RTS,S/AS01_E_ and RTS,S/AS02D, were well tolerated and highly immunogenic for anti-CS and anti-HBs antibodies in young children aged 5–17 months living in a malaria-endemic area. RTS,S based vaccines were well tolerated in children having previously received a full course of anti-HBs vaccine in infancy as part of EPI. The RTS,S/AS01_E_ formulation showed a trend towards less local reactogenicity and demonstrated higher immunogenicity for anti-CS antibodies. The 3 dose schedules were more immunogenic than 2 dose schedules with AUC analyses for anti-CS antibody levels and were comparable between the 0,1,2- and 0,1,7-month RTS,S/AS01_E_ schedules. In summary, this study supports further evaluation of the RTS,S/AS01_E_ candidate vaccine with both 0,1,2- and 0,1,7-month schedules in infants, and when co-administered with standard EPI antigens.

## Supporting Information

Checklist S1 CONSORT Checklist
(0.06 MB DOC)

Protocol S1 Trial Protocol
(1.04 MB PDF)
