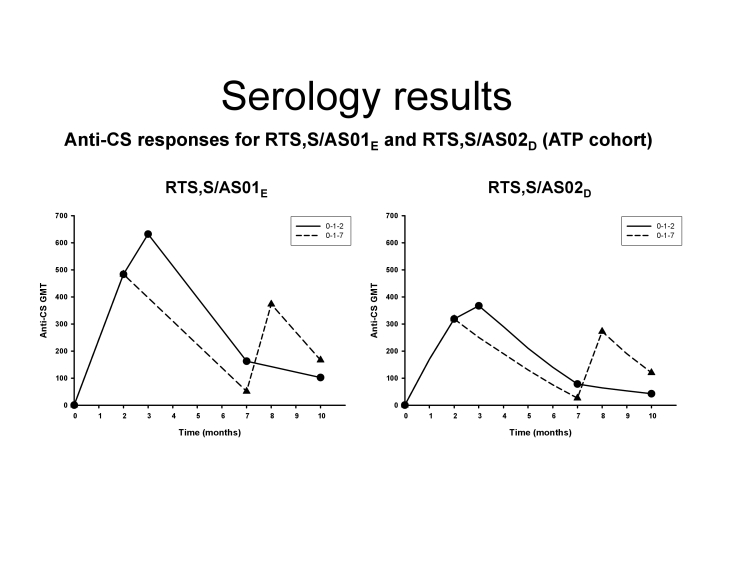# Correction: Randomized Controlled Trial of RTS,S/AS02_D_ and RTS,S/AS01_E_ Malaria Candidate Vaccines Given According to Different Schedules in Ghanaian Children

**DOI:** 10.1371/annotation/23dc5ebf-15ce-4f77-9aaf-9afec78e19b5

**Published:** 2010-11-17

**Authors:** Seth Owusu-Agyei, Daniel Ansong, Kwaku Asante, Sandra Kwarteng Owusu, Ruth Owusu, Naana Ayiwa Wireko Brobby, David Dosoo, Alex Osei Akoto, Kingsley Osei-Kwakye, Emmanuel Asafo Adjei, Kwadwo Owusu Boahen, Justice Sylverken, George Adjei, David Sambian, Stephen Apanga, Kingsley Kayan, Johan Vekemans, Opokua Ofori-Anyinam, Amanda Leach, Marc Lievens, Marie-Ange Demoitie, Marie-Claude Dubois, Joe Cohen, W. Ripley Ballou, Barbara Savarese, Daniel Chandramohan, John Owusu Gyapong, Paul Milligan, Sampson Antwi, Tsiri Agbenyega, Brian Greenwood, Jennifer Evans

Figure 3 was published with an error in the labeling. RTS,S/AS01E is actually shown on the left and RTS,S/AS02D is shown on the right. Please view the correct figure 3 here: 

**Figure:**