# Supplementary material for: Randomized Controlled Trial of RTS,S/AS02D and RTS,S/AS01E Malaria Candidate Vaccines Given According to Different Schedules in Ghanaian Children
Source: PLoS One. 2009 Oct 2;4(10):e7302. doi: 10.1371/journal.pone.0007302 (PMC2750750; doi:10.1371/journal.pone.0007302)
Supplement: Protocol S1 — Trial Protocol (1.04 MB PDF) [file pone.0007302.s002.pdf]

GlaxoSmithKline Biologicals
Rue de l'Institut 89, 1330 Rixensart,
Belgium.

**Study vaccines**	GlaxoSmithKline Biologicals' candidate *Plasmodium falciparum* malaria vaccine RTS,S/AS02D and GlaxoSmithKline Biologicals' candidate *Plasmodium falciparum* malaria vaccine RTS,S/AS01E and Chiron's Rabies vaccine, Rabipur®.
**eTrack study number**	106367
**eTrack abbreviated title**	Malaria-047
**Investigational New Drug (IND) number**	~~BB-IND to be determined~~ ***BB-IND 12937*** **Amended (04 July 2006)**
**Date of approval**	Final Version: 13 February 2006
Amendment 1	04 July 2006
**Title**	A Phase II randomized, controlled, partially-blind study of the safety and immunogenicity of GlaxoSmithKline Biologicals' candidate *Plasmodium falciparum* vaccines RTS,S/AS02D and RTS,S/AS01E, when administered IM according to one of three dose schedules in children aged 5 to 17 months living in Ghana.

GlaxoSmithKline Biologicals

**Coordinating author**	Conor Cahill, Scientific Writer
**Coordinating author Amendment 1**	Marie-Sylvie Remacle, Scientific Writer
**Contributing authors**	• Nathalie Annez, Central Study Coordinator<br>• W Ripley Ballou, Vice President – Clinical R&D, Early Development<br>• Ozzie Berger, US Regulatory Affairs<br>• Joe Cohen, Vice President – R&D, Vaccines For Emerging Diseases Program<br>• Sabine Corachan, Central Study Coordinator<br>• Marie-Ange Demoitié, Clinical Read-Outs Manager – R&D, Vaccines for Emerging Diseases Program<br>• Marie Claude Dubois, Project Manager – Disease Area Programme Vaccines for Emerging Diseases<br>• Amanda Leach, Clinical Development Manager<br>• Marc Lievens, Statistician<br>• Opokua Ofori-Anyinam, Clinical Development Manager<br>• Arlette Simonon, Central Study Coordinator<br>• Joelle Thonnard, Manager, Post Marketing Surveillance<br>• Marie Chantal Uwamwezi, Scientist – Regulatory Development Unit III<br>• Wendy Valinski, US Regulatory Affairs<br>• Johan Vekemans, Clinical Development Manager

Copyright 2006 the GlaxoSmithKline group of companies. All rights reserved. Unauthorized copying or use of this information is prohibited.

GSK Biologicals' Protocol DS V 12.2

**eTrack study number**	106367
**eTrack abbreviated title**	Malaria-047
**IND number**	~~BB-IND to be determined~~ ***BB-IND 12937***
	**Amended (04 July 2006)**
**Date of approval**	Final Version: 13 February 2006
Amendment 1	04 July 2006
**Title**	A Phase II randomized, controlled, partially-blind study of the safety and immunogenicity of GlaxoSmithKline Biologicals' candidate *Plasmodium falciparum* vaccines RTS,S/AS02D and RTS,S/AS01E, when administered IM according to one of three dose schedules in children aged 5 to 17 months living in Ghana.

Kumasi Centre for Collaborative Research (KCCR)/
School of Medical Sciences (SMS), Kumasi, Ghana

- Tsiri Agbenyega, School of Medical Sciences
- Daniel Ansong, School of Medical Sciences
- Jennifer Evans, Kumasi Centre for Collaborative Research

Kintampo Health Research Centre (KHRC), Kintampo, Ghana

- Seth Owusu Agyei, Kintampo Health Research Centre
- Kwaku Poku Asante, Kintampo Health Research Centre
- Daniel Chandramohan, London School of Hygiene and Tropical Medicine
- Brian Greenwood, London School of Hygiene and Tropical Medicine
- John Gyapong, Health Research Unit
- Ruth Owusu, Kintampo Health Research Centre

Malaria Vaccine Initiative at Program for Appropriate Technology in Health
Contributing Author

- Filip Dubovsky, Scientific Director
- Carolyn Petersen, Director, Clinical & Regulatory Affairs
- Marla Sillman, Technical Coordinator
- Tonya Villafana, Senior Program Officer

GlaxoSmithKline Biologicals will act as sponsor for this trial

**eTrack study number**	106367
**eTrack abbreviated title**	Malaria-047
**IND number**	~~BB-IND to be determined~~ ***BB-IND 12937***<br>**Amended (04 July 2006)**
**Date of approval**	Final Version: 13 February 2006
Amendment 1	04 July 2006
**Title**	A Phase II randomized, controlled, partially-blind study of the safety and immunogenicity of GlaxoSmithKline Biologicals' candidate *Plasmodium falciparum* vaccines RTS,S/AS02D and RTS,S/AS01E, when administered IM according to one of three dose schedules in children aged 5 to 17 months living in Ghana.

Sponsor signatory approval

~~**Sponsor signatory:**~~	~~W Ripley Ballou~~<br>~~Vice President – Clinical R&D, Early Development~~
**Signature:**	______________________________
**Date:**	______________________________

Sponsor Information

eTrack study number 106367

eTrack abbreviated title Malaria-047

IND number ~~BB-IND to be determined~~ ***BB-IND 12937***

Amended (04 July 2006)

1. Principal Investigators (KCCR/SMS)

Tsiri Agbenyega

School of Medical Sciences, KNUST,
Kumasi, Ghana.

Tel: +233.51.60.294
Fax: +233.51.60.302
Mobile: +233.244.442.889
email: tsiri@ghana.com

Jennifer Evans

Kumasi Centre for Collaborative Research,
KNUST,
Kumasi, Ghana.

Tel: +233.51.60351
Mobile: +233.244.630.531
Fax: +233.51.62017
email: evans@kccr.de

2. Principal Investigators (KHRC)

Seth Owusu Agyei

Kintampo Health Research Centre,
P.O. Box 200,
Kintampo, Ghana.

Tel: +233.61.2445/28871
Fax: +233.61.28872/28873
email: seth.owusu-agyei@ghana-khrc.org

alternate email:
seth.owusu-agyei@lshtm.ac.uk

Kwaku Poku Asante

Kintampo Health Research Centre,
P.O. Box 200,
Kintampo, Ghana.

Tel: +233.61.2445/28871
Fax: +233.61.28872/28873
email: kwakupoku.asante@ghana-khrc.org

3. Medical Monitor

Johan Vekemans,
Clinical Development Manager,
GlaxoSmithKline Biologicals,
Rue de l'Institut 89, 1330 Rixensart,
Belgium.

Tel: +32.2.656.56.59
Mobile: +32.473.29.21.67
Fax: +32.2.656.80.44
email: johan.vekemans@gskbio.com

eTrack study number 106367

eTrack abbreviated title Malaria-047

IND number ~~BB-IND to be determined~~ ***BB-IND 12937***

Amended (04 July 2006)

4. Central Study Coordinator (KCCR/SMS)

Arlette Simonon
Central Study Coordinator,
GlaxoSmithKline Biologicals,
Rue de l'Institut 89, 1330 Rixensart,
Belgium.
Tel: +32.2.656.55.07
Fax: +32.2.656.80.44
email:arlette.simonon@gskbio.com

5. Central Study Coordinator (KHRC)

Nathalie Annez
Central Study Coordinator,
GlaxoSmithKline Biologicals,
Rue de l'Institut 89, 1330 Rixensart,
Belgium.
Tel: +32.2.656.57.48
Fax: +32.2.656.80.44
email: nathalie.annez@gskbio.com

6. Study Contacts for Reporting of a Serious Adverse Event occurring at Agogo (KCCR/SMS)

All four of the contacts listed below must be informed of each SAE occurring at Agogo (KCCR/SMS)

Central Study Coordinator (KCCR/SMS)

Arlette Simonon
Central Study Coordinator,
GlaxoSmithKline Biologicals,
Rue de l'Institut 89, 1330 Rixensart,
Belgium.
Tel: +32.2.656.55.07
Fax: +32.2.656.80.44
email:arlette.simonon@gskbio.com

Local Safety Monitor (KCCR/SMS)

Gyikua Plange-Rhule,
Department of Child Health,
KATH/SMS,
Kumasi, Ghana.
Tel: +233.20.813.77.80
email: gyikua@hotmail.com

Malaria Vaccine Initiative

Carolyn Petersen,
Malaria Vaccine Initiative, PATH,
7500 Old Georgetown Road, 12th Floor,
Bethesda, MD 20814,
USA.
Tel: +1.240.395.27.16
or Tel: +1.240.395.27.00
Fax: +1.240.395.25.91
email: sae@malariavaccine.org

alternate email:
cpetersen@malariavaccine.org

Study Contact at GSK Biologicals for Reporting Serious Adverse Events Manager Clinical Safety Vaccines

GSK Biologicals Clinical Safety Physician,
GlaxoSmithKline Biologicals,
Rue de l'Institut 89, 1330 Rixensart,
Belgium.
Tel: +32.2.656.87.98
Fax: +32.2.656.80.09
Mobile phone for 7/7 day availability:
+32.477.40.47.13
email: rix.ct-safety-vac@gskbio.com

**eTrack study number**	106367
**eTrack abbreviated title**	Malaria-047
**IND number**	~~BB-IND to be determined~~ ***BB-IND 12937***

Amended (04 July 2006)

7. Study Contacts for Reporting of a Serious Adverse Event occurring at Kintampo (KHRC)

All four of the contacts listed below must be informed of each SAE occurring at Kintampo (KHRC)

Central Study Coordinator (KHRC)

Nathalie Annez
Central Study Coordinator,
GlaxoSmithKline Biologicals,
Rue de l'Institut 89, 1330 Rixensart,
Belgium.

Tel: +32.2.656.57.48
Fax: +32.2.656.80.44
email: nathalie.annez@gskbio.com

Local Safety Monitor (KHRC)

Sam Newton
Clinical Research Fellow
Kintampo Health Research Centre,
Kintampo, Ghana

Tel: +233.61.28869
sam.newton@ghana-khrc.org

Malaria Vaccine Initiative

Carolyn Petersen,
Malaria Vaccine Initiative, PATH,
7500 Old Georgetown Road, 12th Floor,
Bethesda, MD 20814,
USA.

Tel: +1.240.395.27.16
or Tel: +1.240.395.27.00
Fax: +1.240.395.25.91
email: sae@malariavaccine.org

alternate email:
cpetersen@malariavaccine.org

Study Contact at GSK Biologicals for Reporting Serious Adverse Events Manager Clinical Safety Vaccines

GSK Biologicals Clinical Safety Physician,
GlaxoSmithKline Biologicals,
Rue de l'Institut 89, 1330 Rixensart,
Belgium.

Tel: +32.2.656.87.98
Fax: +32.2.656.80.09
Mobile phone for 7/7 day availability:
+32.477.40.47.13
email: rix.ct-safety-vac@gskbio.com

**eTrack study number**	106367
**eTrack abbreviated title**	Malaria-047
**IND number**	~~BB-IND to be determined~~ ***BB-IND 12937***
	**Amended (04 July 2006)**

8. Study Contact for Emergency Code Break

GSK Biologicals Clinical Safety Physician,
GlaxoSmithKline Biologicals,
Rue de l'Institut 89, 1330 Rixensart,
Belgium.

Tel: +32.2.656.87.98
Fax: +32.2.656.80.09
Mobile phone for 7/7 day availability: +32.477.40.47.13

9. Study Centers

1: Kumasi Centre for Collaborative Research (KCCR/SMS), Kumasi, Ghana

2: Kintampo Health Research Centre (KHRC), PO Box 200, Kintampo, Ghana

Investigator Agreements

**eTrack study number**	106367
**eTrack abbreviated title**	Malaria-047
**IND number**	~~BB-IND to be determined~~ ***BB-IND 12937***
	**Amended (04 July 2006)**
**Title**	A Phase II randomized, controlled, partially-blind study of the safety and immunogenicity of GlaxoSmithKline Biologicals' candidate *Plasmodium falciparum* vaccines RTS,S/AS02D and RTS,S/AS01E, when administered IM according to one of three dose schedules in children aged 5 to 17 months living in Ghana.

I agree:

- To assume responsibility for the proper conduct of the study at this site.
- To conduct the study in compliance with this protocol, any mutually agreed future protocol amendments, and with any other study conduct procedures provided by GlaxoSmithKline Biologicals (GSK Biologicals).
- ***To ensure that all persons assisting me with the study are adequately informed about the GSK Biologicals investigational product(s) and other study-related duties and functions as described in the protocol.***

Amended (04 July 2006)

- Not to implement any changes to the protocol without agreement from the sponsor and prior review and written approval from the Institutional Review Board (IRB) or Independent Ethics Committee (IEC), except where necessary to eliminate an immediate hazard to the subjects, or for administrative aspects of the study (where permitted by all applicable regulatory requirements).
- That I am thoroughly familiar with the appropriate use of the vaccines, as described in this protocol, and any other information provided by the sponsor, including, but not limited to, the following: the current Investigator's Brochure (IB) or equivalent document, IB supplement (if applicable), prescribing information (in the case of a marketed vaccine).
- That I am aware of, and will comply with, "Good Clinical Practices" (GCP) and all applicable regulatory requirements.
- That I have been informed that certain regulatory authorities require the sponsor to obtain and supply, as necessary, details about the investigator's ownership interest in the sponsor or the investigational product, and more generally about his/her financial ties with the sponsor. GSK Biologicals will use and disclose the information solely for the purpose of complying with regulatory requirements.

Hence I:

- Agree to supply GSK Biologicals with any necessary information regarding ownership interest and financial ties (including those of my spouse and dependent children).
- Agree to promptly update this information if any relevant changes occur during the course of the study and for 1 year following completion of the study.
- Agree that GSK Biologicals may disclose any information it has about such ownership interests and financial ties to regulatory authorities.
- Agree to provide GSK Biologicals with an updated Curriculum Vitae and other FDA required documents.

Tsiri Agbenyega	Principal Coinvestigator, Kumasi Centre for Collaborative Research/School of Medical Sciences, KNUST

Investigator signature	Date

**eTrack study number**	106367
**eTrack abbreviated title**	Malaria-047
**IND number**	~~BB-IND to be determined~~ ***BB-IND 12937***
	**Amended (04 July 2006)**
**Title**	A Phase II randomized, controlled, partially-blind study of the safety and immunogenicity of GlaxoSmithKline Biologicals' candidate *Plasmodium falciparum* vaccines RTS,S/AS02D and RTS,S/AS01E, when administered IM according to one of three dose schedules in children aged 5 to 17 months living in Ghana.

I agree:

- To assume responsibility for the proper conduct of the study at this site.
- To conduct the study in compliance with this protocol, any mutually agreed future protocol amendments, and with any other study conduct procedures provided by GlaxoSmithKline Biologicals (GSK Biologicals).
- ***To ensure that all persons assisting me with the study are adequately informed about the GSK Biologicals investigational product(s) and other study-related duties and functions as described in the protocol.***

Amended (04 July 2006)

- Not to implement any changes to the protocol without agreement from the sponsor and prior review and written approval from the Institutional Review Board (IRB) or Independent Ethics Committee (IEC), except where necessary to eliminate an immediate hazard to the subjects, or for administrative aspects of the study (where permitted by all applicable regulatory requirements).
- That I am thoroughly familiar with the appropriate use of the vaccines, as described in this protocol, and any other information provided by the sponsor, including, but not limited to, the following: the current Investigator's Brochure (IB) or equivalent document, IB supplement (if applicable), prescribing information (in the case of a marketed vaccine).
- That I am aware of, and will comply with, "Good Clinical Practices" (GCP) and all applicable regulatory requirements.
- That I have been informed that certain regulatory authorities require the sponsor to obtain and supply, as necessary, details about the investigator's ownership interest in the sponsor or the investigational product, and more generally about his/her financial ties with the sponsor. GSK Biologicals will use and disclose the information solely for the purpose of complying with regulatory requirements.

Hence I:

- Agree to supply GSK Biologicals with any necessary information regarding ownership interest and financial ties (including those of my spouse and dependent children).
- Agree to promptly update this information if any relevant changes occur during the course of the study and for 1 year following completion of the study.
- Agree that GSK Biologicals may disclose any information it has about such ownership interests and financial ties to regulatory authorities.
- Agree to provide GSK Biologicals with an updated Curriculum Vitae and other FDA required documents.

Jennifer Evans	Principal Coinvestigator, Kumasi Centre for Collaborative Research/School of Medical Sciences, KNUST

Investigator signature ____________________ Date ____________________

**eTrack study number**	106367
**eTrack abbreviated title**	Malaria-047
**IND number**	~~BB-IND to be determined~~ ***BB-IND 12937***
	**Amended (04 July 2006)**
**Title**	A Phase II randomized, controlled, partially-blind study of the safety and immunogenicity of GlaxoSmithKline Biologicals' candidate *Plasmodium falciparum* vaccines RTS,S/AS02D and RTS,S/AS01E, when administered IM according to one of three dose schedules in children aged 5 to 17 months living in Ghana.

I agree:

- To assume responsibility for the proper conduct of the study at this site.
- To conduct the study in compliance with this protocol, any mutually agreed future protocol amendments, and with any other study conduct procedures provided by GlaxoSmithKline Biologicals (GSK Biologicals).
- ***To ensure that all persons assisting me with the study are adequately informed about the GSK Biologicals investigational product(s) and other study-related duties and functions as described in the protocol.***

Amended (04 July 2006)

- Not to implement any changes to the protocol without agreement from the sponsor and prior review and written approval from the Institutional Review Board (IRB) or Independent Ethics Committee (IEC), except where necessary to eliminate an immediate hazard to the subjects, or for administrative aspects of the study (where permitted by all applicable regulatory requirements).
- That I am thoroughly familiar with the appropriate use of the vaccines, as described in this protocol, and any other information provided by the sponsor, including, but not limited to, the following: the current Investigator's Brochure (IB) or equivalent document, IB supplement (if applicable), prescribing information (in the case of a marketed vaccine).
- That I am aware of, and will comply with, "Good Clinical Practices" (GCP) and all applicable regulatory requirements.
- That I have been informed that certain regulatory authorities require the sponsor to obtain and supply, as necessary, details about the investigator's ownership interest in the sponsor or the investigational product, and more generally about his/her financial ties with the sponsor. GSK Biologicals will use and disclose the information solely for the purpose of complying with regulatory requirements.

Hence I:

- Agree to supply GSK Biologicals with any necessary information regarding ownership interest and financial ties (including those of my spouse and dependent children).
- Agree to promptly update this information if any relevant changes occur during the course of the study and for 1 year following completion of the study.
- Agree that GSK Biologicals may disclose any information it has about such ownership interests and financial ties to regulatory authorities.
- Agree to provide GSK Biologicals with an updated Curriculum Vitae and other FDA required documents.

Seth Owusu Agyei   Principal Coinvestigator, Kintampo Health Research Centre

____   ________________________

Investigator signature   Date

**eTrack study number**	106367
**eTrack abbreviated title**	Malaria-047
**IND number**	~~BB-IND to be determined~~ ***BB-IND 12937***
	**Amended (04 July 2006)**
**Title**	A Phase II randomized, controlled, partially-blind study of the safety and immunogenicity of GlaxoSmithKline Biologicals' candidate *Plasmodium falciparum* vaccines RTS,S/AS02D and RTS,S/AS01E, when administered IM according to one of three dose schedules in children aged 5 to 17 months living in Ghana.

I agree:

- To assume responsibility for the proper conduct of the study at this site.
- To conduct the study in compliance with this protocol, any mutually agreed future protocol amendments, and with any other study conduct procedures provided by GlaxoSmithKline Biologicals (GSK Biologicals).
- ***To ensure that all persons assisting me with the study are adequately informed about the GSK Biologicals investigational product(s) and other study-related duties and functions as described in the protocol.***

Amended (04 July 2006)

- Not to implement any changes to the protocol without agreement from the sponsor and prior review and written approval from the Institutional Review Board (IRB) or Independent Ethics Committee (IEC), except where necessary to eliminate an immediate hazard to the subjects, or for administrative aspects of the study (where permitted by all applicable regulatory requirements).
- That I am thoroughly familiar with the appropriate use of the vaccines, as described in this protocol, and any other information provided by the sponsor, including, but not limited to, the following: the current Investigator's Brochure (IB) or equivalent document, IB supplement (if applicable), prescribing information (in the case of a marketed vaccine).
- That I am aware of, and will comply with, "Good Clinical Practices" (GCP) and all applicable regulatory requirements.
- That I have been informed that certain regulatory authorities require the sponsor to obtain and supply, as necessary, details about the investigator's ownership interest in the sponsor or the investigational product, and more generally about his/her financial ties with the sponsor. GSK Biologicals will use and disclose the information solely for the purpose of complying with regulatory requirements.

Hence I:

- Agree to supply GSK Biologicals with any necessary information regarding ownership interest and financial ties (including those of my spouse and dependent children).
- Agree to promptly update this information if any relevant changes occur during the course of the study and for 1 year following completion of the study.
- Agree that GSK Biologicals may disclose any information it has about such ownership interests and financial ties to regulatory authorities.
- Agree to provide GSK Biologicals with an updated Curriculum Vitae and other FDA required documents.

Kwaku Poku Asante   Principal Coinvestigator, Kintampo Health Research Centre

______________________________	____________________
Investigator signature	Date

Synopsis

Title A Phase II randomized, controlled, partially-blind study of the safety and immunogenicity of GlaxoSmithKline Biologicals' candidate *Plasmodium falciparum* vaccines RTS,S/AS02D and RTS,S/AS01E, when administered IM according to one of three dose schedules in children aged 5 to 17 months living in Ghana.

Indication/Study population Immunization of healthy male and female children aged 5 to 17 months at enrolment, if eligible according to inclusion and exclusion criteria.

Rationale The RTS,S/AS01E candidate combined malaria and hepatitis B vaccine is being developed for the routine immunization of infants and children living in malaria-endemic areas as part of the Expanded Program on Immunization (EPI). The RTS,S/AS01E vaccine consists of sequences of the circumsporozoite (CS) protein and hepatitis B surface antigen (HBsAg) with the proprietary adjuvant AS01E (proprietary liposomes, MPL® and Stimulon® QS21 immunostimulants).

Previous studies of GSK malaria vaccines in children have been conducted with the same antigen, but administered with an adjuvant formulation from the AS02 adjuvant family which consists of an oil-in-water emulsion, MPL® and QS21. The following table details the various vaccine formulations that have been trialled in humans (or are planned for human trials).

	Freeze-dried fraction	Liquid fraction				
Formulation	RTS,S (µg)		MPL® (µg)	QS21 (µg)	Dose volume	
RTS,S/AS02A (0.5 mL dose)	50	Oil-in-water emulsion	50	50	0.5 mL	Efficacy against infection, Gambian adults (Malaria-005)
RTS,S/AS02A (0.25 mL dose)	25	Oil-in-water emulsion	25	25	0.25 mL	Efficacy against clinical disease, Mozambican children (Malaria-026)
RTS,S/AS02D	25	Oil-in-water emulsion	25	25	0.5 mL	Pediatric formulation (Malaria-034, -038, -040)
RTS,S/AS01B	50	Liposomes	50	50	0.5 mL	Efficacy in challenge model, adults (Malaria-027)
RTS,S/AS01E	25	Liposomes	25	25	0.5 mL	Proposed pediatric formulation

The RTS,S/AS02A vaccine (0.25 mL dose) has demonstrated efficacy against infection and a range of illnesses caused by *P. falciparum* in children aged 1 to 4 years in Mozambique (Malaria-026). Up to 18 months post Dose 3 the Vaccine Efficacy (VE) determined as the time to the first clinical episode of malaria was 33%. VE against multiple attacks of malaria was 32% and against severe malaria was 49% over the 18 months of the trial.

RTS,S/AS02D, the EPI-compatible pediatric formulation of RTS,S/AS02A, is currently being assessed in infants in a malaria-endemic area. The Malaria-038 trial, taking place in Mozambique is being conducted to assess RTS,S/AS02D when administered in a staggered fashion with DTPw/Hib vaccine. The Malaria-040 trial, due to start in ~~Q1/Q2~~ ***Q3*** 2006 in Tanzania will assess RTS,S/AS02D when coadministered with DTPw/Hib vaccine. **Amended (04 July 2006)**

The RTS,S/AS01 vaccines have been developed in parallel with the RTS,S/AS02 vaccines with the aim of improving the immune response and increasing vaccine efficacy. A recent challenge study with RTS,S/AS01B in malaria-naïve adults (Malaria-027) has shown encouraging results, indicating a similar safety profile to that of RTS,S/AS02A, higher humoral immunogenicity, a favorable Th1 cell-mediated immune profile and a trend towards higher vaccine efficacy.

The first study in which RTS,S/AS01 vaccines ~~will be~~ ***is*** assessed in children is Malaria-046. That study ~~will take~~ ***takes*** place in children in Gabon in children between the ages of 18 months and 4 years in order to investigate safety, reactogenicity and immunogenicity of RTS,S/AS01E. **Amended (04 July 2006)**

RTS,S/AS01E (0.5 mL dose) is the proposed pediatric formulation which is composed of the same active constituents in the same quantities as a 0.25 mL dose of RTS,S/AS01B.

This study (Malaria-047), will compare three potential schedules (see table below) to optimize the immune response and determine vaccination schedule prior to the commencement of Phase III studies. A two-dose 0, 1-month regimen could offer the potential of rapid immunization, and for implementation would have logistical and cost-effectiveness advantages. The data suggesting that a two-dose schedule may be efficacious comes from early mosquito-challenge studies in malaria-naïve adults with the RTS,S/AS02A vaccine at the Walter Reed Army Institute of Research (WRAIR): estimates of efficacy for pooled data for two- and three-dose schedules were 37.8% (95% CI 17.3% to 53.3%) and 43.2% (95% CI 26.5% to 56.1%) respectively. The 0, 1, 2-month schedule has been used in a number of studies of RTS,S/AS02 vaccines in the past. The 0, 1, 7-schedule represents an alternative 3-dose schedule that might allow the third dose of candidate vaccines to be delivered during another existing EPI vaccination visit (e.g. measles vaccination at 9 months of age). The delay between administering Dose 2 and Dose 3 of vaccine may allow maturing of the immune system allowing for better stimulation of primed B-cells and T-cells.

One group of children on the 0, 1, 2-schedule will receive a Rabies vaccine as a control. One group on the same schedule will receive the RTS,S/AS02D experimental vaccine as an active comparator. **Amended**

(04 July 2006)

~~RTS,S/AS02D will not be assessed on a 0, 1, 2-schedule in this study as it has already been assessed in children during the trials Malaria-025, -026, -034 and is being assessed in Malaria-038 and -040.~~

Amended (04 July 2006)

Vaccine	Schedule (Months)	Number of children to be enrolled	Estimated number evaluable
RTS,S/AS01E (0.5 mL dose)	0, 1, 2	90	75
Rabies vaccine (0.5 mL dose)[a]	0, 1, 2	~~90~~ ***45***	~~75~~ ***40***
RTS,S/AS02D (0.5 mL dose)[b]	***0, 1, 2***	***45***	***40***
RTS,S/AS02D (0.5 mL dose)	0, 1, 7	90	75
RTS,S/AS01E (0.5 mL dose)	0, 1, 7	90	75
RTS,S/AS02D (0.5 mL dose)	0, 1	90	75
RTS,S/AS01E (0.5 mL dose)	0, 1	90	75
[a] The Rabies vaccine will only be used at Kintampo – KHRC ***[b] Enrollment will occur at Kumasi – KCCR/SMS***			

Amended (04 July 2006)

The dose intervals of all three schedules in this trial allow for potential incorporation into the EPI vaccination schedule, which has visits at approximately 2, 3, 4 and 9 months of age in the first year of life.

This product development plan is conducted under a partnership agreement with the Malaria Vaccine Initiative at PATH (MVI) and is guided by a joint MVI/GSK Steering Committee.

All current studies of the RTS,S candidate antigen conducted in children are overseen by a formally constituted DSMB operating under a charter.

Objectives

Primary

- For RTS,S/AS01E (on a 0, 1-month and a 0, 1, 2-month and a 0, 1, 7-month schedule) and RTS,S/AS02D (on a 0, 1-month, ***0, 1, 2-month*** and a 0, 1, 7-month schedule) when administered intramuscularly to children aged 5 to 17 months living in a malaria-endemic area:

Amended (04 July 2006)

  – to assess safety until ten months post Dose 1.

Secondary

- For RTS,S/AS01E (on a 0, 1-month and a 0, 1, 2-month and a 0, 1, 7-month schedule) and RTS,S/AS02D (on a 0, 1-month, ***0, 1, 2-***

month and a 0, 1, 7-month schedule) when administered intramuscularly to children aged 5 to 17 months living in a malaria-endemic area:

Amended (04 July 2006)

- to assess safety and reactogenicity until 30 days post Dose 1
- to describe the evolution of antibody responses to the circumsporozoite (CS) antigen until ten months post Dose 1
- to assess the antibody responses to the hepatitis B surface (HBs) antigen at one month post final vaccine dose.

Tertiary

- For RTS,S/AS01E (on a 0, 1-month and a 0, 1, 2-month and a 0, 1, 7-month schedule) and RTS,S/AS02D (on a 0, 1-month, ***0, 1, 2-month*** and a 0, 1, 7-month schedule) when administered intramuscularly to children aged 5 to 17 months living in a malaria-endemic area:

Amended (04 July 2006)

- to assess safety between ten months post Dose 1 up to 19 months post Dose 1
- to assess antibody responses to the circumsporozoite (CS) antigen at 19 months post Dose 1
- to assess antibody responses to the hepatitis B surface (HBs) antigen at 19 months post Dose 1.

Exploratory

- For RTS,S/AS01E (on a 0, 1-month and a 0, 1, 2-month and a 0, 1, 7-month schedule) and RTS,S/AS02D (on a 0, 1-month, ***0, 1, 2-month*** and a 0, 1, 7-month schedule) when administered intramuscularly to children aged 5 to 17 months living in a malaria-endemic area:

Amended (04 July 2006)

- to assess safety up to 19 months post Dose 1 when vaccinees are stratified by age at the time of Dose 1.
- to assess antibody responses to the CS antigen and the HBs antigen up to 19 months post Dose 1 when vaccinees are stratified by age at the time of Dose 1.
- to evaluate T-cell-mediated immune response (CMI) to CS antigen up to 19 months post Dose 1

Study design

- Experimental design: Phase II, controlled, partially blind (blind to vaccine administration, open to vaccination schedule), randomized (1: 1: 1: 1: 1: 1) trial with six treatment groups ***at each study site***.

Amended (04 July 2006)

Vaccine	Schedule (Months)	Number of children to be enrolled	Estimated number evaluable
RTS,S/AS01E (0.5 mL dose)	0, 1, 2	90	75
Rabies vaccine (0.5 mL dose)[a]	0, 1, 2	~~90~~ ***45***	~~75~~ ***40***
RTS,S/AS02D (0.5 mL dose)[b]	***0, 1, 2***	***45***	***40***
RTS,S/AS02D (0.5 mL dose)	0, 1, 7	90	75
RTS,S/AS01E (0.5 mL dose)	0, 1, 7	90	75
RTS,S/AS02D (0.5 mL dose)	0, 1	90	75
RTS,S/AS01E (0.5 mL dose)	0, 1	90	75

[a] The Rabies vaccine will only be used at Kintampo – KHRC
[b] Enrollment will occur at Kumasi – KCCR/SMS

Amended (04 July 2006)

- ***In the Kintampo Health Research Center (KHRC), on the 0, 1, 2-schedule, subjects will receive the Rabies vaccine as a control. In the Kumasi Center for Collaborative Research / School of Medical Sciences (KCCR/SMS), on the 0, 1, 2-schedule, subjects will receive the RTS,S/AS02D experimental vaccine as an active comparator.*** Randomization to each of the ***other*** study groups will be balanced between the two study sites.

Amended (04 July 2006)

- Healthy male and female children aged 5 to 17 months will be screened; those determined to be eligible, based on the inclusion and exclusion criteria, will be enrolled in the study.
- Only those children whose parents/guardians provide written proof that the child has completed a 3 dose regimen of a licensed Hepatitis B vaccine in infancy will be included in this trial of an experimental Hepatitis B vaccine.
- Route of administration: all vaccines will be administered by the intramuscular route to the left deltoid.
- Each child will be observed for at least 60 minutes after vaccination to evaluate and treat any acute adverse events (AEs).
- There will be a 7-day follow-up period for solicited AEs post-vaccination: Day 0 evaluation will be carried out by the study physician at the study center. Subsequently, trained field workers will visit the children to solicit AEs on Days 1 to 6 after each vaccination (evaluation on Day 6 post Dose 1 will be carried out by the study physician at the study center).
- There will be a 30-day (day of vaccination and 29 subsequent days)

follow-up after each vaccine dose for reporting unsolicited symptoms.

- Serious adverse events (SAEs) will be recorded throughout the study period. Prior to vaccination, any SAEs due directly to study procedures will be captured. All SAEs will be captured, beginning with the administration of the first dose and ending 19 months after Dose 1 of study vaccines. After Month 10 (the primary study phase) of the trial, all enrolled children will be visited at home monthly by field workers until study conclusion to ensure complete identification of all SAEs.

- Three unblinded safety reports will be produced for the DSMB on safety information collected on vaccinees. The first safety report will be produced on data collected on the first 90 vaccinees on a 0, 1, 2-schedule to complete 7 days of follow-up post Dose 1. A second and third DSMB report will be produced on data from these children up to the time that they complete 7 days of follow-up post Dose 2 and post Dose 3 respectively.

- Blood samples for assessment of hepatic and renal function will be collected at all timepoints at which blood samples for immunogenicity are taken (see below). In addition, blood will be taken from the first 100 subjects who present for follow-up at Day 6 post Dose 1 for inclusion in the first DSMB safety review.

- Anti-CS antibody titers, anti-HBs antibody titers and CMI response will be determined at the time points indicated in the table below.

	PRIMARY STUDY PHASE												EXTENDED FOLLOW-UP PHASE								
Study Month	Scr	0	1	2	3	4	5	6	7	8	9	10	11	12	13	14	15	16	17	18	19
0, 1-month vaccination schedule																					
CS/HBs	**HBs**			**HBs**																	**HBs**
	**CS**			**CS**					**CS**			**CS**									**CS**
CMI				**CMI**																	**CMI**
VACC		**X**	**X**																		
0, 1, 2-month vaccination schedule																					
CS/HBs	**HBs**				**HBs**																**HBs**
	**CS**				**CS**				**CS**			**CS**									**CS**
CMI					**CMI**																**CMI**
VACC	**X**	**X**	**X**	**X**																	
0, 1, 7-month vaccination schedule																					
CS/HBs	**HBs**									**HBs**											**HBs**
	**CS**								**CS**	**CS**		**CS**									**CS**
CMI										**CMI**											**CMI**
VACC	**X**	**X**	**X**						**X**												

Scr: Screening VACC: Vaccination

Amended (04 July 2006)

- This study will not capture cases of malaria for the analysis of efficacy. All cases of malaria presenting during the unsolicited period will be captured as unsolicited events. Cases at anytime during the study that meet the criteria for a SAE will be reported as SAEs.
- The access of the study population to Insecticide Treated Bednets, HIV voluntary counseling and testing and HIV antiretroviral therapy according to national recommendations will be facilitated, in collaboration with the government services.
- The organization of the dates of experimental vaccination will take into account the EPI vaccination with measles and yellow fever vaccination at nine months of age and allow a minimum of 14 days period of separation between the study vaccines and the Measles and Yellow Fever vaccines. Any EPI vaccines administered during the duration of the study will be documented in the CRF.
- Final analysis will be carried out on data collected up to ten months post Dose 1. An addendum safety analysis will be performed on data collected up to the end of the study, 19 months post Dose 1.
- Data collection: conventional Case Report Form (CRF).

Number of subjects

540 subjects will be enrolled. It is expected that approximately ~~450~~ ***455*** subjects will be evaluable at study end.

Amended (04 July 2006)

Primary endpoints

Safety

- Occurrence of SAEs from the time of first vaccination until ten months post Dose 1.

Secondary endpoints

Safety & Reactogenicity

- Occurrence of solicited general and local reactions over a 7-day follow-up period (day of vaccination and 6 subsequent days) after each vaccination.
- Occurrence of unsolicited symptoms after each vaccination over a 30-day follow-up period (day of vaccination and 29 subsequent days).

Immunogenicity

- Anti-CS antibody titers prior to vaccination until ten months post Dose 1.
- Anti-HBs antibody titers prior to vaccination and one month post final vaccine dose

Tertiary endpoints

Safety

- Occurrence of SAEs from ten to 19 months post Dose 1.

Immunogenicity

- Anti-CS antibody titers at 19 months post Dose 1.
- Anti-HBs antibody titers at 19 months post Dose 1.

Exploratory endpoints

Safety

- Occurrence of SAEs from ten to 19 months post Dose 1 when vaccinees are stratified by age at the time of Dose 1.
- Occurrence of solicited general and local reactions over a 7-day follow-up period (day of vaccination and 6 subsequent days) after each vaccination when vaccinees stratified by age at the time of Dose 1.
- Occurrence of unsolicited symptoms after each vaccination over a 30-day follow-up period (day of vaccination and 29 subsequent days) when vaccinees stratified by age at the time of Dose 1.

Immunogenicity

- Anti-HBs antibody titers until 19 months post Dose 1 when vaccinees stratified by age at the time of Dose 1.
- Anti-CS antibody titers until 19 months post Dose 1 when vaccinees stratified by age at the time of Dose 1.

Cell-mediated immunity

- Frequency of CS-specific T-cells one month post final dose and at Month 19.

TABLE OF CONTENTS

PAGE

SYNOPSIS **16**

LIST OF ABBREVIATIONS **28**

GLOSSARY OF TERMS **31**

1. INTRODUCTION **35**
1.1. Malaria 35
1.2. Hepatitis 35
1.3. RTS,S candidate vaccine 36
1.3.1. AS02 and AS01 adjuvants 36
1.4. The RTS,S/AS02 candidate malaria vaccine; key clinical efficacy, safety and immunogenicity data 37
1.5. The RTS,S/AS01B candidate malaria vaccine; preliminary clinical safety, efficacy and immunogenicity data 39
1.6. Rationale for the study design 42
1.6.1. Rationale for the use of Rabies vaccine as a control 43
1.6.2. Rationale for CMI testing 43

2. OBJECTIVES **44**
2.1. Primary objective 44
2.2. Secondary objectives 44
2.3. Tertiary objectives 45
2.4. Exploratory Objectives 45

3. STUDY DESIGN OVERVIEW **46**

4. STUDY COHORT **50**
4.1. Kintampo Health Research Centre and Kumasi Centre for Collaborative Research/School of Medical Sciences 50
4.1.1. Kumasi Centre for Collaborative Research/School of Medical Sciences (KCCR/SMS) 50
4.1.1.1. KCCR/SMS: Study Area Geography, Population & Malaria Epidemiology 51
4.1.1.2. Agogo Presbyterian Hospital 51
4.1.2. Kintampo Health Research Centre (KHRC) 51
4.1.2.1. KHRC: Study Area Geography, Population & Malaria Epidemiology 52
4.1.2.2. Kintampo District Hospital (KDH) 52
4.1.3. Komfo Anokye Teaching Hospital (KATH), Kumasi 53
4.2. HIV Services 53
4.2.1. Treatment of HIV-infected children 53
4.3. Inclusion criteria 53
4.4. Exclusion criteria for enrolment 54
4.5. Elimination criteria during the study 55
4.6. Contraindications to subsequent vaccination 55
4.6.1. Indications for deferral of vaccination 55
4.6.2. Absolute contraindications to further vaccination 56

5. CONDUCT OF STUDY 56
5.1. Ethics and regulatory considerations 56
5.1.1. Institutional Review Board/Independent Ethics Committee (IRB/IEC) 56
5.1.2. Informed consent 57
5.1.3. Safety monitoring plan 60
5.1.3.1. Data Safety Monitoring Board (DSMB) 60
5.1.3.1.1. Data Reviewed by the DSMB 60
5.1.3.2. Local Safety Monitor (LSM) 61
5.1.3.2.1. Data Reviewed by the LSM 61
5.1.3.3. Safety monitoring reports 61
5.1.3.4. Process for the suspension of progression to the next sequential vaccine dose 62
5.1.3.5. Process if the trial is suspended 63
5.1.4. Exposure to rabies 63
5.1.5. Cross over immunization with Rabies vaccine 63
5.2. Storage of study documentation at investigator's sites 64
5.3. Recruitment/Screening 64
5.3.1. Community information 64
5.3.2. Screening of volunteers 64
5.4. Vaccination process 65
5.5. Home follow-up visits for assessment of reactogenicity (7-day follow-up period) 66
5.6. Monitoring of hematological and biochemical laboratory parameters 67
5.7. Surveillance for SAEs (all subjects) 67
5.7.1. At health facilities 67
5.7.2. In the community 68
5.8. Provision of health care 68
5.8.1. Management and treatment of malaria in all subjects 69
5.8.2. Measles and Yellow Fever vaccination 69
5.8.3. Referring of excluded volunteers to medical care 69
5.9. Subject identification 69
5.10. Outline of study procedures 71
5.11. Detailed description of study stages/visits 75
5.11.1. Study visits for children to receive RTS,S/AS01E or RTS,S/AS02D on a 0, 1-month schedule 78
5.11.2. Study visits for children to receive RTS,S/AS01E or Rabies vaccine *or RTS,S/AS02D* on a 0, 1, 2-month schedule 83
5.11.3. Study visits for children to receive RTS,S/AS01E or RTS,S/AS02D on a 0, 1, 7-month schedule 89
5.12. Sample handling and analysis 94
5.12.1. Treatment and storage of biological samples 94
5.12.2. Laboratory assays 95
5.12.3. Serology plan 95

6. INVESTIGATIONAL PRODUCTS AND ADMINISTRATION 96
6.1. Study vaccines 96
6.1.1. RTS,S/AS01E vaccine 96
6.1.1.1. RTS,S antigen presentation: 97
6.1.1.2. AS01E adjuvant: 97
6.1.2. RTS,S/AS02D vaccine 97

6.1.2.1. RTS,S antigen presentation: 97
6.1.2.2. AS02D adjuvant: 97
6.1.3. Rabipur Rabies vaccine 97
6.2. Dosage and administration 97
6.2.1. RTS,S/AS01E 98
6.2.2. RTS,S/AS02D 98
6.2.3. Rabipur Rabies vaccine 98
6.3. Storage 98
6.4. Treatment allocation and randomization 99
6.4.1. Randomization of supplies 99
6.4.2. Randomization of subjects 99
6.5. Method of blinding and breaking the study blind 100
6.6. Replacement of unusable vaccine doses 100
6.7. Packaging 101
6.8. Vaccine accountability 101
6.9. Concomitant medication/treatment 101

7. HEALTH ECONOMICS 102

8. ADVERSE EVENTS AND SERIOUS ADVERSE EVENTS 102
8.1. Definition of an adverse event 102
8.2. Definition of a serious adverse event 103
8.3. Clinical laboratory parameters and other abnormal assessments qualifying as adverse events and serious adverse events 104
8.4. Time period, frequency, and method of detecting adverse events and serious adverse events 104
8.4.1. Solicited adverse events 105
8.5. Evaluating adverse events and serious adverse events 106
8.5.1. Assessment of intensity 106
8.5.2. Assessment of causality 108
8.6. Follow-up of adverse events and serious adverse events and assessment of outcome 109
8.7. Prompt reporting of serious adverse events to GSK Biologicals 110
8.7.1. Time frames for submitting serious adverse event reports to GSK Biologicals 110
8.7.2. Completion and transmission of serious adverse event reports to GSK Biologicals 110
8.8. Regulatory reporting requirements for serious adverse events 112
8.9. Post-study adverse events and serious adverse events 113
8.10. Pregnancy 113
8.11. Treatment of adverse events 113

9. SUBJECT COMPLETION AND WITHDRAWAL 113
9.1. Subject completion 113
9.2. Subject withdrawal 114
9.2.1. Subject withdrawal from the study 114
9.2.2. Subject withdrawal from investigational product 114

10. DATA EVALUATION: CRITERIA FOR EVALUATION OF OBJECTIVES 115
10.1. Primary endpoint 115
10.2. Secondary endpoints 115
10.3. Tertiary endpoints 115

10.4. Exploratory Endpoints 115
10.5. Estimated sample size 116
10.5.1. Sample size for the primary safety endpoint 116
10.5.2. Sample size for the secondary immunogenicity endpoint 117
10.6. Study cohorts to be evaluated 117
10.6.1. Total Vaccinated Cohort 117
10.6.2. According to protocol (ATP) cohort for analysis of safety 117
10.6.3. According to protocol (ATP) cohort for analysis of immunogenicity 118
10.7. Derived and transformed data 118
10.8. Final analyses 118
10.8.1. Analysis of demographics/baseline characteristics 118
10.8.2. Analyses of safety 119
10.8.2.1. Primary endpoint 119
10.8.2.2. Secondary safety endpoints 119
10.8.3. Analyses of immunogenicity 119
10.8.3.1. Anti-CS antibodies 120
10.8.3.2. Anti-HBs antibodies 120
10.9. Annex analysis at study conclusion 120
10.9.1. Analyses of safety 120
10.9.2. Analyses of immunogenicity 120
10.9.2.1. Anti-HBs antibodies 121
10.9.2.2. Anti-CS antibodies 121
10.10. Exploratory Analyses 121
10.11. Planned interim analysis 121

11. ADMINISTRATIVE MATTERS 121

12. REFERENCES 121

APPENDICES

Appendix A World Medical Association Declaration of Helsinki 127

Appendix B Administrative Matters 131

Appendix C Overview of the Recruitment Plan 137

Appendix D Handling of Biological Samples Collected by the Investigator 139

Appendix E Shipment of Biological Samples 143

Appendix F Laboratory Assays 145

Appendix G Vaccine supplies, packaging and accountability 146

Appendix H Amendments and Administrative Changes to the Protocol 148

List of Abbreviations

AE	Adverse event
ALT	Alanine aminotransferase
anti-CS	Antibody to the *P. falciparum* circumsporozoite (CS) repeat domain
anti-HBsAg	Antibody to the hepatitis B surface antigen
ATP	According to protocol
CI	Confidence interval
CMI	Cell-mediated immunity
CRF	Case report form
CS	Circumsporozoite protein of *P. falciparum*
DTPw/Hib	Diphtheria, Tetanus, Pertussis (whole cell) and *Hemophilus influenzae* type B conjugate vaccine
DSMB	Data safety monitoring board
EIA	Enzyme immunosorbent assay
ELISA	Enzyme linked immunosorbent assay
EPI	Expanded program on immunization
FDA	Food and Drug Administration, United States
GCP	Good clinical practice
GMT	Geometric mean titer
GSK	GlaxoSmithKline
HBsAg	Hepatitis B surface antigen
HBV	Hepatitis B virus
Hep B	Hepatitis B
HIV	Human immunodeficiency virus
IB	Investigator’s brochure

ICF	Informed consent form
ICH	International Committee on Harmonization
IEC	Independent ethics committee
IFN-γ	Interferon gamma
IM	Intramuscular
IND	Investigational new drug
IRB	Institutional review board
IU	International unit
ITN	Insecticide-treated bednet
KCCR/SMS	Kumasi Centre for Collaborative Research, School of Medical Sciences
kg	Kilogram
KHRC	Kintampo Health Research Centre
LEP	Low egg passage
LLN	Lower limit of normal
LSM	Local safety monitor
MedDRA	Medical Dictionary for Regulatory Activities
mg	Milligram
mL	Milliliter
MPL®	3-deacylated monophosphoryl lipid A
MVI	Malaria Vaccine Initiative
P. falciparum	*Plasmodium falciparum*
PATH	Program for Appropriate Technology in Health
PFS	Pre-filled syringe
PI	Principal Investigator
PMCT	Prevention of Mother-to-Child Transmission

PPP	Pre-patent period
QS 21	'*Quillaja saponaria* 21': a triterpene glycoside purified from the bark of the soap bark tree, *Quillaja saponaria*
RAP	Report and Analysis Plan
RTS	Hybrid protein comprising HBs (hepatitis B surface antigen) and CSP portions
RTS,S	Particulate antigen, containing both RTS and HBs proteins
SAE	Serious adverse event
SOP	Standard operating procedure
SP	sulfadoxine-pyrimethamine
ULN	Upper limit of normal
VCT	Voluntary Counseling and Testing
VE	Vaccine efficacy
WRAIR	Walter Reed Army Institute of Research

Glossary of Terms

Adverse event:	Any untoward medical occurrence in a patient or clinical investigation subject, temporally associated with the use of a medicinal product, whether or not considered related to the medicinal product.<br><br>An AE can therefore be any unfavorable and unintended sign (including an abnormal laboratory finding), symptom, or disease (new or exacerbated) temporally associated with the use of a medicinal product. For marketed medicinal products, this also includes failure to produce expected benefits (i.e. lack of efficacy), abuse or misuse.
Severe Adverse Event	A serious adverse event (SAE) is any untoward medical occurrence that:<br><br>a. results in death;<br>b. is life-threatening;<br>c. requires hospitalization or prolongation of existing hospitalization;<br>d. results in disability/incapacity;<br>e. medical or scientific judgment should be exercised in deciding whether reporting is appropriate in other situations, such as important medical events that may not be immediately life-threatening or result in death or hospitalization but may jeopardize the subject or may require medical or surgical intervention to prevent one of the other outcomes listed in the above definition. These should also be considered serious. Examples of such events are invasive or malignant cancers, intensive treatment in an emergency room or at home for allergic bronchospasm, blood dyscrasias or convulsions that do not result in hospitalization.<br><br>A full definition of the events that constitute SAEs can be found in Section 8.2).

Blinding:	A procedure in which one or more parties to the trial are kept unaware of the treatment assignment in order to reduce the risk of biased study outcomes. In a single-blind trial, the investigator and/or his staff are aware of the treatment assignment but the subject is not. In an observer-blind study, the subject and the study personnel involved in the clinical evaluation of the subjects are blinded while other study personnel may be aware of the treatment allocation. When the investigator and sponsor staff who are involved in the treatment or clinical evaluation of the subjects and review/analysis of data are also unaware of the treatment assignments, the study is double blind. Partially blind is to be used for study designs with different blinding levels between different groups, e.g. double blinded consistency lots which are open with respect to the control group. The level of blinding is maintained throughout the conduct of the trial, and only when the data are cleaned to an acceptable level of quality will appropriate personnel be unblinded or when required in case of a serious adverse event.
Central Study Coordinator:	An individual assigned by and centrally located at GSK Biologicals at Rixensart who is responsible for assuring proper conduct of a clinical study.
Data Safety Monitoring Board (DSMB):	The DSMB is an independent committee appointed to oversee ethical and safety aspects of the conduct of the study. See Section 5.1.3.1 for a full overview of the role and structure of the DSMB.
Eligible:	Qualified for enrollment into the study based upon strict adherence to inclusion/exclusion criteria.
eTrack	GSK's clinical trials tracking tool.
Evaluable:	Meeting all eligibility criteria, complying with the procedures defined in the protocol, and, therefore, included in the according-to-protocol (ATP) analysis (see Sections 4.3, 4.4 and 10.5.2 for details on criteria for evaluability).
Investigational product:	A pharmaceutical form of an active ingredient or placebo being tested or used as a reference in a clinical trial, including a product with a marketing authorization when used in a way different from the approved form, or when used for an unapproved indication, or when used to gain further information about an approved use. The investigational products for this study are RTS,S/AS02D, RTS,S/AS01E and Rabipur

Local Safety Monitor (LSM):	The overall role of the Local Safety Monitor, an experienced physician based in-country, will be to support the study investigators and to act as a link between the investigators and the Data Safety Monitoring Board (DSMB) (see Section 5.1.3.2 for further details on the LSM).
Medical Monitor:	An individual medically qualified to assume the responsibilities of the sponsor (GSK Biologicals) especially in regards to the ethics, clinical safety of a study and the assessment of adverse events.
Pre-patent Period (PPP)	The time in days between experimental sporozoite challenge and first detection of parasitemia by peripheral blood thick smear.
Protocol amendment:	ICH defines a protocol amendment as: "A written description of a change(s) to or formal clarification of a protocol". GSK Biologicals further details this to include a change to an approved protocol that affects the safety of subjects, scope of the investigation, study design, or scientific integrity of the study.
Protocol administrative change:	A protocol administrative change addresses changes to only logistical or administrative aspects of the study.<br><br>N.B. Any change that falls under the definition of a protocol amendment (e.g. a change that affects the safety of subjects, scope of the investigation, study design, or scientific integrity of the study) MUST be prepared as an amendment to the protocol.
Randomization:	Process of random attribution of treatment to subjects in order to reduce bias of selection.
Solicited adverse event:	Adverse events (AEs) to be recorded as endpoints in the clinical study. The presence/occurrence/intensity of these events is actively solicited from the subject or an observer during a specified post-vaccination follow-up period.
Study Monitor:	An individual assigned by the sponsor who is responsible for assuring proper conduct of a clinical study.
Subject:	Term used throughout the protocol to denote an individual that has been contacted in order to participate in the clinical study, either as a recipient of the investigational product(s) or as a control.
Treatment:	Term used throughout the clinical study to denote a set of investigational products or marketed products intended to be administered to a subject, identified by a unique number, according to the study randomization or treatment allocation.

Treatment number:	A unique number identifying a treatment to a subject, according to the study randomization or treatment allocation.
Unsolicited adverse event:	Any adverse event (AE) reported in addition to those solicited during the clinical study. Also any "solicited" symptom with onset outside the specified period of follow-up for solicited symptoms will be reported as an unsolicited adverse event.

1. INTRODUCTION

1.1. Malaria

Four species of the *Plasmodium* protozoan parasite are the etiologic agents of malaria in humans (*P. falciparum*, *P. vivax*, *P. ovale* and *P. malariae*). Of these four parasites, *P. falciparum* is the major cause of severe morbidity and mortality.

There can be no doubt of the importance of *P. falciparum* malaria as a major cause of human suffering and economic drain across sub-Saharan Africa [Breman 2001a; Gallup 2001]. In this region, it causes the deaths of between 0.5 and 2.0 million children every year and is the most common reason for admission to hospital, leading each year to about 300 million clinical episodes in children under five years [Breman 2001a].

The incidence of malaria in much of Africa is increasing for a variety of reasons: changes in agricultural practices, armed conflicts, migration of refugees, increasing drug resistance to conventional anti-malarial drugs, and insecticide resistance of the anopheline mosquito vectors. It is estimated that without effective control the number of cases of clinical malaria will more than double over the next 20 years. The burden of malaria at the country level correlates closely with the rate of economic development even after adjustment for confounding factors, indicating that malaria is an important constraint on economic progress [Breman 2001b].

Clinical manifestations of *P. falciparum* disease appear as a result of the parasite infection of the red blood cell (RBC). Initial symptoms may include fever, chills, headache, joint and muscle pain, sweating, and vomiting. Acute complications may result from hemolysis leading to anemia and the propensity of infected RBCs to become adhesive and to be sequestered in capillaries thus causing local inflammatory reactions and damage to vital organs, leading to cerebral, hepatic, renal or pulmonary malaria. In *P. falciparum* malaria, an untreated acute attack can progress very rapidly and death may occur within a short timeframe.

Efforts to develop vaccines that target each stage of the parasite life cycle, to identify protective antigens and to understand the nature of the protective immune responses have been ongoing for the past three decades. The approach of GlaxoSmithKline (GSK) Biologicals has been to focus on vaccines that target the free sporozoite and intra-hepatic stages of the parasites (i.e. the pre-erythrocytic stages).

1.2. Hepatitis

Hepatitis B is an infection of the liver due to hepatitis B virus (HBV); it is an important public health problem across the developing world. World-wide approximately 350 million people carry HBV and about 1 million chronic carriers die annually [Vryheid 2001]. The likelihood of the infection becoming chronic is dependent upon the age at infection: 90% if infected in infancy, 30% to 50% if infected between the ages of 1 to 4 years, and low in adulthood. For those that become chronically infected during childhood

the risk of death from HBV-related liver cancer or cirrhosis in adult life is approximately 25% [World Health Organization 2003].

1.3. RTS,S candidate vaccine

GSK Biologicals and the Walter Reed Army Institute of Research (WRAIR) are developing a candidate antigen against malaria caused by *P. falciparum*: RTS,S. The vaccine consists of sequences of the circumsporozoite (CS) protein and the hepatitis B surface antigen (HBsAg) adjuvanted with AS02 (proprietary oil-in-water emulsion formulated with MPL® and Stimulon® QS21 immunostimulants) or AS01 (liposome formulation with MPL and QS21 immunostimulants).

The HBsAg contained in the RTS,S candidate malaria vaccine is encoded by the hepatitis B virus S protein gene that is identical to the gene used to express HBsAg in GSK Biologicals' Engerix-B® vaccine against hepatitis B. As a result, vaccines containing RTS,S also provide protection against hepatitis B.

In parallel to the continued development of RTS,S/AS02 in children in endemic countries, GSK Biologicals and WRAIR have continued to pursue strategies to improve the vaccine efficacy (VE) and duration of efficacy. One such strategy is the combination of the RTS,S antigen with the AS01 adjuvant. Both the AS02 and AS01 adjuvant formulations have a number of similar key components (Table 1).

Table 1 Formulations of RTS,S

Formulation	RTS,S (µg)	MPL (µg)	QS21 (µg)	Volume (mL)	Key clinical trials
RTS,S/AS02A (0.5 mL dose)	50	50	50	0.5	Efficacy demonstrated in adults in an endemic area (Malaria-005[a])
RTS,S/AS02A (0.25 mL dose)	25	25	25	0.25	Efficacy demonstrated in children in an endemic area (Malaria-026[b])
RTS,S/AS02D (0.5 mL dose)	25	25	25	0.5	Bridged to RTS,S/AS02A (0.25 mL dose) (Malaria-034[c])
RTS,S/AS01B (0.5 mL dose)	50	50	50	0.5	Efficacy demonstrated in malaria-naïve adults (Malaria-027[d])
RTS,S/AS01E (0.5 mL dose)	25	25	25	0.5	Proposed pediatric formulation

a GSK data on file, Malaria-005, Clinical Study Protocol, 1997; Bojang, 2001
b GSK data on file, Malaria-026, Clinical Study Report, 2004; Alloueche, 2003
c GSK data on file, Malaria-034, Clinical Study Report, 2004
d GSK data on file, Malaria-027, Clinical Study Protocol, 2003

1.3.1. AS02 and AS01 adjuvants

The GSK proprietary adjuvant system 2 (AS02) is composed of a proprietary oil-in-water emulsion and the immunostimulants Stimulon® QS21 (a triterpene glycoside purified from the bark of *Quillaja saponaria*) and MPL®. RTS,S/AS02D is composed of the same active constituents in the same quantities as in a 0.25 mL dose of RTS,S/AS02A, but is formulated to supply a 0.5 mL dose. AS01B utilizes liposomes in place of proprietary oil-in-water emulsion. RTS,S/AS01E, the pediatric formulation of RTS,S/AS01B, is

composed of the same active constituents, but with half the quantities as in RTS,S/AS01B and is administered as a 0.5 mL dose.

1.4. The RTS,S/AS02 candidate malaria vaccine; key clinical efficacy, safety and immunogenicity data

In the clinical trial setting to date, the RTS,S/AS02A candidate vaccine has been administered to both malaria-naïve adult subjects (subjects who live in countries where there is no naturally occurring malaria transmission) and naturally exposed adults and children (subjects who live in countries where malaria transmission occurs naturally). The numbers of doses of RTS,S-containing vaccines and the number of recipients is tabulated in Table 2. A comprehensive summary of the results of reported trials to date can be found in the Malaria Vaccine Investigator Brochure 2005 [GSK data on file].

Table 2 Approximate number of doses of RTS,S/AS02A administered to date with number of recipients

Subject population	Recipients	Doses administered
Malaria-naïve adults [a]	247	596
Naturally-exposed adults [b]	266	842
Naturally-exposed children [b]	1292	3746

a subjects who took part in studies conducted in the USA and Belgium, where there is no naturally occurring transmission of malaria
b subjects who took part in studies conducted in malaria-endemic countries in Africa.

Early clinical development of the RTS,S malaria candidate vaccine was initiated in studies in malaria-naïve adults in collaboration with the WRAIR in which confirmation of the efficacy, safety and immunogenicity of the RTS,S/AS02A vaccine formulation was demonstrated [Stoute 1997; Kester 2001]. Two doses of RTS,S/AS02A (0.5 mL) provided protection to 37.8% healthy non-immune volunteers against homologous sporozoite challenge (pooled results for WRMAL-004 [GSK data on file] & -005 [GSK data on file]); 3 doses demonstrated protection of 43.2% of subjects (pooled results for WRMAL-004 & Malaria-012 [GSK data on file]). In subjects not protected, the prepatent period (PPP) was significantly prolonged in the RTS,S/AS02A group compared to control (WRMAL-004, WRMAL-005 & Malaria-012). Protective efficacy was low following re-challenge six months after Dose 3 of RTS,S/AS02A, but a statistically significant difference between PPP for vaccinees compared to infectivity control was observed (WRMAL-003 [GSK data on file]). A strong humoral immune response to the RTS,S/AS02A vaccine in terms of anti-CS and anti-HBs antibodies was demonstrated in all the adult studies in malaria-naïve individuals. Evaluations of the CMI response showed consistently that administration of RTS,S/AS02A induced strong cellular Th1 T-cell responses, specific to the vaccine antigen [Lalvani 1999; Stoute 1998; Epstein 2004; Sun 2003].

The RTS,S/AS02A vaccine progressed to evaluation in subjects under conditions of natural transmission. In adult males from The Gambia, VE against infection adjusted for covariates was 71% (95% CI: 46 to 85; $p < 0.001$) during the first 2 months and 34%

(95% CI: 8.0 to 53, p=0.014) for the entire 15 week surveillance period (Malaria-005 [GSK data on file]). VE, adjusted for covariates, following a booster dose given during a second year malaria season was 47% (95% CI: 3 to 71; p=0.039). Protection was not limited to the NF54 parasite genotype from which the vaccine was derived [Alloueche 2003]. Following review of SAEs and safety surveillance over approximately 5 years, no safety signal was apparent (Malaria-016, -017 & -018 [GSK data on file]). A strong humoral immune response to the RTS,S/AS02A vaccine in terms of anti-CS and anti-HBs antibodies was demonstrated. Overall, the kinetics of the humoral immune response induced by vaccination with RTS,S/AS02A were similar in malaria-naïve and experienced populations, while the absolute GMT values appeared to be higher in malaria-naïve volunteers. The vaccine induced and boosted Th1-like cellular immunity to several T-cell epitopes in a population naturally exposed to malaria.

The RTS,S/AS02A candidate vaccine progressed to clinical evaluation in children. Two age de-escalation and dose comparison trials (which compared doses with 10 µg, 25 µg and 50 µg of antigen corresponding to volume fractions of RTS,S/AS02A: 0.1 mL, 0.25 mL and 0.5 mL respectively) enrolled a total of 225 children aged 1 to 11 years from The Gambia in which a similar pattern and intensity of reactogenicity following vaccination with RTS,S/AS02A to that of previous studies in semi-immune adults was demonstrated (Malaria-015 [GSK data on file] & -020 [GSK data on file]). RTS,S/AS02A was highly immunogenic for both anti-CS and anti-HBs antibodies, irrespective of pre-vaccination HBs serostatus. From these trials, the 0.25 mL dose was selected due to equivalent immunogenicity and slightly less reactogenicity in the 0.25 mL group compared to the 0.5 mL group; immunogenicity was consistently lowest in the 0.1 mL group. The safety and immunogenicity of the RTS,S/AS02A 0.25 mL dose was further confirmed in another study, Malaria-025 [GSK data on file], conducted in 1 to 4 year old children in Mozambique.

Subsequently, a large safety, immunogenicity and efficacy trial in children aged 1 to 4 years from an area of high transmission was conducted in Mozambique, enrolling a total of 2022 subjects (Malaria-026 [GSK data on file]; [Alonso 2004]; [Alonso 2005]). In this study, 3 doses of RTS,S/AS02A (0.25 mL dose) were administered according to a 0, 1, 2-month schedule to approximately 1000 children. The primary endpoint of this trial investigated vaccine efficacy against the onset of clinical malaria disease. Up to six months post Dose 3, the VE determined as the time to the first clinical episode (after adjustment for covariates) was 29.9% (95% CI: 11.0 to 44.8; p=0.004). For the entire study period, up to 18 months post Dose 3, VE for the same endpoint was 32.8% (95% CI: 20.1 to 43.4; p=0.0001).

The trial also offered the opportunity to investigate VE against multiple attacks of malaria and severe malaria. Against multiple attacks, VE was 27.4% (95% CI: 6.2 to 43.8; p=0.014) up to six months post Dose 3. For the entire study period, VE was 32.4% (95% CI: 17.6 to 44.5; p=0.0001). Against severe malaria, VE was 57.7% (95% CI: 16.2 to 80.6; p=0.019) over six months post Dose 3: for the entire study period VE was 48.6% (95% CI 12.3 to 71.0; p=0.02).

The proportion of children experiencing a SAE was similar in the RTS,S/AS02A and control groups; no SAE was judged to be related to vaccination. No difference in the

pattern of morbidity notified as SAEs was observed between recipients of RTS,S/AS02A and control vaccines; the pattern of morbidity was similar to that previously observed at the study site and described in the region. At least 97% of recipients of RTS,S/AS02A were seropositive for anti-CS antibodies and at least 97% of recipients of RTS,S/AS02A were seroprotected for anti-HBs antibodies 18 months post Dose 3 [Alonso, 2005].

In parallel, a 0.5 mL variant of the 0.25 mL dose of RTS,S/AS02A (RTS,S/AS02D) was developed for compatibility with standard auto-disable EPI syringe. RTS,S/AS02D, was shown in children aged 3 to 5 years in a malaria-endemic region of Mozambique to be safe and exhibit non-inferior immunogenicity to RTS,S/AS02A (0.25 mL dose) (Malaria-034 [GSK data on file]); no SAE was reported in recipients of RTS,S/AS02D; non-inferiority of the anti-CS and anti-HBs antibody responses induced by the RTS,S/AS02D formulation as compared to the RTS,S/AS02A (0.25 mL dose) formulation was demonstrated. RTS,S/AS02D has progressed to assessment in infants. In the trial Malaria-038 the safety, efficacy and immunogenicity of RTS,S/AS02D is being assessed when it is administered to Mozambican infants two weeks after the administration of a dose of DTPw/Hib. In the Malaria-040 trial, due to start in April 2006, safety, efficacy and immunogenicity of RTS,S/AS02D will be assessed in Tanzanian infants when it is coadministered (administered on the same day) as DTPw/Hib.

1.5. The RTS,S/AS01B candidate malaria vaccine; preliminary clinical safety, efficacy and immunogenicity data

Recent preliminary results of a challenge study conducted in healthy malaria-naïve adults have been encouraging (Malaria-027 [GSK data on file]), indicating that RTS,S/AS01B may be more efficacious than RTS,S/AS02A. In this double-blind**,** randomized Phase I/IIa human challenge study, the safety, reactogenicity, immunogenicity and preliminary efficacy after early sporozoite challenge and rechallenge, of RTS,S/AS01B and RTS,S/AS02A were assessed. Two sequential cohorts of approximately 52 subjects each were enrolled; each cohort was evenly divided into 2 groups receiving either the RTS,S/AS01B or RTS,S/AS02A vaccine (up to 48 infectivity controls for challenge and re-challenge phases were additionally enrolled). Subjects were vaccinated at 0, 1, 2-months followed by a challenge 14 to 30 days after Dose 3. Protected individuals were invited to be rechallenged approximately 6 months after Dose 3 to evaluate persistence of efficacy. To date, results for Cohort 1 are available [GSK data on file].

In Cohort 1, following primary challenge, the point efficacy of VE was significantly greater in both vaccine groups compared to infectivity control. Although not statistically significant, VE was higher in the RTS,S/AS01B group compared to RTS,S/AS02A (58.8% [95% CI: 32.1, 81.6] vs 37.5% [95% CI: 11.8, 59.4], p=0.2085). In non-protected subjects, the mean time to infection (pre-patent period; PPP) following challenge was similar in both vaccine groups and longer than in the infectivity control.

Following re-challenge, 2/5 subjects (40.0%) in the RTS,S/AS01B group and 4/5 subjects (80.0%) in the RTS,S/AS02A group were infected; all infectivity control subjects became infected. There was a trend towards greater VE in the RTS,S/AS01B group compared to infectivity control (p=0.061) and VE was greater when compared to RTS,S/AS02A (60.0% [95% CI: 3.2, 95.1] vs 20.0% [95% CI: -60.2, 71.6], respectively). The PPP in

non-protected subjects was longer in the RTS,S/AS01B group compared RTS,S/AS02A; PPP was longer in both vaccine groups compared to control. Anti-CS antibody responses were greater in recipients of RTS,S/AS01B than of RTS,S/AS02A; anti-CS CD4+ T-cell responses were stronger in the RTS,S/AS01B group compared to RTS,S/AS02A.

The reactogenicity and safety profile of RTS,S/AS01B was comparable to RTS,S/AS02A (refer to Table 3 and Table 4). The overall incidence of AEs (both solicited and unsolicited) was comparable in the RTS,S/AS01B group (following 88.2% of doses) and the RTS,S/AS02A group (following 91.9% of doses). Grade 3 AEs (solicited and unsolicited) were reported less frequently in the RTS,S/AS01B group (following 17.6% doses) compared to the RTS,S/AS02A group (following 28.4% of doses). Only 10 solicited/unsolicited AEs (following < 0.5% of all doses administered) were reported during days 5 to 7 of the follow-up period following vaccination.

Table 3 Incidence per dose of solicited local symptoms reported during the 7-day follow-up period, Malaria-027, subjects in Cohort 1 only (Total Cohort)

Group		RTS,S/AS02A					RTS,S/AS01B				
		N	n	%	95% CI		N	n	%	95% CI	
Pain	Any	74	60	81.1	70.3	89.3	68	53	77.9	66.2	87.1
	Grade 3	74	11	14.9	7.7	25.0	68	2	2.9	0.4	10.2
Redness	Any	74	23	31.1	20.8	42.9	68	20	29.4	19.0	41.7
	Grade 3	74	1	1.4	0.0	7.3	68	4	5.9	1.6	14.4
Swelling	Any	74	19	25.7	16.2	37.2	68	11	16.2	8.4	27.1
	Grade 3	74	0	0.0	0.0	4.9	68	2	2.9	0.4	10.2

Table 4 Incidence per dose of solicited general symptoms reported during the 7-day follow-up period, Malaria-027, subjects in Cohort 1 only (Total Cohort)

Group		RTS,S/AS02A					RTS,S/AS01B				
		N	n	%	95% CI		N	n	%	95% CI	
Fatigue	Any	74	37	50.0	38.1	61.9	68	25	36.8	25.4	49.3
	Related	74	31	41.9	30.5	53.9	68	23	33.8	22.8	46.3
	Grade 3/rel	74	2	2.7	0.3	9.4	68	3	4.4	0.9	12.4
Headache	Any	74	28	37.8	26.8	49.9	68	22	32.4	21.5	44.8
	Related	74	22	29.7	19.7	41.5	68	19	27.9	17.7	40.1
	Grade 3/rel	74	0	0.0	0.0	4.9	68	2	2.9	0.4	10.2
Malaise	Any	74	27	36.5	25.6	48.5	68	22	32.4	21.5	44.8
	Related	74	25	33.8	23.2	45.7	68	21	30.9	20.2	43.3
	Grade 3/rel	74	2	2.7	0.3	9.4	68	1	1.5	0.0	7.9
Myalgia	Any	74	19	25.7	16.2	37.2	68	15	22.1	12.9	33.8
	Related	74	15	20.3	11.8	31.2	68	15	22.1	12.9	33.8
	Grade 3/rel	74	0	0.0	0.0	4.9	68	2	2.9	0.4	10.2
GI	Any	74	12	16.2	8.7	26.6	68	14	20.6	11.7	32.1
	Related	74	7	9.5	3.9	18.5	68	12	17.6	9.5	28.8
	Grade 3/rel	74	1	1.4	0.0	7.3	68	0	0.0	0.0	5.3
Arthralgia	Any	74	10	13.5	6.7	23.5	68	14	20.6	11.7	32.1
	Related	74	7	9.5	3.9	18.5	68	13	19.1	10.6	30.5
	Grade 3/rel	74	0	0.0	0.0	4.9	68	1	1.5	0.0	7.9
Fever	Any	74	10	13.5	6.7	23.5	68	12	17.6	9.5	28.8
	Related	74	8	10.8	4.8	20.2	68	12	17.6	9.5	28.8
	Grade 3/rel	74	0	0.0	0.0	4.9	68	0	0.0	0.0	5.3

N= number of administered doses; n/%= number/percentage of doses followed by at least one type of symptom
95%CI= Exact 95% confidence interval; Lower/Upper limits
rel=related
GI: Gastrointestinal

Pain was the most frequently reported solicited local AE in both the RTS,S/AS01B and RTS,S/AS02A groups (following 77.9% and 81.1% of doses, respectively), occurring with a similar frequency in each vaccine group. The incidence of Grade 3 pain was lower in the RTS,S/AS01B group compared to the RTS,S/AS02A group (following 2.9% and 14.9% of doses, respectively). Fatigue, headache and malaise were the most frequently reported solicited general AEs after vaccination in both groups. All Grade 3 solicited local events resolved within the 7 day follow-up period.

Few Grade 3 solicited general AEs were reported (following ≤ 4.4% doses). The frequency of unsolicited AEs considered to be related to vaccination was similar in the RTS,S/AS01B and RTS,S/AS02A groups (following 22.1% and 17.6% of doses, respectively).

Two SAEs were reported during the study (tendon rupture, cerebral infarction), both in the RTS,S/AS01B group, neither of which was considered to be related to study vaccine.

1.6. Rationale for the study design

This trial will investigate the administration of the candidate malaria vaccines RTS,S/AS02D and RTS,S/AS01E. There are two main components to this investigation.

1. to describe the safety and reactogenicity profile of RTS,S/AS01E and RTS,S/AS02D when administered to 5-17 month old children

This trial is one of a series of age de-escalation steps with the aim of assessing whether RTS,S/AS01E and RTS,S/AS02~~A~~ ***D*** may be suitable for inclusion in an EPI vaccination program. The safety and reactogenicity profile of RTS,S/AS02A has already been described in a number of studies in children in Africa (see Section 1.4). RTS,S/AS01 has been developed as a potential improvement to the RTS,S/AS02 vaccines. Preliminary results from the Malaria-027 trial in adults indicate that the RTS,S/AS01B formulation may be more efficacious than RTS,S/AS02A while retaining its acceptable reactogenicity profile (refer to Section 1.5). One of this study's aims is to describe the safety and reactogenicity profile of the two vaccines in this younger age group. **Amended (04 July 2006)**

In addition, although RTS,S/AS01E is primarily being developed as a vaccine against malaria, the particles of RTS,S contain HBsAg and the vaccine induces high seroprotection rates against hepatitis B (see Section 1.4). Therefore it is logical to propose that, in malaria-endemic regions, RTS,S/AS01E replaces the existing hepatitis B vaccine in the infant regimen. However, co-administration of an RTS,S vaccine in infants of the EPI age range may be difficult, for safety reasons or interference in immunogenicity in case of vaccine co-administration. If that is the case, children of 5-17 months of age may still benefit from the RTS,S vaccination. This study will investigate safety of RTS,S vaccination in children recently vaccinated with three doses of a classical anti-HBV vaccine (HBsAg vaccine).

2. to evaluate alternative schedules for the administration of the candidate malaria vaccines

~~To date, RTS,S/AS02A has been extensively evaluated on a 0, 1, 2-month schedule in children between the ages of 1 and 4 years in Mozambique and found to provide good immunogenicity [GSK data on file, Malaria-025, GSK data on file, Malaria-034] and efficacy [GSK data on file, Malaria-026] with an acceptable safety profile. Because of this extensive prior evaluation, RTS,S/AS02D will not be assessed on a 0, 1, 2-schedule in this trial.~~ **Amended (04 July 2006)**

There are two reasons why the RTS,S/AS01E and RTS,S/AS02D candidate malaria and hepatitis B vaccines are being developed for delivery through the infant Expanded Program on Immunization (EPI) of the World Health Organization (WHO). Firstly, the vaccine is being developed to prevent severe malaria disease which occurs from about 4 months of age coinciding with the time that maternally-acquired immunity wanes [Snow 1993, Snow 1994]. Secondly the EPI has been highly successful at increasing the coverage of basic vaccines across the developing world.

While a 0, 1, 2 months schedule is commonly used throughout Africa for the administration of DTPw vaccine, it is not the only available option. The WHO's draft

global immunization strategies specifically recommend the characterization of optimal schedules for new vaccines [World Health Organization, 2005]. It is possible that other schedules may produce a better reactogenicity profile or better immunogenicity profile. This trial is designed to test the candidate malaria vaccine formulations under alternative schedules that can be integrated in the present EPI; a rapid immunization course following a 0,1-month schedule, a 3 dose 0, 1, 2 months schedule and an alternative 3-dose regimen following a 0, 1, 7-dose schedule: the visits on months 0 and 1 correspond to the first two dates of DTP vaccination under EPI, and the visit at Month 7 corresponds with the administration of Measles and/or Yellow Fever vaccine. The delay between administering Dose 2 and Dose 3 of vaccine may allow maturing of the immune system allowing for better stimulation of primed B-cells and T-cells.

1.6.1. Rationale for the use of Rabies vaccine as a control

Rabies vaccine has been chosen as the comparator because: 1) volunteers will benefit from receiving Rabies vaccine as rabid animals occur in the study area and 2) Rabies vaccine can be administered on a 0, 1, 2-month schedule.

Chiron's Rabipur® vaccine will be used. When the Rabipur Rabies vaccine is administered according to the recommended vaccination schedule (days 0, 7, 21), nearly 100% of subjects attain a protective titer. In two studies carried out in the US in 101 subjects, protective antibody titers > 0.5 IU/mL were obtained by day 28 in all subjects. In studies carried out in Thailand in 22 subjects, and in Croatia in 25 subjects, antibody titers of > 0.5 IU/mL were obtained by day 14 (injections on days 0, 7, 21) in all subjects [Dreesen, 1989; Nicholson, 1987; Vodopija, 1986; Wasi, 1986].

High antibody titers have also been demonstrated with off-label immunization with Rabies vaccines. Among participants in England, Germany, France and Belgium who received two vaccinations one month apart, nearly 100% of the participants developed specific antibody and the geometric mean titer for the group was 10 IU [Ajjan, 1978; Costy-Berger, 1978; Cox, 1976; Kuwert, 1978]. The proposed vaccination schedule of 0, 1, 2-months is therefore expected to be highly successful in conferring protective immunity against rabies among the control participants. However all parents/guardians will be advised that if their child is bitten or scratched by a dog or cat they must seek medical attention immediately.

1.6.2. Rationale for CMI testing

Neutralizing-antibody and cell-mediated immunity (CMI) responses are thought to be important immune effector mechanisms for protecting people vaccinated with RTS,S/AS02 and RTS,S/AS01. It is hypothesized that these responses act to limit hepatocyte invasion, destroy infected hepatocytes and/or limit intracellular parasite development.

RTS,S/AS02A has been shown to be a powerful inducer of antigen-specific humoral and CMI responses in preclinical and clinical studies [Malaria Investigator's Brochure 2005, GSK data on file]. Recent preliminary results of a challenge study conducted in healthy malaria-naïve adults (Malaria-027 [GSK data on file]) shows that RTS,S/AS01B induces

more potent CMI responses characterized predominantly by CD4+ type 1 responses and higher anti-CS antibody levels as compared to RTS,S/AS02A. This may be associated with increased vaccine efficacy on the part of RTS,S/AS01B; immunological monitoring performed during this trial revealed an association between protection against *P. falciparum* and CS-specific humoral and cellular response (Dr Kent Kester, WRAIR, personal communication, November 2005; publication in preparation).

We will describe CMI responses in children vaccinated with RTS,S/AS01E and RTS,S/AS02D administered on a 0, 1-month, 0, 1, 2-month and 0, 1, 7-month schedule in order to explore differences in cellular immunity induction by vaccine schedules, as this may translate into differences in vaccine efficacy. Recipients of Rabies vaccine will also be assessed to act as a control. Responses will be measured on cells taken one month post final vaccine dose and at Month 19.

2. OBJECTIVES

2.1. Primary objective

- For RTS,S/AS01E (on a 0, 1-month and a 0, 1, 2-month and a 0, 1, 7-month schedule) and RTS,S/AS02D (on a 0, 1-month, ***0, 1, 2-month*** and a 0, 1, 7-month schedule) when administered intramuscularly to children aged 5 to 17 months living in a malaria-endemic area: **Amended (04 July 2006)**
  - to assess safety until ten months post Dose 1.

Refer to Section 10.1 for definition of the primary endpoint.

2.2. Secondary objectives

- For RTS,S/AS01E (on a 0, 1-month and a 0, 1, 2-month and a 0, 1, 7-month schedule) and RTS,S/AS02D (on a 0, 1-month, ***0, 1, 2-month*** and a 0, 1, 7-month schedule) when administered intramuscularly to children aged 5 to 17 months living in a malaria-endemic area: **Amended (04 July 2006)**
  - to assess safety and reactogenicity until 30 days post Dose 1
  - to describe the evolution of antibody responses to the circumsporozoite (CS) antigen until ten months post Dose 1
  - to assess the antibody responses to the hepatitis B surface (HBs) antigen at one month post final vaccine dose

Refer to Section 10.2 for definitions of secondary endpoints.

2.3. Tertiary objectives

- For RTS,S/AS01E (on a 0, 1-month and a 0, 1, 2-month and a 0, 1, 7-month schedule) and RTS,S/AS02D (on a 0, 1-month, ***0, 1, 2-month*** and a 0, 1, 7-month schedule) when administered intramuscularly to children aged 5 to 17 months living in a malaria-endemic area: **Amended (04 July 2006)**
  - to assess safety between ten months post Dose 1 up to 19 months post Dose 1
  - to assess antibody responses to the circumsporozoite (CS) antigen at 19 months post Dose 1
  - to assess antibody responses to the hepatitis B surface (HBs) antigen at 19 months post Dose 1.

Refer to Section 10.3 for definitions of tertiary endpoints.

2.4. Exploratory Objectives

- For RTS,S/AS01E (on a 0, 1-month and a 0, 1, 2-month and a 0, 1, 7-month schedule) and RTS,S/AS02D (on a 0, 1-month, ***0, 1, 2-month*** and a 0, 1, 7-month schedule) when administered intramuscularly to children aged 5 to 17 months living in a malaria-endemic area: **Amended (04 July 2006)**
  - to assess safety up to 19 months post Dose 1 when vaccinees are stratified by age at the time of Dose 1
  - to assess antibody responses to the CS antigen and the HBs antigen up to 19 months post Dose 1 when vaccinees are stratified by age at the time of Dose 1.
  - to evaluate T-cell-mediated immune response (CMI) to CS antigen up to 19 months post Dose 1.

Refer to Section 10.4 for definitions of exploratory endpoints.

3. STUDY DESIGN OVERVIEW

Figure 1 Study Design Overview: children to receive RTS,S/AS01E or RTS,S/AS02D on a 0, 1-month schedule

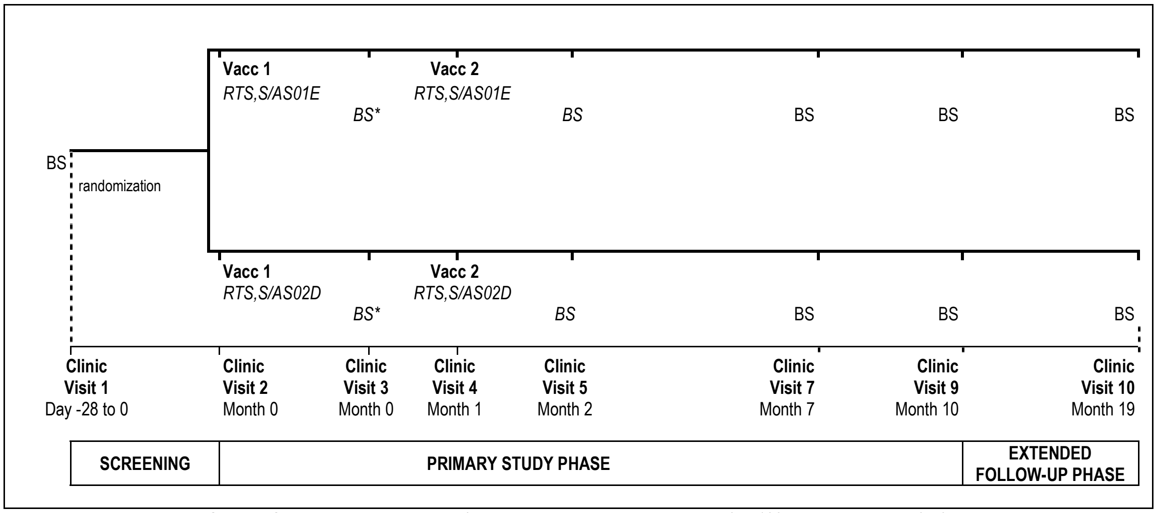

KEY: BS; Blood Sample. **Vacc;** Vaccination. * this blood sample only carried out on first 100 children to present for follow-up

Figure 2 Study Design Overview: children to receive RTS,S/AS01E or Rabies vaccine *or RTS,S/AS02D* on a 0, 1, 2-month schedule

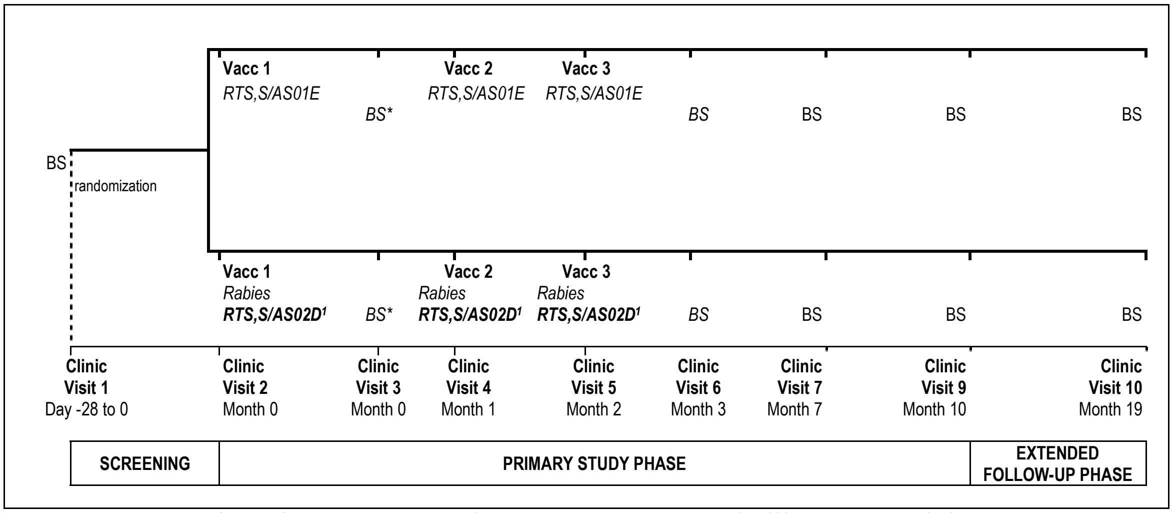

KEY: BS; Blood Sample. **Vacc;** Vaccination. * this blood sample only carried out on first 100 children to present for follow-up

1. The Rabies vaccine will be used only at Kintampo-KHRC; The RTS,S/AS02D vaccine will be used at Kumasi-KCCR/SMS.

Amended (04 July 2006)

Figure 3 Study Design Overview: children to receive RTS,S/AS01E or RTS,S/AS02D on a 0, 1, 7-month schedule

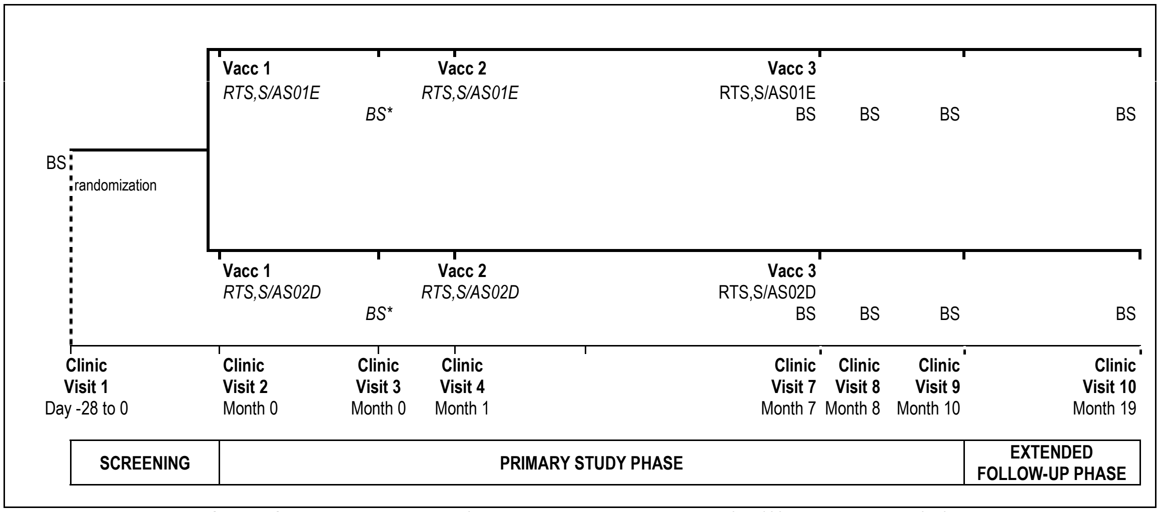

KEY: BS; Blood Sample. **Vacc;** Vaccination. * this blood sample only carried out on first 100 children to present for follow-up

- Experimental design: Phase II, controlled, partially blind (blind to vaccine administration, open to vaccination schedule), randomized (1: 1: 1: 1: 1: 1 ratio) trial with six treatment groups ***at each study site***.

Vaccine	Schedule (Months)	Number of children to be enrolled	Estimated number evaluable
RTS,S/AS01E (0.5 mL dose)	0, 1, 2	90	75
Rabies vaccine (0.5 mL dose)[a]	0, 1, 2	~~90~~ ***45***	~~75~~ ***40***
RTS,S/AS02D (0.5 mL dose)[b]	***0, 1, 2***	***45***	***40***
RTS,S/AS02D (0.5 mL dose)	0, 1, 7	90	75
RTS,S/AS01E (0.5 mL dose)	0, 1, 7	90	75
RTS,S/AS02D (0.5 mL dose)	0, 1	90	75
RTS,S/AS01E (0.5 mL dose)	0, 1	90	75

[a] The Rabies vaccine will only be used at Kintampo – KHRC
[b] Enrollment will occur at Kumasi – KCCR/SMS

Amended (04 July 2006)

- Healthy male and female children aged 5 to 17 months will be screened; those determined to be eligible, based on the inclusion and exclusion criteria, will be enrolled in the study.
- Only those children whose parents/guardians provide written proof that the child has completed a 3 dose regimen of a licensed Hepatitis B vaccine in infancy will be included in this trial of an experimental Hepatitis B vaccine.
- Route of administration: all vaccines will be administered by the intramuscular route to the left deltoid.

- Each child will be observed for at least 60 minutes after vaccination to evaluate and treat any acute adverse events (AEs).
- There will be a 7-day follow-up period for solicited AEs post-vaccination: Day 0 evaluation will be carried out by the study physician at the study center. Subsequently, trained field workers will visit the children to solicit AEs on Days 1 to 6 after each vaccination (evaluation on Day 6 post Dose 1 will be carried out by the study physician at the study center).
- There will be a 30-day (day of vaccination and 29 subsequent days) follow-up after each vaccine dose for reporting unsolicited symptoms.
- Serious adverse events (SAEs) will be recorded throughout the study period. Prior to vaccination, any SAEs due directly to study procedures will be captured. All SAEs will be captured, beginning with the administration of the first dose and ending 19 months after Dose 1 of study vaccines. After Month 10 (the primary study phase) of the trial, all enrolled children will be visited at home monthly by field workers until study conclusion to ensure complete identification of all SAEs.
- Unblinded safety reports will be reviewed by the DSMB at 3 timepoints: these will summarize the experience of the first 90 children to complete 7 days of follow-up post Dose 1 who are enrolled to be vaccinated on a 0, 1, 2-month schedule. The reports will be made available to the DSMB post Dose 1, Dose 2 and Dose 3. Data will e reviewed between doses to authorize progression to the next sequential dose.
- Blood samples for assessment of hepatic and renal function will be collected at all timepoints at which blood samples for immunogenicity are taken (see below). In addition, blood will be taken from the first 100 subjects who present for follow-up at Day 6 post Dose 1 for inclusion in the first DSMB safety review.
- Anti-CS antibody titers, anti-HBs antibody titers and CMI response will be determined at the time points indicated in the table below.

	**PRIMARY STUDY PHASE**												**EXTENDED FOLLOW-UP PHASE**								
Study Month	Scr	0	1	2	3	4	5	6	7	8	9	10	11	12	13	14	15	16	17	18	19
0, 1-month vaccination schedule																					
CS/HBs	**HBs**			**HBs**																	**HBs**
	**CS**			**CS**					**CS**			**CS**									**CS**
CMI				**CMI**																	**CMI**
VACC		**X**	**X**																		
0, 1, 2-month vaccination schedule																					
CS/HBs	**HBs**				**HBs**																**HBs**
	**CS**				**CS**				**CS**			**CS**									**CS**
CMI					**CMI**																**CMI**
VACC	~~**X**~~	**X**	**X**	**X**																	
0, 1, 7-month vaccination schedule																					
CS/HBs	**HBs**									**HBs**											**HBs**
	**CS**								**CS**	**CS**		**CS**									**CS**
CMI										**CMI**											**CMI**
VACC	~~**X**~~	**X**	**X**						**X**												

Scr: Screening VACC: Vaccination

Amended (04 July 2006)

- This study will not capture cases of malaria for the analysis of efficacy. All cases of malaria presenting during the unsolicited period will be captured as unsolicited events. Cases at anytime during the study that meet the criteria for a SAE will be reported as SAEs.
- The access of the study population to Insecticide Treated Bednets, HIV voluntary counseling and testing and HIV antiretroviral therapy according to national recommendations will be facilitated, in collaboration with the government services.
- The organization of the dates of experimental vaccination will take into account the EPI vaccination with measles and yellow fever vaccination at nine months of age and allow a minimum of 14 days period of separation between the study vaccines and the Measles and Yellow Fever vaccines. Any EPI vaccines administered during the duration of the study will be documented in the CRF.
- Final analysis will be carried out on data collected up to ten months post Dose 1. An addendum safety analysis will be performed on data collected up to the end of the study, 19 months post Dose 1.
- Data collection: conventional Case Report Form (CRF).
- 540 subjects will be enrolled. It is expected that approximately ~~450~~ ***455*** subjects will be evaluable at study end. **Amended (04 July 2006)**

4. STUDY COHORT

4.1. Kintampo Health Research Centre and Kumasi Centre for Collaborative Research/School of Medical Sciences

This study will be conducted by two investigative teams in Ghana; one team from Kintampo Health Research Centre (KHRC) (refer to Section 4.1.2) and one from Kumasi Centre for Collaborative Research/School of Medical Sciences (KCCR/SMS) in Kumasi (refer to Section 4.1.1). Each investigative team will recruit half of the subjects for the trial.

Komfo Anokye Teaching Hospital (KATH), located in the city of Kumasi will provide all tertiary level care for this study (refer to Section 4.1.3).

Malaria is the most important cause of morbidity in Ghana, accounting for about 45% of all patients' hospital attendance. In 2001, malaria was the cause of 22% of deaths of children under the age of 5 years [Afrane, 2004]. *P. falciparum* is the predominant malaria species. *P. malariae* is found in 1 to 2% of infections. *Anopheles gambiae* sensu stricto is the main vector of malaria in the area in which the study will take place. *An. funestus* is also encountered; while it does not appear to be an important malaria vector in the Kumasi area [Afrane, 2004], it is thought to be becoming increasingly important in the Kintampo area (Owusu-Agyei, personal communication, January 2006).

The first line of treatment recommended by the Ministry of Health is artesunate/amodiaquine for uncomplicated malaria and quinine for complicated malaria. Parasite resistance to chloroquine and sulfadoxine/pyrimethamine is widespread in Ghana. At least 35% of infections are resistant to chloroquine,18% resistant to sulfadoxine/pyrimethamine.

4.1.1. Kumasi Centre for Collaborative Research/School of Medical Sciences (KCCR/SMS)

The Kumasi Centre for Collaborative Research (KCCR) and the School of Medical Sciences (SMS) are situated on the campus of the Kwame Nkrumah University of Science and Technology (KNUST) in Kumasi. KCCR was created jointly by the Ministry of Health of the Republic of Ghana, the Kwame Nkrumah University of Science and Technology (KNUST), Kumasi, Ghana, and the Bernhard-Nocht Institute for Tropical Medicine (BNI), Hamburg, Germany, with the signing of an agreement in 1997 between the Republic of Ghana and the City/State of Hamburg. KCCR facilities were opened in October 2003. The philosophy behind KCCR is to facilitate and coordinate collaborative research projects between Ghanaian and both Northern and Southern partners. The School of Medical Sciences (SMS) at KNUST is one of the principal Ghanaian partners working with the KCCR.

The running costs of KCCR are met by externally funded projects and some core funding is at present provided by the BNI.

SMS-KNUST was established in May 1975 as Ghana's second medical school to train doctors, scientists and laboratory technologists.

Laboratories and offices of 400 m^2 at KCCR are equipped with telephone, fax, internet connection (through a satellite connection via KNUST) and generators. The laboratory complex consists of; a molecular biology unit, an immunology unit, a parasitology unit and a virology unit.

The unit has three generators including one mobile generator that can be used at field station sites.

4.1.1.1. KCCR/SMS: Study Area Geography, Population & Malaria Epidemiology

The study will be coordinated from KCCR/SMS but the field site will be in the town of Agogo in Asante Akim North District, 70 km from Kumasi. The district population is 126 477 (2000 population and housing census), with approximately 15% of the population aged less than 5 years. Agogo, a town of population 28 271, is set in the forest region where the main occupation is farming. The majority of the population is Akan with a small group of migrants from the north of the country. The main language used is Twi.

There is approximately 1400 mm of rain per year, with the main rainfall occurring between April to July and again in September and October [Afrane 2004]. The entomological inoculation rate is estimated to be 70 infective bites per person per year. It is estimated that approximately 10% of people in Agogo sleep under an ITN.

4.1.1.2. Agogo Presbyterian Hospital

The Presbyterian Hospital in Agogo was founded in 1931. Salaries are paid by the Ghanaian Ministry of Health. Currently 10 doctors are on staff, including 2 general surgeons/obstetricians, 1 obstetrician/gynecologist and a pediatrician. In addition there are approximately 90 nurses. In 2003 approximately 38% of admissions to the pediatric ward were diagnosed with malaria, more than a quarter of these with severe malaria (severe anemia, deep breathing/prostration or cerebral malaria). A daily clinic is in operation for children under the age of 5 years. Between 520 and 870 cases are seen per month. Hematology and biochemistry testing will be in place for the study.

4.1.2. Kintampo Health Research Centre (KHRC)

KHRC, based in the town of Kintampo in the Brong-Ahafo Region, was founded in 1994. It is an institution of the Ministry of Health of Ghana with autonomy of administrative and financial management. The center focuses its research on a range of locally important health issues including malaria, prevention and treatment of anemia, and management of childhood illness amongst others. The centre works in close collaboration with Kintampo District Hospital (refer to 4.1.2.2), located on a common campus. Research laboratories are located within KHRC while the hospital also has its own laboratory for routine work.

The core KHRC research group consists of three epidemiologists, three public health physicians, five research fellows, 25 assistant research officers, two laboratory technologists, one laboratory technician and seven data managers.

KHRC receives most of its funding through individual projects supported by international agencies including the Department of International Development, UK, United States Agency for International Development, Gates Malaria Partnership, Wellcome Trust, UK and the WHO. The Government of Ghana through the Ministry of Health supports some routine activities.

4.1.2.1. KHRC: Study Area Geography, Population & Malaria Epidemiology

Kintampo is situated 200 km north of Kumasi within the forest-savanna transition. The average rainfall in Kintampo is about 1250 mm of rain a year, falling mainly between March and June.

The local population is ethnically diverse. The main groups comprise people from the Bono and Mo tribes. There is a large population from northern Ghana that have permanently migrated into Kintampo District. Most of the population in the study area speak Twi. A large proportion of the population is illiterate. Most settlements are located along the main road leading north out of Kintampo town.

There are approximately 30 births per 1000 population. The infant mortality rate is about 60 per 1000 live births.

The entomological inoculation rate determined in 2004 in Kintampo District was 269 infective bites per person per year. Children suffer about 8 episodes of clinical malaria per year. Anemia is common; 50% of children have hemoglobin levels less than < 11g/dL throughout the year. Lower hemoglobin levels are more common in November/December (during the rainy season) when up to 12.6% of children have been recorded with hemoglobin < 8g/dL. Insecticide treated bednets (ITNs) are available throughout Ghana for subsidized purchase and on the open market. It is estimated that approximately 10% of people in Kintampo sleep under an ITN.

4.1.2.2. Kintampo District Hospital (KDH)

A total of 80 beds are available, 30 dedicated to pediatric patients. Currently the hospital is staffed full-time by three doctors, two medical assistants, one pharmacist, three dispensing technicians and 33 nurses who run a 24 hour call. During the course of the trial, these will be supplemented by doctors from KHRC.

Facilities for basic hematology, biochemistry, microscopy, x-ray and ultrasound are available at the KDH. A back-up generator is in place.

Children requiring more intensive diagnostics or specialist care will be referred to the KATH in Kumasi (refer to Section 4.1.3).

4.1.3. Komfo Anokye Teaching Hospital (KATH), Kumasi

All tertiary level care for this study will be provided by KATH. The department of child health at KATH admits approximately 6000 children per year. It consists of a mother and baby unit, pediatric emergency unit, three general pediatric wards and a pediatric surgery unit. The department is staffed by nine pediatricians and 15 residents, many of whom have experience in conducting clinical trials within the department of child health.

The laboratory has a cell counter, an analyzer for glucose/lactate, an analyzer for clinical chemistry, a -80°C freezer, two 4°C refrigerators, a centrifuge and a 37°C incubator. The laboratory is staffed 24 hours a day.

4.2. HIV Services

The prevalence of HIV in Ghana is estimated to be approximately 3% [UNAIDS/WHO, 2004]. HIV counseling and testing are available both at KDH and at Agogo hospital. All children confirmed to be infected with HIV will be referred to the HIV clinic at KATH. KATH has both an adult and a pediatric HIV clinic providing access to immunological testing (CD4 counts) and anti retroviral therapies.

4.2.1. Treatment of HIV-infected children

As part of the national antiretroviral program, three categories of antiretrovirals are provided to pediatric patients infected with HIV at KATH; 1) Nucleoside Reverse Transcriptase Inhibitors (NRTIs); 2) non-NRTIs; 3) Protease Inhibitors. Currently the cost of therapy is subsidized to the value of 50 000 Ghanaian Cedi per person per month (roughly equivalent to €4.66 [exchange rate determined December 2005]). Treatment at KATH is given according to Ghanaian Ministry of Health policy.

4.3. Inclusion criteria

All subjects must satisfy the following criteria at study entry:

- A male or female child between 5 months and 17 months of age at the time of first vaccination.
- Written or oral, signed or thumb-printed and witnessed informed consent obtained from the parent(s)/guardian(s) of the child.
- Subjects who the investigator believes that their parents/guardians can and will comply with the requirements of the protocol (e.g. return for follow-up visits) should be enrolled in the study.
- Proof that child has received a full 3-dose regimen of licensed Hepatitis B vaccine in infancy.

4.4. Exclusion criteria for enrolment

The following criteria should be checked at the time of study entry. If any apply, the subject must not be included in the study:

- Acute disease at the time of enrolment (acute disease is defined as the presence of a moderate or severe illness with or without fever). All vaccines can be administered to persons with a minor illness, such as diarrhea or mild upper respiratory infection without fever, i.e. axillary temperature < 37.5°C.
- Serious acute or chronic illness determined by clinical or physical examination and laboratory screening tests including, but not limited to:
  - Any confirmed or suspected immunosuppressive or immunodeficient condition, based on medical history and physical examination (no laboratory testing required)
  - A family history of congenital or hereditary immunodeficiency
  - History of splenectomy
  - Major congenital defects
  - Tuberculosis
  - History of any neurologic disorders or seizures
  - Malnutrition at screening defined as weight for age Z-score less than -3 or other clinical signs of malnutrition.
- Laboratory screening tests out of acceptable limits:
  - refer to Table 18.
- Planned administration/administration of a vaccine not foreseen by the study protocol within 30 days of the first dose of vaccine(s) with the exception of Tetanus Toxoid or scheduled Yellow Fever or Measles vaccine.
- Use of any investigational or non-registered drug or vaccine within 30 days preceding the first dose of study vaccine, or planned use during the study period.
- Administration of immunoglobulins, blood transfusions or other blood products within the three months preceding the first dose of study vaccine or planned administration during the study period.
- Chronic administration (defined as more than 14 days) of immunosuppressants or other immune-modifying drugs within six months prior to the first vaccine dose (for corticosteroids, this will mean prednisone, or equivalent, ≥ 0.5 mg/kg/day. Inhaled and topical steroids are allowed).
- Previous participation in any other malaria vaccine trial.
- Simultaneous participation in any other clinical trial.
- Any twins

- History of allergic reactions (significant IgE-mediated events) or anaphylaxis to previous immunizations.
- History of allergic disease or reactions likely to be exacerbated by any component of the vaccine.
- Any other findings that the investigator feels would increase the risk of having an adverse outcome from participation in the trial.

4.5. Elimination criteria during the study

The following criteria should be checked at each visit subsequent to the first visit. If any become applicable during the study, it will not require withdrawal of the subject from the study but may determine a subject's evaluability in the according-to-protocol (ATP) analysis.

- Administration of a vaccine (except Tetanus Toxoid, or scheduled Yellow Fever or Measles vaccine) not foreseen by the study protocol during the period starting from 30 days before Dose 1 and ending 30 days after Dose 3.
- Use of any investigational or non-registered product (drug or vaccine) other than the study vaccines during the study period.
- Administration of immunoglobulins and/or any blood products during the study period.
- Chronic administration (defined as more than 14 days) of immunosuppressants or other immune-modifying drugs during the study period (for corticosteroids, this will mean prednisone, or equivalent, ≥ 0.5 mg/kg/day. Inhaled and topical steroids are allowed).
- Failure to thrive.

4.6. Contraindications to subsequent vaccination

4.6.1. Indications for deferral of vaccination

The following events constitute contraindications to administration of RTS,S/AS02D, RTS,S/AS01E or Rabies vaccine at that point in time; if any one of these AEs occur at the time scheduled for vaccination, the subject may be vaccinated at a later date, within the time window specified in the protocol, or withdrawn at the discretion of the investigator. AEs should be followed-up according to the instructions in Section 8.6:

- Acute disease at the time of administration of investigational product (acute disease is defined as the presence of a moderate or severe illness with or without fever). All vaccines can be administered to persons with a minor illness such as diarrhea or mild upper respiratory infection without fever, i.e. axillary temperature < 37.5°C.
- Axillary temperature of ≥ 37.5°C.
- Yellow Fever or Measles vaccination within 14 days of any trial vaccination (i.e. RTS,S/AS02D, RTS,S/AS01E or Rabies vaccine).

4.6.2. Absolute contraindications to further vaccination

The following AEs constitute absolute contraindications to further administration of RTS,S/AS02D, RTS,S/AS01E or Rabies vaccine; if any of these AEs occur during the study, the subject must not receive additional doses of vaccine, but may continue other study procedures at the discretion of the investigator. AEs should be followed-up according to the instructions in Section 8.6:

- Acute allergic reaction (significant IgE-mediated events) or anaphylaxis following the administration of vaccine investigational product.
- Any confirmed or suspected immunosuppressive or immunodeficient condition, including human immunodeficiency virus (HIV) infection.

5. CONDUCT OF STUDY

5.1. Ethics and regulatory considerations

The study will be conducted according to Good Clinical Practice (GCP), the Declaration of Helsinki (Protocol Appendix A), and local rules and regulations of Ghana.

Submission of the protocol and any protocol amendments to regulatory agencies will occur in accordance with Ghanaian regulatory requirements. The timing of the submission relative to IEC/IRB submission or approval and whether or not the authority will provide their approval of or favorable opinion on the protocol or amendment before it can be implemented will depend on local regulatory requirements.

5.1.1. Institutional Review Board/Independent Ethics Committee (IRB/IEC)

Each IRB/IEC will be constituted according to the local laws/customs of each participating country. The ICH Harmonized Tripartite Guideline for Good Clinical Practice recommends that IRBs/IECs should include:

a. At least five members.

b. At least one member whose primary area of interest is in a non-scientific area.

c. At least one member who is independent of the institution/ study site.

Only those IRB/IEC members who are independent of the investigator and the sponsor of the study will vote/ provide opinion on a study-related matter.

A list of the professions of the IRBs'/IECs' members will be obtained by the Principal Investigators (PIs) or their delegates.

This protocol and any other documents that the IRBs/IECs may need to fulfill their responsibilities, including subject recruitment procedures and information about payments and compensation available to subjects, will be submitted to each IRB/IEC by the PIs or their delegates. Written and dated unconditional approval/favorable opinion

from each IRB/IEC of the protocol and amendment (if any and applicable), written informed consent form ICF), consent form updates (if any), subject recruitment procedure(s) (e.g. advertisements), and any other written information to be provided to subjects will be in the possession of the investigator and GSK before commencement of the study. This approval/favorable opinion will refer to the study by study title and number with exact protocol version and date, and will identify the documents reviewed and state the date of review. Relevant GSK Biologicals' data will be supplied by the GSK Biologicals' Central Study Coordinator to the independent IRBs/IECs for review and approval of the protocol. Verification of IRBs/IECs unconditional approval of the protocol and the written informed consent statement will be transmitted by the PIs to the GSK Biologicals' Central Study Coordinator, using the standard notification form, prior to shipment of vaccine supplies and CRFs to the site.

No deviations from, or changes to, the protocol will be initiated without prior written sponsor and IRBs'/IECs' approval/favorable opinion of an appropriate amendment, except when necessary to eliminate immediate hazards to the subjects or where permitted by all applicable regulatory requirements or when the change(s) involves only logistical or administrative aspects of the study (e.g., change of monitor[s], telephone number[s].) Administrative changes and amendments not submitted for approval will be submitted to the IRB/IEC for information only. However, written verification that such documents were submitted will be obtained. Approvals/ verifications will be transmitted in writing by the PIs.

The IRB/IEC will be informed by the Principal Investigators of:

- all subsequent protocol amendments, informed consent changes or revisions of other documents originally submitted for review,
- serious and/or unexpected adverse events occurring during the study,
- all subsequent protocol administrative changes (for information),
- new information that may affect adversely the safety of the subjects or the conduct of the study,
- regular updates and/or request for re-approval,
- when the study has been completed.

If the trial is prematurely terminated or suspended for reasons including, but not limited to, safety or ethical issues or severe non-compliance, the sponsor will promptly inform the regulatory authorities of the termination or suspension and the reason(s) for the termination or suspension. If required by applicable regulations, the PIs will inform the IEC/IRB promptly and provide the reason for the suspension or termination (see Appendix B for further details).

5.1.2. Informed consent

The details of the informed consent process are provided in Appendix C. The following principles will also apply.

In obtaining and documenting informed consent, the investigators should comply with the applicable regulatory requirement(s), and should adhere to GCP and to the ethical principles that have their origin in the appended Declaration of Helsinki. Prior to the beginning of the trial, the investigators should have the IRB/IEC's written approval/favorable opinion of the written informed consent form and any other written information to be provided to the subjects' parents/guardians.

Information should be given in both oral and written form whenever possible and as deemed appropriate by the IRB/IEC.

An investigator or designate will describe the protocol to potential subjects' parents/guardians in person. The Subject Information and Consent Form may be read to the subjects' parents/guardians, but, in any event, the investigator or designate shall give the subjects' parents/guardians ample opportunity to inquire about details of the study and ask any questions before dating and signing the Consent Form.

Subject Information and Informed Consent Forms must be in a language fully comprehensible to the prospective subjects' parents/guardians. Informed consent shall be documented by the use of a written consent form approved by the IRB/IEC and signed and dated by the subjects' parents/guardians and by the person who conducted the informed consent discussion. The signature confirms the consent is based on information that has been understood. All individuals will have the study, the Subject Information and Consent Form explained to them point by point by the interviewer in the presence of an impartial witness. The subjects' parents/guardians will thumbprint or sign the consent form. The witness will also sign and date the consent form.

Each subject's signed Informed Consent Form must be kept on file by the investigator for possible inspection by Regulatory Authorities and/or GSK Biologicals' professional and Regulatory Compliance persons. The subjects should receive a copy of the signed and dated written Informed Consent Form and any other written information provided to the subjects and should receive copies of any signed and dated consent form updates. Any amendments to the written information will be provided to subjects.

Both the informed consent discussion and the written Informed Consent Form and any other written information to be provided to the subjects' parents/guardians should include explanations of the following:

a. That the trial involves research.

b. The purpose of the trial.

c. The trial treatment(s) and the probability for random assignment to each treatment.

d. The trial procedures to be followed, including all invasive procedures.

e. The subject's and/or subject's parents'/guardians' responsibilities.

f. Those aspects of the trial that are experimental.

g. The reasonably foreseeable risks or inconveniences to the subjects and, when applicable, to an embryo, fetus or nursing infant.

h. The reasonable expected benefits. When there is no intended clinical benefit to subjects, the subjects and/or subjects' parents/guardians should be made aware of this.

i. The alternative procedure(s) or course(s) of treatment/ methods of prevention that may be available to subjects, and their important potential benefits and risks.

j. The compensation and/or treatment available to subjects in the event of trial-related injury.

k. The anticipated prorated payment, if any, to subjects / subjects' parents/guardians for participating in the trial.

l. The anticipated expenses, if any, to subjects / subjects' parents/guardians for participating in the trial.

m. That the subjects' participation in the trial is voluntary and subjects / subjects' parents/guardians may refuse to participate or withdraw from the trial, at any time, without penalty or loss of benefits to which subjects are otherwise entitled.

n. That the monitor(s), the auditor(s), the IRB/IEC and the regulatory authority(ies) will be granted direct access to the subject's original medical records for verification of clinical trial procedures and/or data, without violating the confidentiality of subjects, to the extent permitted by the applicable laws and regulations and that, by signing a written informed consent, the subject / the subject's parents/guardians is authorizing such access.

o. That records identifying subjects will be kept confidential and, to the extent permitted by the applicable laws and/or regulations, will not be made publicly available. If the results of the trial are published, subjects' identity will remain confidential.

p. That the subjects / subjects' parents/guardians will be informed in a timely manner if information becomes available that may be relevant to the subjects' / the subjects' parents/guardians willingness for continued participation in the trial.

q. The person(s) to contact for further information regarding the trial and the rights of trial subjects, and who to contact in the event of trial-related injury.

r. The foreseeable circumstances and/or reasons under which a subject's participation in the trial may be terminated.

s. The expected duration of a subject's participation in the trial.

t. The approximate number of subjects involved in the trial.

GSK Biologicals will prepare a model Informed Consent Form which will embody all the elements described above. While it is strongly recommended that this model document be followed as closely as possible, the informed consent requirements given in this document are not intended to pre-empt any local regulations which require additional information to be disclosed for informed consent to be legally effective. Clinical judgment, local regulations and requirements should guide the final structure and content of the document.

The investigator has the final responsibility for the final presentation of Informed Consent Form, respecting the mandatory requirements of local regulations. The consent form generated by the investigator with the assistance of the sponsor's representative, must be approved (along with the protocol, and any other necessary documentation) by the IRB/IEC and be acceptable to GSK Biologicals.

5.1.3. Safety monitoring plan

This trial is overseen by a Data Safety Monitoring Board (DSMB) operating under a charter assisted by a Local Safety Monitor (LSM) at each site.

The DSMB will be notified of all SAEs within 24 hours. In addition there are defined points during the trial at which cumulative safety data will be reviewed by the DSMB. There will be 3 reports as defined in Section 5.1.3.3.

The PIs, LSM and DSMB are empowered to suspend the trial for any safety concern. To supplement this, the protocol defines criteria for the suspension of vaccination (refer to Section 5.1.3.4).

5.1.3.1. Data Safety Monitoring Board (DSMB)

An independent committee consisting of experts in malaria, pediatrics, statistics and other appropriate disciplines has been appointed to oversee ethical and safety aspects of the study conduct. A quorum of 3 members is required at scheduled meetings.

The role of the DSMB includes the review of the implementation and progress of the study. It provides initial, regular, and closing advice on safety-related issues to GSK Biologicals. Its advice is based on the interpretation of study data with reference to the study protocol.

The DSMB will confer before the initiation of the study (pre-initiation review), during the study at points of safety review and at the close of the study. They will review the Protocol and Report and Analysis Plan (RAP). Other unscheduled meetings may be required. Meetings must be documented and minutes made available to the sponsors. The DSMB may, if deemed necessary, convene a meeting with, or request further information from the Principal Investigators, the Medical Monitor/Local Safety Monitors and GSK Biologicals' and MVI at PATH's designated project representatives at any stage of the study.

The DSMB is empowered to suspend the enrollment to the trial and/or vaccination on the trial pending review of potential safety issues; complete details of this process are given in Section 5.1.3.4.

5.1.3.1.1. Data Reviewed by the DSMB

The DSMB must be informed by the Local Safety Monitors (LSM) of the following safety data on an 'as received' basis:

- All SAEs;
- All withdrawals of study subjects by the Principal Investigator or the parent(s)/guardian(s) of a subject due to adverse events.

The DSMB will receive from the sponsor, GSK Biologicals:

- Safety summary reports at 3 predefined timepoints as defined in Section 5.1.3.3.
- New information that may affect adversely the safety of the subjects or the conduct of the study;
- All subsequent protocol amendments, informed consent changes or revisions of other documents originally submitted for review;
- All subsequent protocol modifications (for information).

5.1.3.2. Local Safety Monitor (LSM)

The overall role of the Local Safety Monitors (LSM), who are experienced clinicians based in-country, will be to support the clinical investigators and to act as a link between the investigators and the DSMB.

The LSM's role will include:

- Acting as the study volunteer's advocate;
- Promptly communicating relevant safety information to the DSMB;
- Providing advice to the investigators on whether a set of clinical circumstances in a study warrants formal notification to the DSMB;
- Unblinding a subject if deemed necessary to allow for adequate treatment;
- Liaising closely with the chair of the DSMB throughout the course of the trial;
- Suspension of vaccination for a major safety concern pending discussion with the DSMB (see Section 5.1.3.4 for full details).

5.1.3.2.1. Data Reviewed by the LSM

The relevant LSM must be informed by the investigator on an 'as received' basis of:

- All SAEs;
- All withdrawals of study subjects by the Principal Investigator or the parent(s)/ guardian(s) of a subject due to adverse events.

5.1.3.3. Safety monitoring reports

Three unblinded safety reports will be produced for the DSMB on safety information collected on vaccinees. The first safety report will be produced on data collected on the first 90 vaccinees on a 0, 1, 2-schedule to complete 7 days of follow-up post Dose 1. A second and third DSMB report will be produced on data from these children up to the time that they complete 7 days of follow-up post Dose 2 and post Dose 3 respectively.

The first two reports will be reviewed prior to authorization by the DSMB to allow progress to the next sequential dose. The third report will be used by the DSMB to authorize age de-escalation in other trials of RTS,S/AS01E.

An independent statistician will analyze the data and prepare an unblinded report for the DSMB thereby maintaining the blind of the malaria project team at GSK Biologicals and the investigator group. Data will be reviewed by the DSMB before progression to the next sequential vaccine dose may be authorized.

The reports will contain the following data on 90 vaccinees on a 0, 1, 2-month schedule:

- All SAEs and any relationship to vaccines to date
- All solicited AEs tabulated by severity grading (any and Grade 3 alone) and relationship to vaccine.
  - Should the investigator judge a case of Grade 3 fever to be unrelated to vaccination, an alternative explanation for the cause of the fever will be provided
- All unsolicited AEs tabulated by severity grading (any and Grade 3 alone) and relationship to vaccine.
- All withdrawals of study subjects by the Principal Investigator due to adverse events recorded from the children or withdrawals of children by the parent(s)/guardian(s) (expressed as percentage of subjects enrolled).

In addition, the report post Dose 1 will include all laboratory values collected from the first 100 children to present for Clinic Visit 2 presented as number of subjects out of range (above and below normal range) tabulated by toxicity grading scale

- For all subjects with toxicity grading scale ≥ 3, full clinical details will be provided (refer to Table 18 for toxicity grading scales).

If a criterion for the suspension of progression to the next sequential vaccine dose is met, or the DSMB have any safety concerns about the vaccines, they may suspend the ongoing enrollment and vaccination in the trial. The process outlined in Section 5.1.3.4 will be followed.

5.1.3.4. Process for the suspension of progression to the next sequential vaccine dose

Suspension of the next sequential vaccine dose, pending full review of all available data by the DSMB will take place if:

- The Principal Investigator suspends vaccination for any of the following SAEs pending review by the DSMB;
  - Death or life-threatening SAE which is judged to be related to the study vaccine;
  - Anaphylactic shock reaction in an enrolled subject following vaccination.

- The DSMB recommend suspension of all vaccination for any one SAE or pattern of SAEs. The DSMB will communicate their recommendation to the Principal Investigator who will enact it. The DSMB will notify the sponsors of their decision immediately;
- A safety report shows > 5% of subjects vaccinated with RTS,S/AS01E are withdrawn by the investigator for local or systemic reactogenicity.
  - In making their recommendation the DSMB will take into account the full clinical history of each withdrawn child.
- A safety report shows > 5% of doses followed by fever > 39.0°C judged to be related to vaccination in recipients of RTS,S/AS01E.
  - In making their recommendation the DSMB will review the investigators assessment of relatedness of all Grade 3 fevers.
- The DSMB recommend suspension of enrollment following their review of a safety summary reports.

5.1.3.5. Process if the trial is suspended

Although the trial may be suspended by the DSMB, the LSMs or the Principal Investigator, it is the responsibility of the sponsor (GSK Biologicals) to make the recommendation whether or not the trial should be stopped permanently.

If the trial is suspended, the DSMB will review all available information (which will include the experience of all children to have been vaccinated) and make a recommendation to the study sponsor (GSK Biologicals) whether to recommence the trial and proceed to the next sequential vaccine dose, or to stop the trial permanently. In the event that the DSMB recommend to stop the trial permanently, the FDA will be informed by GSK Biologicals that the trial is suspended.

In the event that the trial is suspended on the recommendation of the DSMB the sponsor (GSK Biologicals) will evaluate the information. If the sponsor concurs with the DSMB's recommendation to suspend the trial, GSK Biologicals will inform the FDA that the trial has been stopped permanently. If the sponsor's recommendation is to continue, then a report will be submitted to the FDA detailing the rationale used in reaching this decision. The agreement of the FDA will be obtained prior to restarting the trial.

5.1.4. Exposure to rabies

The parents/guardians of all subjects enrolled will be reminded at each Clinic Visit that should their child be bitten or scratched by a dog or cat, they must immediately consult a physician for treatment.

5.1.5. Cross over immunization with Rabies vaccine

After completion of the study, Rabies immunization on a 0, 1, 28-day schedule will be offered for all children ***enrolled at the KHRC and*** that did not receive it during the study

(i.e. those subjects that received RTS,S/AS02D or RTS,S/AS01E). **Amended (04 July 2006)**

5.2. Storage of study documentation at investigator's sites

All study documentation containing personal information relating to study subjects will be kept in a secure locked area at the investigator's sites. Such documentation will only be made available to authorized personnel. All electronic data kept at the investigator's site are kept secure. Computer access is only available to authorized personnel.

5.3. Recruitment/Screening

5.3.1. Community information

The communities in which the study will take place will be informed about the nature and design of the study. Refer to Appendix C for an overview of the recruitment plan of the study.

5.3.2. Screening of volunteers

Only children with a written Informed Consent Form, signed/thumbprinted and dated by parents/guardians will be screened.

Comprehension of the information contained within the Informed Consent form will be checked prior to screening by an oral interview with the volunteer's parent(s)/guardian(s), according to SOPs at KHRC and KCCR/SMS. If consent is not available from both parents or guardians, the reason for the unavailability of one of the parents or guardians will be specified on the consent form.

Subject numbers will be allocated to all volunteers who are consented for screening by their parent(s)/guardian(s). Subject numbers will be issued consecutively ***at each study site and there will be no overlap of subject numbers between sites. Screening CRFs will therefore be provided by GSK Biologicals to the study sites with prefilled subject numbers***. Once consent is obtained, then per-protocol eligibility criteria will be checked, which will necessitate a physical examination and blood sampling for assessment of hematology, renal and liver function. This will be documented on clinic forms, which are prepared and filled in by the investigator. Each form will contain the subject number, information about the volunteer's date of birth, household, date of screening visit, medical history, HBV vaccination status physical and laboratory screening examination. After reviewing the medical history, physical examination and laboratory results, any reasons for non-eligibility will be documented in the CRF. **Amended (04 July 2006)**

The parent(s)/guardian(s) of children who have been consented for the study and found to be ineligible will receive a full explanation by a study clinician. Any clinically relevant finding will be treated appropriately by a physician. Where necessary the child will be referred to a specialist at KATH, Kumasi for evaluation and treatment as described in

Section 5.8. An overview of the laboratory assays can be found in Appendix D and further details can be found in KHRC and KCCR/SMS SOPs.

A study identification card will be prepared for the parent(s)/guardian(s) of each screened subject. At screening a photograph of each screened subject being held by their parent(s)/guardian(s) will be taken and attached to the study identification card. This card will also bear the name of the study to which the child is enrolled, and the child's subject number. Parents will be instructed to come to the hospital if their child is sick and to identify their child as study participants.

5.4. Vaccination process

The vaccines RTS,S/AS02D, RTS,S/AS01E and Rabipur® will be packaged in identical boxes and will be identified by a treatment number. This unique treatment number will identify all doses of these vaccines administered to each subject. After randomization the treatment number assigns the subject to one vaccine group or another in a blinded way. Each subject will retain the same treatment number for their subsequent vaccine doses. The treatment number will be recorded on the subject's Clinic Form after the vaccine has been administered; information from the Clinic Form is subsequently transferred to the Case Report Form. The Clinic Form and Case Report Form link the subject number and the treatment number.

All vaccines will be administered by the intramuscular route to the left deltoid. Subjects who receive their vaccination in the incorrect arm will continue to receive subsequent blinded vaccinations as normal. The fact that the vaccine was administered in the wrong arm will be documented in clinic forms and CRF.

Vaccinations will take place at the outpatient clinic of KHRC, Kintampo and the maternal child health clinic (MCH) in Agogo, located close to the hospital. All vaccinations will be given by a qualified person; a nurse or a doctor. A staff member experienced in the resuscitation of children will be available at all vaccination sessions. Facilities and equipment will be available to give emergency treatment in the case of an anaphylactic reaction following administration of vaccines. All children will be observed for an hour after the administration of vaccine to evaluate and treat any acute adverse events.

The process for each vaccination is as follows. The identity of the child will be confirmed using the study identification card. The subject number on this card will be cross-checked with that on the subject's clinic form, ensuring that the subject number on the clinic form matches that of the study identification card. On the day of the first vaccination the contraindications to vaccination, inclusion/exclusion criteria and consent form will be checked prior to vaccination. On the days of the subsequent vaccinations, elimination criteria and contraindications to vaccination will be checked prior to vaccination. A vaccine clinic form will be initiated, which will give the child's identifiers, subject number and treatment number assigned after the administration of the vaccine.

Vaccines will be administered in an observer-blinded fashion. For each vaccination during the course of the study, the Vaccine Preparer will prepare the vaccine and the Vaccinator administer the vaccine for a specific subject. Since the vaccines used in this study are of distinct appearance, the Vaccine Preparers are not blinded and together with

the Vaccinators perform no other function in the study (refer to Section 6.5). The Vaccine Preparer will select the sealed box labeled with the appropriate treatment number, (containing the vials numbered with the treatment number), remove the vaccine vials and fill a syringe according to this study protocol (refer to Section 6.2). The Vaccine Preparer will then place a numbered opaque label with the subject's treatment number on the syringe. The purpose of masking the syringe is to blind the parent(s) or guardians(s) of the subject. The Vaccine Preparer will then pass the syringe to the Vaccinator in an adjacent room who will administer the vaccination. After administering the vaccination to the subject the Vaccinator will enter the treatment number administered to the subject on the clinic form.

Subjects who cannot be vaccinated on the originally scheduled date (see Section 4.6.1) will be vaccinated within 7 days and undergo all study procedures for the visit on the same day as vaccination. In the particular case of any child found to be febrile (axillary temperature ≥ 37.5°C), a blood slide will be taken to investigate for malaria. Children will be treated as appropriate for their condition and will be followed up until resolution of any symptoms and be vaccinated if their clinical symptoms resolve within 7 days.

Those who cannot be re-vaccinated within 7 days of their scheduled date will continue all study procedures apart from receiving further study vaccinations.

5.5. Home follow-up visits for assessment of reactogenicity (7-day follow-up period)

Trained field workers under the supervision of the Principal Investigators will visit each enrolled child at daily intervals for Days 1 to 5 post the first vaccination; a study clinician will examine the child in the clinic on Day 6 post first vaccination. For all other doses, the field workers will visit each enrolled child at daily intervals for Days 1 to 6 post each vaccination (see below in detailed study procedure; Section 5.11). In the event that the field worker finds any Grade 3 solicited general or unsolicited symptoms, the volunteer will be brought to the clinic for examination by a study clinician. Any further clinical data, including treatment provided, will be written on diary cards and clinic forms and transcribed onto the CRF. If the physician finds that the volunteer has experienced an SAE the appropriate measures will be taken to report this (See Section 8.7).

Diary cards will be checked and verified by the Principal Investigators or designate before transcription onto CRFs after the 7-day follow-up. The Principal Investigators have a primary responsibility for the data transcribed onto the CRFs. Unresolved AEs will be followed-up by field workers until resolution under the supervision of the Principal Investigator and data will be entered onto the CRF. The procedures and frequency of visits will be outlined in an SOP at the investigators' sites.

Analgesics/antipyretics will be provided to trained field workers for the treatment of children with injection site pain and fever and their use will be documented. Parent(s)/guardian(s) will not routinely be provided with these medications.

5.6. Monitoring of hematological and biochemical laboratory parameters

Hematological and biochemical parameters will be documented on the CRF. For all values outside the acceptable limit (refer to Table 18), the reason and/or clinical condition will be documented. Results of hematological and biochemical laboratory tests will be reviewed as soon as they are generated. Any value outside the normal range will be managed as appropriate by a medically qualified individual under the supervision of the Principal Investigator (refer to Section 8.6). Guidance on when to report abnormalities as SAEs is given in Section 8.3.

5.7. Surveillance for SAEs (all subjects)

5.7.1. At health facilities

Morbidity surveillance will be in place for this trial. The source of primary outpatient and inpatient care for the study participants in Kintampo and Agogo will be, respectively, the Kintampo District Hospital (see Section 4.1.2.2) and the Agogo Presbyterian Hospital (see Section 4.1.1.2). The study sites surveillance systems will provide a comprehensive recording of all outpatient attendances, the investigational results, diagnosis and management. Prior to the start of the study, all parent(s)/guardian(s) of volunteers will be educated on the appropriate action they should take if their child becomes unwell at any time during the study period. They will be asked not to medicate their child at home, but to seek medical care at the clinic.

A clinically qualified person will be available 24 hours per day at both hospitals to attend study participants when they present and to ensure complete investigation and documentation of the attendance.

All children attending as outpatients within the age range of the trial will be asked if the child is in this study and to provide the child's vaccine study identity card (see Section 5.3.2 for details of identity cards). If the identity cards are not available, the identity of the child will be confirmed against details collected at enrollment to the trial.

The child will be fully assessed by an appropriately clinically qualified person. This assessment will be recorded on hospital documents. These documents will provide a record of the child's name and study number, key symptoms and signs, axillary temperature, results of the laboratory tests and imaging examinations available at the time of form completion, the diagnosis, the treatment prescribed and will state whether hospital admission was required.

These forms will be reviewed by the Principal Investigators or their delegate. If it is necessary to notify any of these consultations as an SAE, the Principal Investigator or delegate will review the child as necessary and complete an SAE form to notify the event (as specified in Section 8.5).

Children requiring inpatient care will be admitted to the Kintampo District Hospital or Agogo Presbyterian Hospital. Their clinical course, treatment and results of further

investigations will be documented in their hospital records. The hospital records and inpatient surveillance will be the source documents for the SAE reports. When necessary, children will be followed to resolution of SAEs as outpatients.

5.7.2. In the community

Up to Clinic Visit 9 volunteers will be seen at least monthly at clinic visits or by field workers, and for 6 days following each dose by fieldworkers. After Clinic Visit 9, capture of SAEs will be enhanced by means of monthly visits by field workers and a final Clinic Visit at Month 19 (Clinic Visit 10)

During the field worker visits, the children's parent(s)/guardian(s) will be asked retrospectively if any SAEs occurred since the last visit and this information will be recorded. Unreported SAEs detected in this way will be investigated and reported by the Principal Investigators or their delegate on the corresponding SAE forms (see Section 5.8). In the case of a death which has occurred at home, supplementary information will be gained using the verbal autopsy technique. The verbal autopsy will be conducted according to previously published methods and detailed in the SOPs on file with the investigators [Smith 1991].

If any child is reported to be unwell at the time of a visit, the field worker will advise the parent(s)/guardian(s) to seek care at the clinic. In the event that a child is seriously ill, the field worker will inform the Principal Investigators or their designate, and transport will be arranged to clinic, if judged appropriate by the responsible clinician.

5.8. Provision of health care

For children in the trial, all primary health care will be conducted by physicians at Kintampo District Hospital and Agogo Presbyterian Hospital. This system will capture AEs up to 30 days post each vaccination and unsolicited SAEs during the whole course of the trial.

Contact details for the relevant PI will be provided to parent(s)/guardian(s) on the ICF. Medical attention is available on a 24-hour basis, seven days a week at the clinics. Children requiring inpatient care will be admitted to the ward. Laboratory and radiological investigation will be carried out when appropriate. Treatment for medical conditions will be given according to the standard treatment regimens of Ghana. A detailed description of the healthcare system available in the study area is provided in Section 4.1.1.2 for KCCR/SMS and Section 4.1.2.2 for KHRC. Children requiring specialized care or investigation unavailable locally will be transported to KATH, Kumasi. In case of referral, all history, physical examination and laboratory findings available will be provided to the parent(s)/guardian(s) and the referral physician.

Any expenses — including transport — incurred by the parents/guardians of study participants for the purpose of obtaining a diagnosis of an adverse event as well as for clinical care related to acute conditions will be borne by the study. Long-term care for chronic conditions unrelated to study procedures will be delivered following local guidelines with no financial support from the study.

5.8.1. Management and treatment of malaria in all subjects

All children with suspected malaria will have a blood sample taken for confirmation of infection by blood smear reading. Children with malaria that can be treated with oral medication will receive a 6-dose regimen of artesunate/amodiaquine, as advocated by the Ministry of Health of Ghana.

Children who require inpatient care or systemic treatment will receive treatment with intravenous or intramuscular quinine, which is standard practice, recommended by WHO and effective therapy in Ghana.

5.8.2. Measles and Yellow Fever vaccination

The EPI of WHO recommends administration of Yellow Fever vaccine and Measles vaccine to children at 9 months of age in Ghana. Vaccination with trial vaccines will be planned so as to ensure that administration of trial vaccines to children (i.e. RTS,S/AS02D, RTS,S/AS01E or Rabies vaccine) does not interfere with the administration of EPI vaccines. No trial vaccination will be administered within 14 days of an EPI vaccine (refer to Sections 4.4, 4.5 and 4.6).

5.8.3. Referring of excluded volunteers to medical care

If a volunteer is found to have a medical condition that excludes them from the trial, their parent(s)/guardian(s) will be informed at a private appointment with a member of the clinical staff of the research team. The clinical staff member will take as much time as is required to explain the condition, including its severity, potential causes, long-term implications, impact on current and future lifestyle of both the child and the parent(s)/guardian(s) and evaluation and treatment options. Once it is clear that the parent(s)/guardian(s) understand the medical condition, the clinical staff member will develop an evaluation and treatment plan with the parent(s)/guardian(s) and ensure that the options are understood. If necessary, children will be transported to KATH, Kumasi.

Any expenses — including transport — incurred by the parents/guardians of study participants for the purpose diagnosis as well as for clinical care related to an acute conditions will be borne by the study. Long-term care for chronic conditions unrelated to study procedures will be delivered following local guidelines with no financial support from the study.

5.9. Subject identification

Subject numbers will be issued sequentially to subjects at screening. Treatment numbers are also assigned sequentially to eligible children at the time of Dose 1 starting with the lowest number in order of administration of dose.

Subject numbers will be issued consecutively to all children who are consented for screening. To identify the child at subsequent contacts, each child’s parent(s)/guardian(s) will be issued with an identification card bearing the child’s subject number with the picture of the parent(s)/guardian(s) with the child attached to it.

Vials of RTS,S/AS02D, RTS,S/AS01E and Rabies vaccine will be identified by a treatment number. The unique treatment number will identify all doses of the vaccine administered to each subject. The treatment number will be issued sequentially at Clinic Visit 2. The treatment number will be recorded on the clinic form and subsequently the case report form documenting the linkage between the subject number and the treatment number.

5.10. Outline of study procedures

Table 5 List of study procedures: children to receive RTS,S/AS01E or RTS,S/AS02D on a 0, 1-month schedule

	SCREEN	PRIMARY STUDY PHASE												EXTENDED FOLLOW-UP	
**Study Month**		**Month 0**			**Month 1**		**Month 2**	**Month 3**	**Months 4 to 6**	**Month 7**	**Month 8**	**Month 9**	**Month 10**	**Months 11 to 18**	**Month 19**
**Study Day**	**-28 to 0**	**0**	**1 to 5**	**6**	**30**	**31 to 36**	**60**	**90**							
**Clinic Visit**	**1**	**2**		**3**	**4**		**5**	**6**		**7**	**8**		**9**		**10**
**Field worker Visit code #**			**21 to 25**			**26 to 31**			**38 to 40**			**47**		**48 to 55**	
**STUDY PROCEDURES**															
Informed consent	●														
Medical History	○	●			○		○	○		○	○		○		○
Vital Signs	○	●			○		○	○		○	○		○		○
Complete physical examination	○	●		○	○		○	○		○	○		○		○
Measure body weight	○	●			○		○	○		○	○		○		○
Assign Subject Number	●														
Prevaccination temperature		●			●										
Check inclusion/exclusion criteria	●	○													
Check elimination criteria		●		●	●		●	●		●	●		●		●
Check contraindications to vaccination		●			●										
Randomization		●													
Administer RTS,S/AS02D or RTS,S/AS01E		●			●										
Recording of concomitant medication		● [c]	● [c]	● [c]	● [c]	● [c]	● [c]	● [c]	● [c]	● [c]	● [c]	● [c]	● [c]	● [d]	● [d]
**SAFETY DATA COLLECTION**															
Recording of solicited symptoms (Investigator)		●		●	●										
Recording of solicited symptoms (Field Workers)			●			●									
Recording of unsolicited AEs within 1 month post-vaccination (Investigator)		●		●	●		●								
Morbidity surveillance/recording of SAEs	● [b]	●	●	●	●	●	●	●	●	●	●	●	●	●	●
**SAFETY LABS**															
Complete blood count [a]	●			● [e]			●			●			●		●
Creatinine, ALT	●			● [e]			●			●			●		●
**INVESTIGATIONAL ASSAYS**															
Antibodies to CS	●						●			●			●		●
Antibodies to HBs	●						●								●
Cell-Mediated Immunity							●								●
**FINAL ANALYSIS**													●		
**STUDY CONCLUSION**															●

Please refer to footnotes following Table 7

Table 6 List of study procedures: children to receive RTS,S/AS01E or Rabies vaccine *or RTS,S/AS02D* on a 0, 1, 2-month schedule

	SCREEN	PRIMARY STUDY PHASE													EXTENDED FOLLOW-UP	
**Study Month**		**Month 0**			**Month 1**		**Month 2**		**Month 3**	**Months 4 to 6**	**Month 7**	**Month 8**	**Month 9**	**Month 10**	**Months 11 to 18**	**Month 19**
**Study Day**	**-28 to 0**	**0**	**1 to 5**	**6**	**30**	**31 to 36**	**60**	**61 to 66**	**90**							
**Clinic Visit**	**1**	**2**		**3**	**4**		**5**		**6**		**7**	**8**		**9**		**10**
**Field worker Visit code #**			**21 to 25**			**26 to 31**		**32 to 37**		**38 to 40**			**47**		**48 to 55**	
**STUDY PROCEDURES**																
Informed consent	●															
Medical History	○	●			○		○		○		○	○		○		○
Vital Signs	○	●			○		○		○		○	○		○		○
Complete physical examination	○	●		○	○		○		○		○	○		○		○
Measure body weight	○	●			○		○		○		○	○		○		○
Assign Subject Number	●															
Prevaccination temperature		●			●		●									
Check inclusion/exclusion criteria	●	○														
Check elimination criteria		●		●	●		●		●		●	●		●		●
Check contraindications to vaccination		●			●		●									
Randomization		●														
Administer RTS,S/AS01E or Rabipur Rabies vaccine ***or RTS,S/AS02D***[1]		●			●		●									
Recording of concomitant medication		● [c]	● [c]	● [c]	● [c]	● [c]	● [c]	● [c]	● [c]	● [c]	● [c]	● [c]	● [c]	● [c]	● [d]	● [d]
**SAFETY DATA COLLECTION**																
Recording of solicited symptoms (Investigator)		●		●	●		●									
Recording of solicited symptoms (Field Workers)			●			●		●								
Recording of unsolicited AEs within 1 month post-vaccination (Investigator)		●		●	●		●		●							
Morbidity surveillance/recording of SAEs	● [b]	●	●	●	●	●	●	●	●	●	●	●	●	●	●	●
**SAFETY LABS**																
Complete blood count [a]	●			● [e]					●		●			●		●
Creatinine, ALT	●			● [e]					●		●			●		●
**INVESTIGATIONAL ASSAYS**																
Antibodies to CS	●								●		●			●		●
Antibodies to HBs	●								●							●
Cell-Mediated Immunity									●							●
**FINAL ANALYSIS**														●		
**STUDY CONCLUSION**																●

Please refer to footnotes following Table 7

1 The Rabies vaccine will only be used at Kintampo-KHRC; The RTS,S/AS02D vaccine will be used at Kumasi-KCCR/SMS.

Amended (04 July 2006)

Table 7 List of study procedures: children to receive RTS,S/AS01E or RTS,S/AS02D on a 0, 1, 7-month schedule

	SCREEN	PRIMARY STUDY PHASE													EXTENDED FOLLOW-UP	
**Study Month**		Month 0			Month 1		Month 2	Month 3	Months 4 to 6	Month 7		Month 8	Month 9	Month 10	Months 11 to 18	Month 19
**Study Day**	-28 to 0	0	1 to 5	6	30	31 to 36	60	90								
**Clinic Visit**	1	2		3	4		5	6		7		8		9		10
**Field worker Visit code #**			21 to 25			26 to 31			38 to 40		41 to 46		47		48 to 55	
**STUDY PROCEDURES**																
Informed consent	●															
Medical History	○	●			○		○	○		○		○		○		○
Vital Signs	○	●			○		○	○		○		○		○		○
Complete physical examination	○	●		○	○		○	○		○		○		○		○
Measure body weight	○	●			○		○	○		○		○		○		○
Assign Subject Number	●															
Prevaccination temperature		●			●					●						
Check inclusion/exclusion criteria	●	○														
Check elimination criteria		●		●	●		●	●		●		●		●		●
Check contraindications to vaccination		●			●					●						
Randomization		●														
Administer RTS,S/AS02D or RTS,S/AS01E		●			●					●						
Recording of concomitant medication		● [c]	● [c]	● [c]	● [c]	● [c]	● [c]	● [c]	● [c]	● [c]	● [c]	● [c]	● [c]	● [c]	● [d]	● [d]
**SAFETY DATA COLLECTION**																
Recording of solicited symptoms (Investigator)		●		●	●					●						
Recording of solicited symptoms (Field Workers)			●			●					●					
Recording of unsolicited AEs within 1 month post-vaccination (Investigator)		●		●	●		●					●				
Morbidity surveillance/recording of SAEs	● [b]	●	●	●	●	●	●	●	●	●	●	●	●	●	●	●
**SAFETY LABS**																
Complete blood count [a]	●			● [e]						●		●		●		●
Creatinine, ALT	●			● [e]						●		●		●		●
**INVESTIGATIONAL ASSAYS**																
Antibodies to CS	●									●		●		●		●
Antibodies to HBs	●											●				●
Cell-Mediated Immunity												●				●
**FINAL ANALYSIS**														●		
**STUDY CONCLUSION**																●

Please refer to footnotes following this table…

Footnotes for Table 5, Table 6 and Table 7:

#: Field Worker visit code numbers are designated in order from the beginning to the end of the study period; they are not necessarily sequential. • is used to indicate a study procedure that requires documentation in the individual CRF and ○ is used to indicate a study procedure that does not require documentation in the individual CRF.
a: includes analysis of hemoglobin, total white cell count and platelets
b: SAEs related to study procedures will be collected
c: all medications required by protocol to be recorded at these visits
d: record administration of immunosuppressants or other immune-modifying drugs during this period (for corticosteroids this means prednisone or equivalent, ≥ 0.5 mg/kg/day. Inhaled or topical steroids are allowed and should not be recorded. ALSO all immunoglobulins and blood products should be recorded during this period.
e: only to be carried out on the first 100 children to present for this visit

It is the investigator's responsibility to ensure that the intervals between visits/contacts are strictly followed. These intervals determine each subject's evaluability in the according to protocol analyses (See Sections 4.3 to 4.6 and Section 10.5.2 for details of criteria for evaluability and cohorts to be analyzed). The intervals are tabulated in Table 8, Table 9 and Table 10.

Table 8 Intervals between study stages/visits for children to receive RTS,S/AS01E or RTS,S/AS02D on a 0, 1-month schedule

Interval	Length of interval
(Visit 1→Visit 2)	0 to 28 days
(Visit 2→Visit 4)	21 to 35 days
(Visit 4→Visit 5)	21 to 35 days
(Visit 2→Visit 9)	10 months ± 1 month
(Visit 2→Visit 10)	19 months ± 1 month

Table 9 Intervals between study stages/visits for children to receive RTS,S/AS01E or Rabies vaccine *or RTS,S/AS02D* on a 0, 1, 2-month schedule Amended (04 July 2006)

Interval	Length of interval
(Visit 1→Visit 2)	0 to 28 days
(Visit 2→Visit 4)	21 to 35 days
(Visit 4→Visit 5)	21 to 35 days
(Visit 5→Visit 6)	21 to 35 days
(Visit 2→Visit 9)	10 months ± 1 month
(Visit 2→Visit 10)	19 months ± 1 month

Table 10 Intervals between study stages/visits for children to receive RTS,S/AS01E or RTS,S/AS02D on a 0, 1, 7-month schedule

Interval	Length of interval
(Visit 1→Visit 2)	0 to 28 days
(Visit 2→Visit 4)	21 to 35 days
(Visit 4→Visit 5)	21 to 35 days
(Visit 4→Visit 7)	6 months ± 2 weeks
(Visit 7→Visit 8)	21 to 35 days
(Visit 2→Visit 9)	10 months ± 1 month
(Visit 2→Visit 10)	19 months ± 1 month

5.11. Detailed description of study stages/visits

When materials are provided by GSK Biologicals, it is **MANDATORY** that all clinical samples (including serum samples) will be collected and stored using exclusively those materials in the appropriate manner. The use of other materials could result in the exclusion of the subject from the ATP analysis (See Section 10.5.2 for definition of study cohorts to be evaluated). The investigator must ensure that his/her personnel and the laboratory(ies) under his/her supervision comply with this requirement. However, when GSK Biologicals does not provide material for collecting and storing clinical samples, then appropriate materials from the investigator's site are to be used. Refer to Appendix D and Appendix E.

The vaccinees will be observed closely for at least 60 minutes, with appropriate medical treatment readily available in case of an anaphylactic reaction following the administration of vaccines.

The subjects' parents/guardians will be instructed to contact the investigator immediately should they/ the subject manifest any signs or symptoms they perceive as serious.

Table 11 Summary of blood sampling timepoints and volumes to be collected; children to receive RTS,S/AS01E or RTS,S/AS02D on a 0, 1-month schedule

Sampling Timepoint	Tests to be carried out	Total volume of blood required
**CV 1** (Screening)	Safety labs<br>Antibodies to CS<br>Antibodies to HBs	1 mL
**CV 3***	Safety labs*	1 mL*
**CV 5**	Safety labs<br>Antibodies to CS<br>Antibodies to HBs<br>CMI	5 mL
**CV 7**	Safety labs<br>Antibodies to CS	1 mL
**CV 9**	Safety labs<br>Antibodies to CS	1 mL
**CV 10**	Safety labs<br>Antibodies to CS<br>Antibodies to HBs<br>CMI	5 mL

* This test only to take place on the first 100 children vaccinated
CV: Clinic Visit **CMI:** Cell Mediated Immunity

Table 12 Summary of blood sampling timepoints and volumes to be collected; children to receive RTS,S/AS01E or Rabies vaccine *or RTS,S/AS02D* on a 0, 1, 2-month schedule

Sampling Timepoint	Tests to be carried out	Total volume of blood required
**CV 1** (Screening)	Safety labs<br>Antibodies to CS<br>Antibodies to HBs	1 mL
**CV 3***	Safety labs*	1 mL*
**CV 6**	Safety labs<br>Antibodies to CS<br>Antibodies to HBs<br>CMI	5 mL
**CV 7**	Safety labs<br>Antibodies to CS	1 mL
**CV 9**	Safety labs<br>Antibodies to CS	1 mL
**CV 10**	Safety labs<br>Antibodies to CS<br>Antibodies to HBs<br>CMI	5 mL

* This test only to take place on the first 100 children vaccinated
CV: Clinic Visit **CMI:** Cell Mediated Immunity

Amended (04 July 2006)

Table 13 Summary of blood sampling timepoints and volumes to be collected; children to receive RTS,S/AS01E or RTS,S/AS02D on a 0, 1, 7-month schedule

Sampling Timepoint	Tests to be carried out	Total volume of blood required
**CV 1** (Screening)	Safety labs<br>Antibodies to CS<br>Antibodies to HBs	1 mL
**CV 3***	Safety labs*	1 mL*
**CV 7**	Safety labs<br>Antibodies to CS	1 mL
**CV 8**	Safety labs<br>Antibodies to CS<br>Antibodies to HBs<br>CMI	5 mL
**CV 9**	Safety labs<br>Antibodies to CS	1 mL
**CV 10**	Safety labs<br>Antibodies to CS<br>Antibodies to HBs<br>CMI	5 mL

* This test only to take place on the first 100 children vaccinated
CV: Clinic Visit **CMI:** Cell Mediated Immunity

5.11.1. Study visits for children to receive RTS,S/AS01E or RTS,S/AS02D on a 0, 1-month schedule

Clinic Visit 1: Screening
Day -28 to Day 0

- Obtain signed, dated, thumb printed informed consent from the parent(s)/guardians
- Check inclusion/exclusion criteria
- Carry out physical examination, take medical history, measure body weight, record vital signs
- Assign subject number
- Record any SAEs that may have occurred as a result of study procedures
- Venous blood sample to collect 1 mL blood for analysis of:
  - hematology (complete blood count)
  - biochemistry (creatinine and ALT)
  - serology (antibodies to CS, HBs)

Clinic Visit 2: Vaccination 1
Day 0

- Check study identification card of vaccinee
- Carry out physical examination, take medical history, measure body weight, record vital signs
- Check inclusion/exclusion criteria
- Check contraindications to vaccination
- Check elimination criteria
- Record pre-vaccination body temperature
- Randomize subjects
- Administer the first dose of RTS,S/AS01E or RTS,S/AS02D intramuscularly in the left deltoid

Each child will be assessed for at least 60 minutes after vaccination to evaluate and treat any acute adverse events.

- Record any post-vaccination solicited symptoms
- Record any post-vaccination unsolicited adverse events
- Record any post-vaccination SAEs
- Record concomitant medication

Field worker post vaccination follow-up visits 21 to 25
Days 1, 2, 3, 4, and 5

- Check study identification card of vaccinee
- Record axillary temperature of subject
- Record local (pain and swelling at the injection site) and general (fever, irritability / fussiness, drowsiness, loss of appetite) solicited adverse events
- Record SAEs experienced by the vaccinee since the last visit
- Record unsolicited adverse events experienced by the vaccinee since the last visit
- Record concomitant medication

Clinic Visit 3: Post Dose 1 follow-up visit in Clinic
Day 6

- Check study identification card of vaccinee
- Carry out physical examination
- Record axillary temperature of subject
- Record local (pain and swelling at the injection site) and general (fever, irritability / fussiness, drowsiness, loss of appetite) solicited adverse events
- Record SAEs experienced by the vaccinee since the last visit
- Record unsolicited adverse events experienced by the vaccinee since the last visit
- Check elimination criteria
- Record concomitant medication
- Blood sample to collect a minimum 1 mL blood for analysis of:
  - hematology (complete blood count)
  - biochemistry (creatinine and ALT)

Clinic Visit 4: Vaccination 2
Day 30

- Check study identification card of vaccinee
- Carry out physical examination, take medical history, measure body weight, record vital signs
- Check contraindications to vaccination
- Check elimination criteria
- Record unsolicited adverse events experienced by the vaccinee since the last visit
- Record pre-vaccination body temperature
- Administer the second dose of RTS,S/AS01E or RTS,S/AS02D intramuscularly in the left deltoid

Each child will be assessed for at least 60 minutes after vaccination to evaluate and treat any acute adverse events.

- Record any post-vaccination solicited symptoms
- Record any post-vaccination unsolicited adverse events
- Record any post-vaccination SAEs
- Record concomitant medication

Field worker post vaccination follow-up visits 26 to 31
Days 31, 32, 33, 34, 35 and 36

- Check study identification card of vaccinee
- Record axillary temperature of subject
- Record local (pain and swelling at the injection site) and general (fever, irritability / fussiness, drowsiness, loss of appetite) solicited adverse events
- Record SAEs experienced by the vaccinee since the last visit
- Record unsolicited adverse events experienced by the vaccinee since the last visit
- Record concomitant medication

Clinic Visit 5
Day 60

- Check study identification card of vaccinee
- Carry out physical examination, take medical history, measure body weight, record vital signs
- Record axillary temperature of subject
- Record SAEs experienced by the vaccinee since the last visit
- Record unsolicited adverse events experienced by the vaccinee since the last visit
- Check elimination criteria
- Record concomitant medication
- Blood sample to collect 5 mL blood for analysis of:
  - hematology (complete blood count)
  - biochemistry (creatinine and ALT)
  - serology (antibodies to CS, HBs)
  - cell-mediated immunity

Clinic Visit 6
Day 90

- Check study identification card of vaccinee

- Carry out physical examination, take medical history, measure body weight, record vital signs
- Record axillary temperature of subject
- Record SAEs experienced by the vaccinee since the last visit
- Check elimination criteria
- Record concomitant medication

Field worker follow-up visits 38, 39, 40
Months 4, 5, 6

- Check study identification card of vaccinee
- Record axillary temperature of subject
- Record SAEs experienced by the vaccinee since the last visit
- Record concomitant medication

Clinic Visit 7
Month 7

- Check study identification card of vaccinee
- Carry out physical examination, take medical history, measure body weight, record vital signs
- Record axillary temperature of subject
- Record SAEs experienced by the vaccinee since the last visit
- Check elimination criteria
- Record concomitant medication
- Blood sample to collect 1 mL blood for analysis of:
  - hematology (complete blood count)
  - biochemistry (creatinine and ALT)
  - serology (antibodies to CS)

Clinic Visit 8
Month 8

- Check study identification card of vaccinee
- Carry out physical examination, take medical history, measure body weight, record vital signs
- Record axillary temperature of subject
- Record SAEs experienced by the vaccinee since the last visit
- Check elimination criteria

- Record concomitant medication

Field worker follow-up visit 47
Month 9

- Check study identification card of vaccinee
- Record axillary temperature of subject
- Record SAEs experienced by the vaccinee since the last visit
- Record concomitant medication

Clinic Visit 9
Month 10

- Check study identification card of vaccinee
- Carry out physical examination, take medical history, measure body weight, record vital signs
- Record axillary temperature of subject
- Record SAEs experienced by the vaccinee since the last visit
- Check elimination criteria
- Record concomitant medication
- Blood sample to collect 1 mL blood for analysis of:
  - hematology (complete blood count)
  - biochemistry (creatinine and ALT)
  - serology (antibodies to CS)

Field worker follow-up visit 48, 49, 50, 51, 52, 53, 54, 55
Months 11, 12, 13, 14, 15, 16, 17, 18

- Check study identification card of vaccinee
- Record axillary temperature of subject
- Record SAEs experienced by the vaccinee since the last visit
- Record concomitant medication

Clinic Visit 10
Month 19

- Check study identification card of vaccinee
- Carry out physical examination, take medical history, measure body weight, record vital signs
- Record axillary temperature of subject
- Record SAEs experienced by the vaccinee since the last visit

- Check elimination criteria
- Record concomitant medication
- Blood sample to collect 5 mL blood for analysis of:
  - hematology (complete blood count)
  - biochemistry (creatinine and ALT)
  - serology (antibodies to CS, HBs)
  - cell-mediated immunity

5.11.2. Study visits for children to receive RTS,S/AS01E or Rabies vaccine *or RTS,S/AS02D* on a 0, 1, 2-month schedule

Amended (04 July 2006)

Clinic Visit 1: Screening
Week -28 to Day 0

- Obtain signed, dated, thumb printed informed consent from the parent(s)/guardians
- Check inclusion/exclusion criteria
- Carry out physical examination, take medical history, measure body weight, record vital signs
- Assign subject number
- Record any SAEs that may have occurred as a result of study procedures
- Venous blood sample to collect 1 mL blood for analysis of:
  - hematology (complete blood count)
  - biochemistry (creatinine and ALT)
  - serology (antibodies to CS, HBs)

Clinic Visit 2: Vaccination 1
Day 0

- Check study identification card of vaccinee
- Carry out physical examination, take medical history, measure body weight, record vital signs
- Check inclusion/exclusion criteria
- Check contraindications to vaccination
- Check elimination criteria
- Record pre-vaccination body temperature
- Randomize subjects

- Administer the first dose of RTS,S/AS01E or Rabies Vaccine ***or RTS,S/AS02D*** intramuscularly in the left deltoid **Amended (04 July 2006)**

Each child will be assessed for at least 60 minutes after vaccination to evaluate and treat any acute adverse events.

- Record any post-vaccination solicited symptoms
- Record any post-vaccination unsolicited adverse events
- Record any post-vaccination SAEs
- Record concomitant medication

Field worker post vaccination follow-up visits 21 to 25
Days 1, 2, 3, 4, and 5

- Check study identification card of vaccinee
- Record axillary temperature of subject
- Record local (pain and swelling at the injection site) and general (fever, irritability / fussiness, drowsiness, loss of appetite) solicited adverse events
- Record SAEs experienced by the vaccinee since the last visit
- Record unsolicited adverse events experienced by the vaccinee since the last visit
- Record concomitant medication

Clinic Visit 3: Post Dose 1 follow-up visit in Clinic
Day 6

- Check study identification card of vaccinee
- Carry out physical examination
- Record axillary temperature of subject
- Record local (pain and swelling at the injection site) and general (fever, irritability / fussiness, drowsiness, loss of appetite) solicited adverse events
- Record SAEs experienced by the vaccinee since the last visit
- Record unsolicited adverse events experienced by the vaccinee since the last visit
- Check elimination criteria
- Record concomitant medication
- Blood sample to collect a minimum 1 mL blood for analysis of:
  - hematology (complete blood count)
  - biochemistry (creatinine and ALT)

Clinic Visit 4: Vaccination 2
Day 30

- Check study identification card of vaccinee
- Carry out physical examination, take medical history, measure body weight, record vital signs
- Check contraindications to vaccination
- Check elimination criteria
- Record unsolicited adverse events experienced by the vaccinee since the last visit
- Record pre-vaccination body temperature
- Administer the second dose of RTS,S/AS01E or Rabies Vaccine ***or RTS,S/AS02D*** intramuscularly in the left deltoid **Amended (04 July 2006)**

Each child will be assessed for at least 60 minutes after vaccination to evaluate and treat any acute adverse events.

- Record any post-vaccination solicited symptoms
- Record any post-vaccination unsolicited adverse events
- Record any post-vaccination SAEs
- Record concomitant medication

Field worker post vaccination follow-up visits 26 to 31
Days 31, 32, 33, 34, 35 and 36

- Check study identification card of vaccinee
- Record axillary temperature of subject
- Record local (pain and swelling at the injection site) and general (fever, irritability / fussiness, drowsiness, loss of appetite) solicited adverse events
- Record SAEs experienced by the vaccinee since the last visit
- Record unsolicited adverse events experienced by the vaccinee since the last visit
- Record concomitant medication

Clinic Visit 5: Vaccination 3
Day 60

- Check study identification card of vaccinee
- Carry out physical examination, take medical history, measure body weight, record vital signs
- Check contraindications to vaccination
- Check elimination criteria
- Record unsolicited adverse events experienced by the vaccinee since the last visit

- Record pre-vaccination body temperature
- Administer the third dose of RTS,S/AS01E or Rabies Vaccine ***or RTS,S/AS02D*** intramuscularly in the left deltoid **Amended (04 July 2006)**

Each child will be assessed for at least 60 minutes after vaccination to evaluate and treat any acute adverse events.

- Record any post-vaccination solicited symptoms
- Record any post-vaccination unsolicited adverse events
- Record any post-vaccination SAEs
- Record concomitant medication

Field worker post vaccination follow-up visits 32 to 37
Days 61, 62, 63, 64, 65 and 66

- Check study identification card of vaccinee
- Record axillary temperature of subject
- Record local (pain and swelling at the injection site) and general (fever, irritability / fussiness, drowsiness, loss of appetite) solicited adverse events
- Record SAEs experienced by the vaccinee since the last visit
- Record unsolicited adverse events experienced by the vaccinee since the last visit
- Record concomitant medication

Clinic Visit 6
Day 90

- Check study identification card of vaccinee
- Carry out physical examination, take medical history, measure body weight, record vital signs
- Record axillary temperature of subject
- Record SAEs experienced by the vaccinee since the last visit
- Record unsolicited adverse events experienced by the vaccinee since the last visit
- Check elimination criteria
- Record concomitant medication
- Blood sample to collect 5 mL blood for analysis of:
  - hematology (complete blood count)
  - biochemistry (creatinine and ALT)
  - serology (antibodies to CS, HBs)
  - cell-mediated immunity

Field worker follow-up visits 38, 39, 40
Months 4, 5, 6

- Check study identification card of vaccinee
- Record axillary temperature of subject
- Record SAEs experienced by the vaccinee since the last visit
- Record concomitant medication

Clinic Visit 7
Month 7

- Check study identification card of vaccinee
- Carry out physical examination, take medical history, measure body weight, record vital signs
- Record axillary temperature of subject
- Record SAEs experienced by the vaccinee since the last visit
- Check elimination criteria
- Record concomitant medication
- Blood sample to collect 1 mL blood for analysis of:
  - hematology (complete blood count)
  - biochemistry (creatinine and ALT)
  - serology (antibodies to CS)

Clinic Visit 8
Month 8

- Check study identification card of vaccinee
- Carry out physical examination, take medical history, measure body weight, record vital signs
- Record axillary temperature of subject
- Record SAEs experienced by the vaccinee since the last visit
- Check elimination criteria
- Record concomitant medication

Field worker follow-up visit 47
Month 9

- Check study identification card of vaccinee
- Record axillary temperature of subject
- Record SAEs experienced by the vaccinee since the last visit

- Record concomitant medication

Clinic Visit 9
Month 10

- Check study identification card of vaccinee
- Carry out physical examination, take medical history, measure body weight, record vital signs
- Record axillary temperature of subject
- Record SAEs experienced by the vaccinee since the last visit
- Check elimination criteria
- Record concomitant medication
- Blood sample to collect 1 mL blood for analysis of:
  - hematology (complete blood count)
  - biochemistry (creatinine and ALT)
  - serology (antibodies to CS)

Field worker follow-up visit 48, 49, 50, 51, 52, 53, 54, 55
Months 11, 12, 13, 14, 15, 16, 17, 18

- Check study identification card of vaccinee
- Record axillary temperature of subject
- Record SAEs experienced by the vaccinee since the last visit
- Record concomitant medication

Clinic Visit 10
Month 19

- Check study identification card of vaccinee
- Carry out physical examination, take medical history, measure body weight, record vital signs
- Record axillary temperature of subject
- Record SAEs experienced by the vaccinee since the last visit
- Check elimination criteria
- Record concomitant medication
- Blood sample to collect 5 mL blood for analysis of:
  - hematology (complete blood count)
  - biochemistry (creatinine and ALT)
  - serology (antibodies to CS, HBs)

- cell-mediated immunity

5.11.3. Study visits for children to receive RTS,S/AS01E or RTS,S/AS02D on a 0, 1, 7-month schedule

Clinic Visit 1: Screening
Week -28 to Day 0

- Obtain signed, dated, thumb printed informed consent from the parent(s)/guardians
- Check inclusion/exclusion criteria
- Carry out physical examination, take medical history, measure body weight, record vital signs
- Assign subject number
- Record any SAEs that may have occurred as a result of study procedures
- Venous blood sample to collect 1 mL blood for analysis of:
  - hematology (complete blood count)
  - biochemistry (creatinine and ALT)
  - serology (antibodies to CS, HBs)

Clinic Visit 2: Vaccination 1
Day 0

- Check study identification card of vaccinee
- Carry out physical examination, take medical history, measure body weight, record vital signs
- Check inclusion/exclusion criteria
- Check contraindications to vaccination
- Check elimination criteria
- Record pre-vaccination body temperature
- Randomize subjects
- Administer the first dose of RTS,S/AS01E or RTS,S/AS02D intramuscularly in the left deltoid

Each child will be assessed for at least 60 minutes after vaccination to evaluate and treat any acute adverse events.

- Record any post-vaccination solicited symptoms
- Record any post-vaccination unsolicited adverse events
- Record any post-vaccination SAEs
- Record concomitant medication

Field worker post vaccination follow-up visits 21 to 25
Days 1, 2, 3, 4, and 5

- Check study identification card of vaccinee
- Record axillary temperature of subject
- Record local (pain and swelling at the injection site) and general (fever, irritability / fussiness, drowsiness, loss of appetite) solicited adverse events
- Record SAEs experienced by the vaccinee since the last visit
- Record unsolicited adverse events experienced by the vaccinee since the last visit
- Record concomitant medication

Clinic Visit 3: Post Dose 1 follow-up visit in Clinic
Day 6

- Check study identification card of vaccinee
- Carry out physical examination
- Record axillary temperature of subject
- Record local (pain and swelling at the injection site) and general (fever, irritability / fussiness, drowsiness, loss of appetite) solicited adverse events
- Record SAEs experienced by the vaccinee since the last visit
- Record unsolicited adverse events experienced by the vaccinee since the last visit
- Check elimination criteria
- Record concomitant medication
- Blood sample to collect a minimum 1 mL blood for analysis of:
  - hematology (complete blood count)
  - biochemistry (creatinine and ALT)

Clinic Visit 4: Vaccination 2
Day 30

- Check study identification card of vaccinee
- Carry out physical examination, take medical history, measure body weight, record vital signs
- Check contraindications to vaccination
- Check elimination criteria
- Record unsolicited adverse events experienced by the vaccinee since the last visit
- Record pre-vaccination body temperature
- Administer the second dose of RTS,S/AS01E or RTS,S/AS02D intramuscularly in the left deltoid

Each child will be assessed for at least 60 minutes after vaccination to evaluate and treat any acute adverse events.

- Record any post-vaccination solicited symptoms
- Record any post-vaccination unsolicited adverse events
- Record any post-vaccination SAEs
- Record concomitant medication

Field worker post vaccination follow-up visits 26 to 31
Days 31, 32, 33, 34, 35 and 36

- Check study identification card of vaccinee
- Record axillary temperature of subject
- Record local (pain and swelling at the injection site) and general (fever, irritability / fussiness, drowsiness, loss of appetite) solicited adverse events
- Record SAEs experienced by the vaccinee since the last visit
- Record unsolicited adverse events experienced by the vaccinee since the last visit
- Record concomitant medication

Clinic Visit 5
Day 60

- Check study identification card of vaccinee
- Carry out physical examination, take medical history, measure body weight, record vital signs
- Record axillary temperature of subject
- Record SAEs experienced by the vaccinee since the last visit
- Record unsolicited adverse events experienced by the vaccinee since the last visit
- Check elimination criteria
- Record concomitant medication

Clinic Visit 6
Day 90

- Check study identification card of vaccinee
- Carry out physical examination, take medical history, measure body weight, record vital signs
- Record axillary temperature of subject
- Record SAEs experienced by the vaccinee since the last visit
- Check elimination criteria

- Record concomitant medication

Field worker follow-up visits 38, 39, 40
Months 4, 5, 6

- Check study identification card of vaccinee
- Record axillary temperature of subject
- Record SAEs experienced by the vaccinee since the last visit
- Record concomitant medication

Clinic Visit 7: Vaccination 3
Month 7

- Check study identification card of vaccinee
- Carry out physical examination, take medical history, measure body weight, record vital signs
- Check contraindications to vaccination
- Check elimination criteria
- Record unsolicited adverse events experienced by the vaccinee since the last visit
- Record pre-vaccination body temperature
- Administer the third dose of RTS,S/AS01E or RTS,S/AS02D intramuscularly in the left deltoid

Each child will be assessed for at least 60 minutes after vaccination to evaluate and treat any acute adverse events.

- Record any post-vaccination solicited symptoms
- Record any post-vaccination unsolicited adverse events
- Record any post-vaccination SAEs
- Record concomitant medication
- Blood sample to collect 1 mL blood for analysis of:
  - hematology (complete blood count)
  - biochemistry (creatinine and ALT)
  - serology (antibodies to CS)

Field worker post vaccination follow-up visits 41, 42, 43, 44, 45, 46
Days 1, 2, 3, 4, 5 and 6 post Dose 3 of vaccine

- Check study identification card of vaccinee
- Record axillary temperature of subject

- Record local (pain and swelling at the injection site) and general (fever, irritability / fussiness, drowsiness, loss of appetite) solicited adverse events
- Record SAEs experienced by the vaccinee since the last visit
- Record unsolicited adverse events experienced by the vaccinee since the last visit
- Record concomitant medication

Clinic Visit 8
Month 8

- Check study identification card of vaccinee
- Carry out physical examination, take medical history, measure body weight, record vital signs
- Record axillary temperature of subject
- Record SAEs experienced by the vaccinee since the last visit
- Record unsolicited adverse events experienced by the vaccinee since the last visit
- Check elimination criteria
- Record concomitant medication
- Blood sample to collect 5 mL blood for analysis of:
  - hematology (complete blood count)
  - biochemistry (creatinine and ALT)
  - serology (antibodies to CS, HBs)
  - cell-mediated immunity

Field worker follow-up visit 47
Month 9

- Check study identification card of vaccinee
- Record axillary temperature of subject
- Record SAEs experienced by the vaccinee since the last visit
- Record concomitant medication

Clinic Visit 9
Month 10

- Check study identification card of vaccinee
- Carry out physical examination, take medical history, measure body weight, record vital signs
- Record axillary temperature of subject
- Record SAEs experienced by the vaccinee since the last visit

- Check elimination criteria
- Record concomitant medication
- Blood sample to collect 1 mL blood for analysis of:
  - hematology (complete blood count)
  - biochemistry (creatinine and ALT)
  - serology (antibodies to CS)

Field worker follow-up visit 48, 49, 50, 51, 52, 53, 54, 55
Months 11, 12, 13, 14, 15, 16, 17, 18

- Check study identification card of vaccinee
- Record axillary temperature of subject
- Record SAEs experienced by the vaccinee since the last visit
- Record concomitant medication

Clinic Visit 10
Month 19

- Check study identification card of vaccinee
- Carry out physical examination, take medical history, measure body weight, record vital signs
- Record axillary temperature of subject
- Record SAEs experienced by the vaccinee since the last visit
- Check elimination criteria
- Record concomitant medication
- Blood sample to collect 5 mL blood for analysis of:
  - hematology (complete blood count)
  - biochemistry (creatinine and ALT)
  - serology (antibodies to CS, HBs)
  - cell-mediated immunity

5.12. Sample handling and analysis

5.12.1. Treatment and storage of biological samples

See Appendix D of the protocol for details of treatment and storage of biological samples.

See Appendix E for instructions for shipment of biological samples.

5.12.2. Laboratory assays

Separation of serum from the blood samples and analysis of hematology and biochemistry will be carried out at the laboratories of the respective trial sites (the laboratory of KHRC and the laboratory of Agogo Presbyterian Hospital.

Table 14 Laboratory immunological assays to be performed

Assay	Marker	Assay method	Test kit/ Manufacturer	Assay unit	Assay cut-off	Laboratory
Anti-CS antibodies	R32LR	ELISA	In-house ELISA	EU/mL	0.5	Leroux-Roels Laboratory, CEVAC, Ghent, Belgium
Anti-HBs antibodies		EIA	AUSAB EIA ABBOTT*	mIU/mL	10**	GSK Biologicals†, Rixensart, Belgium
Cell-mediated immunity		ICS	In-house ICS	% cytokine positive cells		GSK Biologicals†, Rixensart, Belgium

*: or equivalent
**: seroprotective level
†: or designated validated laboratory
ELISA: Enzyme-linked Immunoabsorbent Assay
EIA: Enzyme immunoassay
CEVAC: Center for Vaccinology, Ghent University
ICS: Intra-cellular cytokine staining

The GSK Biologicals' laboratory at Rixensart has established Quality Control Procedures and an established Quality System. Both are audited regularly for quality assessment by an internal (sponsor-dependent) but laboratory-independent Quality Department to document the competency of the facility to perform the required tests and support the reliability of the results. Methods and equipment are validated, where required.

5.12.3. Serology plan

Serum for antibody determination will be collected by blood sample. Samples for safety will be analyzed at the time they are collected.

Serum samples from each child will be shipped to GSK Biologicals, Rixensart, Belgium.

Table 15 Summary of blood sampling time points/immunological assays

Blood sampling time point		Test	No. subjects	Laboratory	Priority ranking
Study month	Clinic Visit No.				
Day –28 to 0 **All Subjects**	1	Anti-CS antibodies	540	GSK Biologicals*	1
		Anti-HBs antibodies	540	GSK Biologicals*	2
Month 2 **Subjects on 0, 1 schedule**	5	Anti-CS antibodies	180	GSK Biologicals*	1
		Anti-HBs antibodies	180	GSK Biologicals*	2
		CMI	180	GSK Biologicals*	3
Month 3 **Subjects on 0, 1, 2 schedule**	6	Anti-CS antibodies	180	GSK Biologicals*	1
		Anti-HBs antibodies	180	GSK Biologicals*	2
		CMI	180	GSK Biologicals*	3
Month 7 **All Subjects**	7	Anti-CS antibodies	540	GSK Biologicals*	1
Month 8 **Subjects on 0, 1, 7 schedule**	8	Anti-CS antibodies	180	GSK Biologicals*	1
		Anti-HBs antibodies	180	GSK Biologicals*	2
		CMI	180	GSK Biologicals*	3
Month 10 **All Subjects**	9	Anti-CS antibodies	540	GSK Biologicals*	1
Month 19 **All subjects**	10	Anti-HBs antibodies	540	GSK Biologicals*	1
		Anti-CS antibodies	540	GSK Biologicals*	2
		CMI	540	GSK Biologicals*	3

*: or designated laboratory

6. INVESTIGATIONAL PRODUCTS AND ADMINISTRATION

6.1. Study vaccines

The candidate vaccines to be used have been developed and manufactured by GSK Biologicals.

The Quality Control Standards and Requirements for the candidate vaccine are described in separate release protocols and the required approvals have been obtained.

Commercial vaccines are assumed to comply with the specifications given in the manufacturer's Summary of Product Characteristics.

Refer to Appendix G for details of vaccine supplies.

6.1.1. RTS,S/AS01E vaccine

The final RTS,S/AS01E vaccine consists of two fractions: the lyophilized fraction containing the RTS,S antigen in one vial and the liquid fraction, consisting of AS01E adjuvant in the other vial to be used for reconstitution just prior to injection. A dose of 0.5 mL will be injected. The presentation of the reconstituted RTS,S/AS01E candidate malaria vaccine is an opalescent liquid.

6.1.1.1. RTS,S antigen presentation:

- The RTS,S antigen presents as a lyophilized pellet containing approximately 25 µg of antigen with sucrose as cryoprotectant per 3 mL monodose vial.

6.1.1.2. AS01E adjuvant:

- AS01E contains 25 µg of MPL, 25 µg of Stimulon® QS21 (a triterpene glycoside purified from the bark of *Quillaja saponaria*) with liposomes presented in a vial.

6.1.2. RTS,S/AS02D vaccine

The final RTS,S/AS02D vaccine consists of two fractions: the lyophilized fraction containing the RTS,S antigen in one vial and the liquid fraction, consisting of AS02D adjuvant in pre-filled syringes to be used for reconstitution just prior to injection. A dose of 0.5 mL will be injected. The presentation of the reconstituted RTS,S/AS02D candidate malaria is an opaque milky liquid.

6.1.2.1. RTS,S antigen presentation:

- The RTS,S antigen presents as a lyophilized pellet containing approximately 25 µg of antigen with sucrose as cryoprotectant per 3 mL monodose vial.

6.1.2.2. AS02D adjuvant:

- AS02D contains 25 µg of MPL, 25 µg of Stimulon® QS21 (a triterpene glycoside purified from the bark of *Quillaja saponaria*) and 125 µL of a proprietary oil-in-water emulsion and phosphate buffered saline per 0.5 mL, presented in prefilled syringes (PFS).

6.1.3. Rabipur Rabies vaccine

Rabipur®, is a sterile freeze-dried vaccine obtained by growing the fixed-virus strain Flury LEP in primary cultures of chicken fibroblasts.

The potency of one dose (1.0 mL) Rabipur is at least 2.5 IU of rabies antigen. Rabipur is a white, freeze-dried vaccine for reconstitution with the diluent prior to use; the reconstituted vaccine is a clear to slightly opaque, colorless suspension.

A dose of 1.0 mL will be injected. The presentation of the reconstituted vaccine is as a clear or slightly opaque suspension.

6.2. Dosage and administration

The vaccinees will be observed closely for at least 60 minutes following the administration of vaccines, with appropriate medical treatment readily available in case of an anaphylactic reaction.

6.2.1. RTS,S/AS01E

RTS,S/AS01E will be supplied such that the reconstituted vaccine volume will provide a 0.5 mL pediatric dose. One 0.5 mL dose will be withdrawn from each vial and used.

Disinfect top of vaccine vial (pellet) and adjuvant vial with alcohol swabs and let dry. Withdraw the contents of the adjuvant vial in a syringe and inject adjuvant into the vial of lyophilized antigen. Remove and discard the syringe and needle under appropriate safety precautions. The pellet is then dissolved by gently shaking the vial. Wait for 1 minute to ensure complete dissolution of vial contents before withdrawing a sufficient volume to provide a 0.5 mL dose (volume required for RTS,S/AS01E) of the reconstituted vaccine solution using a fresh needle and syringe for injection. The reconstituted vaccine should be administered by slow intramuscular (IM) injection, using a 25G needle with length of 1 inch (25 mm), in the left deltoid muscle within 4 hours of reconstitution (storage at +2°C to +8°C).

6.2.2. RTS,S/AS02D

RTS,S/AS02D will be supplied such that the reconstituted vaccine volume will provide a 0.5 mL pediatric dose. One 0.5 mL dose will be withdrawn from each vial and used.

Disinfect top of vaccine vial (pellet) with alcohol swabs and let dry. Inject complete contents of one PFS of adjuvant into vial of lyophilized vaccine. Remove and discard the syringe and needle under appropriate safety precautions. The pellet is then dissolved by gently shaking the vial. Wait for 1 minute to ensure complete dissolution of vial contents before withdrawing a sufficient volume to provide a 0.5 mL dose (volume required for RTS,S/AS02D) of the reconstituted vaccine solution using a fresh needle and syringe for injection. The reconstituted vaccine should be administered by slow IM injection, using a 25G needle with length of 1 inch (25 mm), in the left deltoid muscle within 4 hours of reconstitution (storage at 2°C to 8°C).

6.2.3. Rabipur Rabies vaccine

Disinfect top of vaccine vial with alcohol swabs and let dry. Inject the entire contents of the diluent ampoule into the vaccine vial. Keeping the syringe and needle in place, the freeze-dried vaccine is dissolved by gently shaking the vial. Wait for 1 minute to ensure complete dissolution of vial contents before withdrawing a sufficient volume to provide a 1.0 mL dose still using the original needle and original syringe. The original needle should then be replaced with a fresh needle for IM injection. The reconstituted vaccine should be used immediately.

6.3. Storage

ALL VACCINE VIALS/PRE-FILLED SYRINGES AND ADJUVANTS MUST BE STORED IN THE REFRIGERATOR (+2°C to +8°C) AND MUST NOT BE FROZEN.

All vaccines will be stored in a safe and locked place with no access for unauthorized personnel. Storage temperature will be monitored daily, according to SOPs at the investigator's site. An alarm system and a back-up refrigerator will be available in case of power failure/breakdown.

The study monitor must be contacted if the cold chain is broken (e.g. vaccines become frozen or refrigeration fails).

Storage conditions for transport of vaccines from country medical department or dispatch center to study sites or between sites are described in Appendix D.

6.4. Treatment allocation and randomization

6.4.1. Randomization of supplies

There will be a separate randomization list supplied to each site with no overlap of Treatment Numbers. The randomization list will be generated at GSK Biologicals, Rixensart, using a standard SAS (Statistical Analysis System) program and will be used to number the vaccines.

In this randomization process, volunteers will be allocated randomly to one of six study groups ***at each study site***, each defining which vaccine should be given ~~(i.e. RTS,S/AS01E, RTS,S/AS02D or Rabies vaccine)~~ and at which schedule (0, 1-month or; 0, 1, 2-month or 0, 1, 7-month). ***For the schedule 0, 1, 2-month at the Kintampo study site (KHRC), this means: RTS,S/AS01E or Rabies vaccine. For the same schedule at the Kumasi study site (KCCR/SMS), this means: RTS,S/AS01E or RTS,S/AS02D. For the other schedules at both study sites, this means either RTS,S/AS01E or RTS,S/AS02D. At each study site,*** all study groups will have the same number of subjects. Each investigator will be supplied with a list that indicates which Treatment Numbers correspond to which vaccination schedules. This procedure will mean that investigators will be blinded to vaccine administered to subjects, but not to the vaccination schedule. **Amended (04 July 2006)**

Subjects will receive Study ID cards with Subject Numbers at the screening visit (Clinic Visit 1). At first vaccination visit (Clinic Visit 2 [Month 0]), after verification of eligibility criteria, subjects will be allocated a Treatment Number in the order they present for vaccination with the lowest Treatment Number available from the randomization list. The correspondence between the Subject Number and the Treatment Number will be noted down in the Clinic Form and subsequently transferred to the Case Report Form.

6.4.2. Randomization of subjects

Subjects will be allocated sequentially to treatment numbers in the order that they present at each study center for vaccination.

The actual treatment number used for first vaccination of the subject must be recorded by the investigator in the CRF (Rando/Treatment Allocation Section).

6.5. Method of blinding and breaking the study blind

Data pertaining to RTS,S/AS02D, RTS,S/AS01E or Rabies vaccine will be collected in a partially blinded manner. 'Partially Blinded' means that the vaccine recipient and their parent(s)/guardian(s) as well as those responsible for the evaluation of safety and immunogenicity endpoints will all be unaware which treatment, RTS,S/AS02D, RTS,S/AS01E or Rabies vaccine, was administered to a particular subject. However, because parents/guardians/study staff are aware of what vaccination schedule is being given to each volunteer, the study is 'open' in terms of schedule.

Code break envelopes, for each study enrolled subject and associating each treatment number with a specific vaccine, will be kept by the Local Safety Monitors at each site as well as by Central Safety at GSK Biologicals, Rixensart in a safe and locked place with no access for unauthorized personnel.

If deemed necessary for reasons such as safety, the Local Safety Monitor(s) as well as GSK Biologicals Central Safety will unblind the specific enrolled subject without revealing the study blind to the investigators.

As part of the safety monitoring plan, safety reports will be produced at 3 timepoints in the trial (refer to 5.1.3.3). An independent statistician will analyze the data and provide an unblinded report to the DSMB, thereby maintaining the blind of the malaria project team at GSK Biologicals and the investigator group.

A formal reporting and analysis plan (RAP) will be developed. Once the study is completed and the GSK Biologicals reference database locked, GSK Biologicals will be responsible for initiating the execution of the statistical analysis plan.

GSK Biologicals' policy (incorporating ICH E2A guidance, EU Clinical Trial Directive and Federal Regulations) is to unblind any serious adverse event (SAE) report associated with the use of the investigational product, which is unexpected and attributable/suspected, prior to regulatory reporting. The Clinical Safety Physician at GSK Biologicals is responsible for unblinding the treatment assignment in accordance with specified time frames for expedited reporting of SAEs (Refer to Section 8.8).

6.6. Replacement of unusable vaccine doses

Additional vaccine doses will be provided to replace those that are unusable (see Appendix G for details of supplies).

In addition to the vaccine doses provided for the planned number of enrolled subjects, 3% additional doses will be supplied. In case a vaccine dose is broken or unusable, the investigator should replace it with a replacement vaccine dose. If a vaccine dose needs replacement, the envelope with the corresponding treatment number will designate the replacement without unblinding the study using a coded letter system. Although the sponsor need not be notified immediately in these cases, documentation of the use of the replacement vaccine must be recorded by the investigator on the vaccine administration page of the CRF and on the vaccine accountability form.

6.7. Packaging

See Appendix G.

6.8. Vaccine accountability

See Appendix G.

6.9. Concomitant medication/treatment

At each study visit/contact, the investigator should question the enrolled subject's parent(s)/guardian(s) about any medication(s) taken.

All antipyretic, analgesic and antibiotic drugs, administered at ANY time during the period starting with administration of each dose and ending 30 days after each dose of vaccine are to be recorded with generic name of the medication (trade names are allowed for combination drugs, i.e., multi-component drugs), medical indication, total daily dose, route of administration, start and end dates of treatment.

Any treatments and/or medications which are listed as elimination criteria in Section 4.5, e.g., any immunoglobulins, other blood products and any immune modifying drugs administered within three months preceding the first dose or at any time during the study period are to be recorded with generic name of the medication (trade names are allowed for combination drugs only), medical indication, total daily dose, route of administration, start and end dates of treatment. Refer to Sections 4.3 to 4.6. The time periods between which each type of concomitant medication/treatment should be recorded is summarized in Table 16.

Table 16 Summary of time periods between which different classes of concomitant medication/treatment/vaccination must be recorded

**3 months prior to Dose 1 → Dose 1**	All treatments listed as elimination criteria in Section 4.5*
Dose 1 ~~**Screening**~~ **→ *Study month 10*** ~~**30 Days post Dose 3**~~	All antipyretic, analgesic, antibiotic and any treatments listed as elimination criteria in Section 4.5*<br>All vaccinations
1 Day post Study month 10 ~~**31 Days post Dose 3**~~ **→ Final Study Visit**	All treatments listed as elimination criteria in Section 4.5*

* e.g. any immunoglobulins, other blood products and any immune modifying drugs

Amended (04 July 2006)

Any vaccine not foreseen in the study protocol administered in the period beginning 30 days preceding each dose and ending 30 days after each dose is to be recorded with trade name, route of administration and date(s) of administration. Refer to Sections 4.3 to 4.6 and 5.8.2.

Any concomitant medication administered prophylactically in anticipation of reaction to the vaccination must be recorded in the CRF with generic name of the medication (trade

names are allowed for combination drugs only), total daily dose, route of administration, start and end dates of treatment and coded as 'Prophylactic'.

7. HEALTH ECONOMICS

Not Applicable

8. ADVERSE EVENTS AND SERIOUS ADVERSE EVENTS

The investigators are responsible for the detection and documentation of events meeting the criteria and definition of an adverse event (AE) or serious adverse event (SAE) as provided in this protocol. During the study, when there is a safety evaluation, the investigators or site staff will be responsible for detecting AEs and SAEs, as detailed in this section of the protocol.

Each subject's parents/guardians will be instructed to contact the investigator immediately should the subject manifest any signs or symptoms they perceive as serious.

8.1. Definition of an adverse event

An AE is any untoward medical occurrence in a clinical investigation subject, temporally associated with the use of a medicinal product, whether or not considered related to the medicinal product.

An AE can therefore be any unfavorable and unintended sign (including an abnormal laboratory finding), symptom, or disease (new or exacerbated) temporally associated with the use of a medicinal product.

Examples of an AE include:

- Exacerbation of a chronic or intermittent pre-existing condition including either an increase in frequency and/or intensity of the condition.
- New conditions detected or diagnosed after investigational product administration even though it may have been present prior to the start of the study.
- Signs, symptoms, or the clinical sequelae of a suspected overdose of either investigational product or a concurrent medication (overdose per se should not be reported as an AE/SAE).
- Signs, symptoms temporally associated with vaccine administration.

AEs may include pre-or post-treatment events that occur as a result of protocol-mandated procedures (i.e., invasive procedures, modification of subject's previous therapeutic regimen).

N.B. AEs to be recorded as endpoints (solicited events) are described in Section 8.4.1. All other AEs will be recorded as **UNSOLICITED AEs**.

Example of events to be recorded in the medical history section of the CRF:

- Pre-existing conditions or signs and/or symptoms present in a subject prior to the start of the study (i.e. prior to the first study procedure) should be recorded in the medical history section of the subject's CRF.

8.2. Definition of a serious adverse event

A serious adverse event (SAE) is any untoward medical occurrence that:

a. results in death;

b. is life-threatening;

NOTE: The term 'life-threatening' in the definition of 'serious' refers to an event in which the subject was at risk of death at the time of the event. It does not refer to an event, which hypothetically might have caused death, if it were more severe.

c. requires hospitalization or prolongation of existing hospitalization;

NOTE: In general, hospitalization signifies that the subject has been detained (usually involving at least an overnight stay) at the hospital or emergency ward for observation and/or treatment that would not have been appropriate in the physician's office or out-patient setting. Complications that occur during hospitalization are AEs. If a complication prolongs hospitalization or fulfills any other serious criteria, the event is serious. When in doubt as to whether 'hospitalization' occurred or was necessary, the AE should be considered serious.

Hospitalization for elective treatment of a pre-existing condition that did not worsen from baseline is not considered an AE.

d. results in disability/incapacity;

NOTE: The term disability means a substantial disruption of a person's ability to conduct normal life functions. This definition is not intended to include experiences of relatively minor medical significance such as uncomplicated headache, nausea, vomiting, diarrhea, influenza, and accidental trauma (e.g. sprained ankle) which may interfere or prevent everyday life functions but do not constitute a substantial disruption.

e. medical or scientific judgment should be exercised in deciding whether reporting is appropriate in other situations, such as important medical events that may not be immediately life-threatening or result in death or hospitalization but may jeopardize the subject or may require medical or surgical intervention to prevent one of the other outcomes listed in the above definition. These should also be considered serious. Examples of such events are invasive or malignant cancers, intensive treatment in an emergency room or at home for allergic bronchospasm, blood dyscrasias or convulsions that do not result in hospitalization.

In this study all seizures occurring within a 30-day period of vaccination will be notified as SAEs [refer to Bonhoeffer 2004]. Key information pertaining to seizures will be documented in the CRF.

8.3. Clinical laboratory parameters and other abnormal assessments qualifying as adverse events and serious adverse events

Abnormal laboratory findings (e.g. clinical chemistry, hematology, urinalysis) or other abnormal assessments (e.g. blood film) that are judged by the investigator to be clinically significant will be recorded as AEs or SAEs if they meet the definition of an AE, as defined in Section 8.1 or SAE, as defined in Section 8.2. Clinically significant abnormal laboratory findings or other abnormal assessments that are detected during the study or are present at baseline and significantly worsen following the start of the study will be reported as AEs or SAEs.

The investigator will exercise their medical and scientific judgment in deciding whether an abnormal laboratory finding or other abnormal assessment is clinically significant.

8.4. Time period, frequency, and method of detecting adverse events and serious adverse events

All AEs occurring within 30 days following administration of each dose of vaccine must be recorded on the Adverse Event form in the subject's CRF, irrespective of severity or whether or not they are considered vaccination-related.

The standard time period for collecting and recording SAEs will begin at first receipt of vaccine and will end at the study conclusion. See Section 8.7 for instructions for reporting and recording SAEs.

Additionally, in order to fulfill international reporting obligations, SAEs that are related to study participation (e.g. procedures, invasive tests, a change from existing therapy) or are related to a concurrent medication will be collected and recorded from the time the subject consents to participate in the study until she/he is discharged: if a child is screened and enrolled, then discharge will be the end of the study as per study protocol; if a child is screened but not enrolled, then discharge will be the point at which the decision is taken not to enroll the child.

The investigator will inquire about the occurrence of AEs/SAEs at every visit/contact during the study and throughout the follow-up phase as appropriate.

The mechanism by which SAEs will be identified in the study are detailed in Section 5.7. The investigator or study clinician will fully document any such events on the Serious Adverse Event pages appended to the individual Case Report Form including, where applicable, information from relevant hospital case records, autopsy reports and verbal autopsies.

All AEs either observed by the investigator, study clinician, field worker or reported by the subject's parent/guardian spontaneously or in response to a direct question will be evaluated by the investigator. AEs not previously documented in the study will be recorded in the Adverse Event form within the subject's CRF. The nature of each event, date and time (where appropriate) of onset, outcome, intensity and relationship to

vaccination will be established. Details of any corrective treatment will be recorded on the appropriate page of the CRF. Refer to Section 6.9.

As a consistent method of determining the occurrence of unsolicited AEs, the subject or the subject's parent/guardian will be asked a non-leading question such as:

'Has your child acted differently or felt different in any way since receiving the vaccine or since the last visit '

N.B. The investigator should record only those AEs having occurred within the time frame defined above.

AEs already documented in the CRF, i.e. at a previous assessment, and designated as 'not recovered/not resolved' or 'recovering/resolving' should be reviewed at subsequent visits, as necessary. If these have resolved, the documentation in the CRF should be completed.

When an AE/SAE occurs, it is the responsibility of the investigator to review all documentation (e.g., hospital progress notes, laboratory, verbal autopsies and diagnostics reports) relative to the event. The investigator will then record all relevant information regarding an AE/SAE on the CRF or SAE Report Form as applicable. It is not acceptable for the investigator to send photocopies of the subject's medical records to GSK Biologicals in lieu of the appropriate completed AE/SAE pages. However, there may be instances when copies of medical records and verbal autopsies for certain cases are requested by GSK Biologicals. In this instance, all subject identifiers will be blinded on the copies of the medical records prior to submission to GSK Biologicals.

The investigator will attempt to establish a diagnosis of the event based on signs, symptoms, and/or other clinical information. In such cases, the diagnosis should be documented as the AE/SAE and not the individual signs/symptoms.

8.4.1. Solicited adverse events

Local (injection site) adverse events

- Pain at injection site
- Swelling at injection site

General adverse events

- Fever (defined as axillary temperature ≥ 37.5°C)
- Drowsiness
- Loss of appetite
- Irritability/fussiness

The visiting field worker will record these adverse events according to detailed SOPs available at the study sites during the field worker visits.

N.B. Temperature will be recorded on days 1 to 6 following each vaccination by the field worker or Principal Investigator (or their delegate). Should additional temperature measurements be performed at other times of day, the highest temperature will be recorded.

8.5. Evaluating adverse events and serious adverse events

8.5.1. Assessment of intensity

Intensity of the following AEs will be assessed as described:

Table 17 Intensity scales for solicited symptoms in infants/toddlers and children less than 6 years of age

Adverse Event	Intensity grade	Parameter
Pain at injection site	0	Absent
	1	Minor reaction to touch
	2	Cries/protests on touch
	3	Cries when limb is moved/spontaneously painful
Swelling at injection site		Record greatest surface diameter in mm
Fever*		Record temperature in °C
Irritability/Fussiness	0	Behavior as usual
	1	Crying more than usual/ no effect on normal activity
	2	Crying more than usual/ interferes with normal activity
	3	Crying that cannot be comforted/ prevents normal activity
Drowsiness	0	Behavior as usual
	1	Drowsiness easily tolerated
	2	Drowsiness that interferes with normal activity
	3	Drowsiness that prevents normal activity
Loss of appetite	0	Appetite as usual
	1	Eating less than usual/ no effect on normal activity
	2	Eating less than usual/ interferes with normal activity
	3	Not eating at all

*Fever is defined as axillary temperature ≥ 37.5°C

The maximum intensity of local injection site swelling will be scored at GSK Biologicals as follows:

0 : None
1 : < 5 mm
2 : 5 to 20 mm
3 : > 20 mm

The maximum intensity of fever will be scored at GSK Biologicals as follows:

0 : < 37.5°C
1 : 37.5 – 38.0°C
2 : > 38 – 39.0°C
3 : > 39.0°C

Table 18 Acceptable/normal ranges for blood testing

	Acceptable limit/ normal range	Toxicity grading scale			
		Grade 1	Grade 2	Grade 3	Grade 4
Hemoglobin	≥ 8.0 g/dL	< ***8.0 g/dL*** ~~ULN~~	< 6.0 g/dL	< 5.0 g/dL	< 5.0 g/dL & clinical signs of heart failure
Total white cell count†	≥ 4.0 x 10^3/µL<br>< 17 x 10^3/µL	2.5 to 4.0 x 10^3/µL	1.5 to 2.4 x 10^3/µL	1.0 to 1.4 x 10^3/µL	< 1.0 x 10^3/µL
Platelets†	≥ 100 x 10^3/µL	50 to 99 x 10^3/µL	25 to 49 x 10^3/µL	< 25 x 10^3/µL	< 25 x 10^3/µL & clinical signs of bleeding
ALT*	≤ 60 ***IU/L***~~µmol/L~~	1.1 to 2.5 x ULN	2.6 to 5.0 x ULN	5.1 to 10.0 x ULN	> 10.0 x ULN
Creatinine*	≤ 60 µmol/L (or 0.6 mg/dL)	1.1 to 1.5 x ULN	1.6 to 3.0 x ULN	3.1 to 6.0 x ULN	> 6.0 ULN or requires dialysis

†: Grading scale adapted from Division of AIDS table for grading severity of adult and pediatric adverse events December 2004
*: Grading scale adapted from WHO Toxicity Grading Scale for Determining Severity of Adverse Events, February 2003.
ULN: Upper Limit of Normal LLN: Lower Limit of Normal

Amended (04 July 2006)

The investigator will make an assessment of the maximum intensity that occurred over the duration of the event for all other AEs, i.e. unsolicited symptoms, including SAEs reported during the study. The assessment will be based on the investigator's clinical judgment. The intensity of each AE and SAE recorded in the CRF or SAE Report Form, as applicable, should be assigned to one of the following categories:

1 (mild) = An AE which is easily tolerated by the subject, causing minimal discomfort and not interfering with everyday activities.

2 (moderate) = An AE which is sufficiently discomforting to interfere with normal everyday activities.

3 (severe) = An AE which prevents normal, everyday activities. (In a young child, such an AE would, for example, prevent attendance at school/ kindergarten/ a day-care centre and would cause the parents/ guardians to seek medical advice. In adults/ adolescents, such an AE would, for example, prevent attendance at work/ school and would necessitate the administration of corrective therapy.)

An AE that is assessed as Grade 3 (severe) should not be confused with a SAE. Grade 3 is a category utilized for rating the intensity of an event; and both AEs and SAEs can be assessed as Grade 3. An event is defined as 'serious' when it meets one of the pre-defined outcomes as described in Section 8.2.

8.5.2. Assessment of causality

The investigator is obligated to assess the relationship between investigational product and the occurrence of each AE/SAE. The investigator will use clinical judgment to determine the relationship. Alternative causes, such as natural history of the underlying diseases, concomitant therapy, other risk factors and the temporal relationship of the event to the investigational product will be considered and investigated. The investigator will also consult the Investigator Brochure and/or Product Information, for marketed products, in the determination of his/her assessment.

There may be situations when a SAE has occurred and the investigator has minimal information to include in the initial report to GSK Biologicals. However, it is very important that the investigator always makes an assessment of causality for every event prior to transmission of the SAE Report Form to GSK Biologicals. The investigator may change his/her opinion of causality in light of follow-up information, amending the SAE Report Form accordingly. The causality assessment is one of the criteria used when determining regulatory reporting requirements.

In case of concomitant administration of multiple vaccines, it may not be possible to determine the causal relationship of general AEs to the individual vaccines administered. The investigator should, therefore, assess whether the AE could be causally related to vaccination rather than to the individual vaccines.

All solicited local (injection site) reactions will be considered causally related to vaccination. Causality of all other AEs should be assessed by the investigator using the following question:

Is there a reasonable possibility that the AE may have been caused by the investigational product ?

NO : The AE is not causally related to administration of the study vaccine(s). There are other, more likely causes and administration of the study vaccine(s) is not suspected to have contributed to the AE.

YES : There is a reasonable possibility that the vaccine(s) contributed to the AE.

Non-serious and serious AEs will be evaluated as two distinct events. If an event meets the criteria to be determined “serious” (see Section 8.2 for definition of serious adverse event), it will be examined by the investigator to the extent to be able to determine ALL contributing factors applicable to each serious adverse event.

Other possible contributors include:

- Medical history
- Other medication
- Protocol required procedure

- Other procedure not required by the protocol
- Lack of efficacy of the vaccine(s), if applicable
- Erroneous administration
- Other cause (specify).

8.6. Follow-up of adverse events and serious adverse events and assessment of outcome

After the initial AE/SAE report, the investigator is required to proactively follow each subject and provide further information to GSK Biologicals on the subject's condition.

All AEs and SAEs documented at a previous visit/contact and designated as not recovered/not resolved or recovering/resolving will be reviewed at subsequent visits/contacts.

Investigators will follow-up subjects:

- with SAEs or subjects withdrawn from the study as a result of an AE, until the event has resolved, subsided, stabilized, disappeared, the event is otherwise explained, or the subject is lost to follow-up;
- or, in the case of other non-serious AEs, until they complete the study or they are lost to follow-up.

All Grade 3, Grade 4 or clinically significant laboratory abnormalities will be followed up until they have returned to normal, or a satisfactory explanation has been provided. Additional information (including but not limited to laboratory results) relative to the subsequent course of such an abnormality noted for any subject must be made available to the Study Monitor.

GSK Biologicals may request that the investigator perform or arrange for the conduct of supplemental measurements and/or evaluations to elucidate as fully as possible the nature and/or causality of the AE or SAE. The investigator is obliged to assist. If a subject dies during participation in the study or during a recognized follow-up period, GSK Biologicals will be provided with a copy of any available post-mortem findings, including histopathology.

New or updated information will be recorded on the originally completed SAE Report Form, with all changes signed and dated by the investigator. The updated SAE report form should be resent to GSK Biologicals within 24 hours of receipt of the follow-up information as outlined in Section 8.7.1.

Outcome of any non-serious AE occurring within 30 days post-vaccination (i.e. unsolicited AE) or any SAE reported during the entire study will be assessed as:

- Recovered/resolved
- Not recovered/not resolved

- Recovering/resolving
- Recovered with sequelae/resolved with sequelae
- Fatal (SAEs only).

8.7. Prompt reporting of serious adverse events to GSK Biologicals

8.7.1. Time frames for submitting serious adverse event reports to GSK Biologicals

SAEs will be reported promptly to GSK once the investigator determines that the event meets the protocol definition of an SAE. The investigator or designate will fax the SAE reports to GSK Biologicals' Study Contact for Serious Adverse Event Reporting **WITHIN 24 HOURS OF HIS BECOMING AWARE OF THESE EVENTS**. Additional or follow-up information relating to the initial SAE report is also to be reported to the GSK Biologicals' Study Contact for Serious Adverse Event Reporting within 24 hours of receipt of such information.

8.7.2. Completion and transmission of serious adverse event reports to GSK Biologicals

Once an investigator becomes aware that a SAE has occurred in a study subject, she/he will report the information to GSK within 24 hours as outlined in Section 8.7.1. The SAE Report Form will always be completed as thoroughly as possible with all available details of the event, signed by the investigator (or designee), and forwarded to GSK within the designated time frames. If the investigator does not have all information regarding an SAE, he/she will not wait to receive additional information before notifying GSK of the event and completing the form. The form will be updated when additional information is received and forwarded to GSK **WITHIN 24 HOURS** as outlined in Section 8.7.1.

The investigator will always provide an assessment of causality at the time of the initial report as described in Section 8.5.2.

Facsimile (Fax) or electronic transmission of the SAE Report Form are the preferred methods to transmit this information to the Study Contact for Reporting SAEs. In rare circumstances and in the absence of facsimile equipment or electronic connection, notification by telephone is acceptable, with a copy of the SAE Report Form to follow. Initial notification via the telephone does not replace the need for the investigator to complete and sign the SAE Report Form within 24 hours as outlined in Section 8.7.1.

In the event of a death determined by the investigator to be related to vaccination, sending of the fax must be accompanied by telephone call to the Study Contact for Reporting SAEs.

Study Contacts for Reporting of a Serious Adverse Event occurring at KCCR/SMS

All four of the contacts listed below must be informed of each SAE occurring at KCCR/SMS

Central Study Coordinator (KCCR/SMS)

Arlette Simonon
Central Study Coordinator,
GlaxoSmithKline Biologicals,
Rue de l'Institut 89, 1330 Rixensart,
Belgium.

Tel: +32.2.656.55.07
Fax: +32.2.656.80.44
email:arlette.simonon@gskbio.com

Local Safety Monitor (KCCR/SMS)

Gyikua Plange-Rhule,
Department of Child Health,
KATH/SMS,
Kumasi, Ghana.

Tel: +233.20.813.77.80
email: gyikua@hotmail.com

Malaria Vaccine Initiative

Carolyn Petersen,
Malaria Vaccine Initiative, PATH,
7500 Old Georgetown Road, 12th Floor,
Bethesda, MD 20814,
USA.

Tel: +1.240.395.27.16
or Tel: +1.240.395.27.00
Fax: +1.240.395.25.91
email: sae@malariavaccine.org

alternate email:
cpetersen@malariavaccine.org

Study Contact at GSK Biologicals for Reporting Serious Adverse Events Manager Clinical Safety Vaccines

GSK Biologicals Clinical Safety Physician,
GlaxoSmithKline Biologicals,
Rue de l'Institut 89, 1330 Rixensart,
Belgium.

Tel: +32.2.656.87.98
Fax: +32.2.656.80.09
Mobile phone for 7/7 day availability: +32.477.40.47.13
email: rix.ct-safety-vac@gskbio.com

Study Contacts for Reporting of a Serious Adverse Event occurring at KHRC

All four of the contacts listed below must be informed of each SAE occurring at KHRC

Central Study Coordinator (KHRC)	Local Safety Monitor (KHRC)
Nathalie Annez<br>Central Study Coordinator,<br>GlaxoSmithKline Biologicals,<br>Rue de l'Institut 89, 1330 Rixensart,<br>Belgium.<br><br>Tel: +32.2.656.57.48<br>Fax: +32.2.656.80.44<br>email: nathalie.annez@gskbio.com	Sam Newton<br>Clinical Research Fellow<br>Kintampo Health Research Centre,<br>Kintampo, Ghana<br><br>Tel: +233.61.28.869<br>sam.newton@ghana-khrc.org
**Malaria Vaccine Initiative**	**Study Contact at GSK Biologicals for Reporting Serious Adverse Events Manager Clinical Safety Vaccines**
Carolyn Petersen,<br>Malaria Vaccine Initiative, PATH,<br>7500 Old Georgetown Road, 12th Floor,<br>Bethesda, MD 20814,<br>USA.<br><br>Tel: +1.240.395.27.16<br>*or* Tel: +1.240.395.27.00<br>Fax: +1.240.395.25.91<br>email: sae@malariavaccine.org<br><br>*alternate email:*<br>cpetersen@malariavaccine.org	GSK Biologicals Clinical Safety Physician,<br>GlaxoSmithKline Biologicals,<br>Rue de l'Institut 89, 1330 Rixensart,<br>Belgium.<br><br>Tel: +32.2.656.87.98<br>Fax: +32.2.656.80.09<br>Mobile phone for 7/7 day availability:<br>+32.477.40.47.13<br>email: rix.ct-safety-vac@gskbio.com

8.8. Regulatory reporting requirements for serious adverse events

The investigator will promptly report all SAEs to GSK in accordance with the procedures detailed in Section 8.7. GSK Biologicals has a legal responsibility to promptly notify, as appropriate, both the local regulatory authority and other regulatory agencies about the safety of a product under clinical investigation. Prompt notification of SAEs by the investigator to the Study Contact for Reporting SAEs is essential so that legal obligations and ethical responsibilities towards the safety of other subjects are met.

The investigator, or responsible person according to local requirements, will comply with the applicable local regulatory requirements related to the reporting of SAEs to the IRB/IEC and, if required, to the applicable government authority.

Investigator safety reports are prepared according to the current GSK policy and are forwarded to investigators as necessary. An investigator safety report is prepared for a SAE(s) that is both attributable to investigational product and unexpected. The purpose of the report is to fulfill specific regulatory and Good Clinical Practice (GCP) requirements, regarding the product under investigation.

An investigator who receives an investigator safety report describing a SAE(s) or other specific safety information (e.g., summary or listing of SAEs) from GSK Biologicals will file it with the Investigator Brochure or other appropriate study documentation and will notify the IRB or IEC, if appropriate according to local requirements.

8.9. Post-study adverse events and serious adverse events

A post-study AE/SAE is defined as any event that occurs outside of the AE/SAE detection period defined in Section 8.4. Investigators are not obligated to actively seek AEs or SAEs in former study participants.

However, if the investigator learns of any SAE, including a death, at any time after a subject has been discharged from the study, and he/she considers the event reasonably related to the investigational product, the investigator will promptly notify the Study Contact for Reporting SAEs.

8.10. Pregnancy

Not applicable

8.11. Treatment of adverse events

Treatment of any adverse event is at the sole discretion of the investigator and according to current good medical practice. Any medication administered for the treatment of an AE should be recorded in the subject's CRF. Refer to Section 6.9.

9. SUBJECT COMPLETION AND WITHDRAWAL

9.1. Subject completion

A subject who returns for the concluding visit/is available for the concluding contact foreseen in the protocol is considered to have completed the study.

A subject that comes for Clinic Visit 9 has completed the primary study phase. A subject that comes for Clinic Visit ***10*** ~~11~~ has completed the extended follow-up phase of the study. **Amended (04 July 2006)**

9.2. Subject withdrawal

Subjects who are withdrawn because of AEs must be clearly distinguished from subjects who are withdrawn for other reasons. Investigators will follow subjects who are withdrawn as result of a SAE/AE until resolution of the event (see Section 8.6). Withdrawals will not be replaced.

9.2.1. Subject withdrawal from the study

From an analysis perspective, a 'withdrawal' from the study is any subject who was not available for the concluding contact foreseen in the protocol. A subject that comes for Clinic Visit 9 has completed the primary study phase. A subject that comes for Clinic Visit ***10*** ~~11~~ has completed the extended follow-up phase of the study. **Amended (04 July 2006)**

A subject qualifies as a 'withdrawal' from the study when no study procedure has occurred, no follow-up has been performed and no further information has been collected for this subject from the date of withdrawal/last contact.

Investigators will make an attempt to contact those subjects who do not return for scheduled visits or follow-up.

Information relative to the withdrawal will be documented on the Study Conclusion page of the CRF. The investigator will document whether the decision to withdraw from the study was made by the subject's parent or guardian or the investigator and which of the following possible reasons was responsible for withdrawal:

- serious adverse event
- non-serious adverse event
- protocol violation (specify)
- consent withdrawal, not due to an adverse event
- moved from the study area
- lost to follow-up
- death
- other (specify).

9.2.2. Subject withdrawal from investigational product

A 'withdrawal' from the investigational product is any subject who does not receive the complete treatment, i.e. when no further planned dose is administered from the date of withdrawal. A subject withdrawn from the investigational product may not necessarily be withdrawn from the study as further study procedures or follow-up may be performed (safety or immunogenicity) if planned in the study protocol.

Information relative to premature discontinuation of the investigational product will be documented on the Vaccine Administration page of the CRF. The investigator will document whether the decision to discontinue further vaccination/treatment was made by the subject's parent or guardian or the investigator and which of the following possible reasons was responsible for withdrawal:

- serious adverse event;
- non-serious adverse event;
- other (specify).

10. DATA EVALUATION: CRITERIA FOR EVALUATION OF OBJECTIVES

10.1. Primary endpoint

Safety

- Occurrence of SAEs from the time of first vaccination until ten months post Dose 1.

10.2. Secondary endpoints

Safety & Reactogenicity

- Occurrence of solicited general and local reactions over a 7-day follow-up period (day of vaccination and 6 subsequent days) after each vaccination.
- Occurrence of unsolicited symptoms after each vaccination over a 30-day follow-up period (day of vaccination and 29 subsequent days).

Immunogenicity

- Anti-CS antibody titers prior to vaccination until ten months post Dose 1.
- Anti-HBs antibody titers prior to vaccination and one month post final vaccine dose

10.3. Tertiary endpoints

Safety

- Occurrence of SAEs from ten to 19 months post Dose 1.

Immunogenicity

- Anti-HBs antibody titers at 19 months post Dose 1.
- Anti-CS antibody titers at 19 months post Dose 1.

10.4. Exploratory Endpoints

Safety

- Occurrence of SAEs from ten to 19 months post Dose 1 when vaccinees are stratified by age at the time of Dose 1.
- Occurrence of solicited general and local reactions over a 7-day follow-up period (day of vaccination and 6 subsequent days) after each vaccination when vaccinees stratified by age at the time of Dose 1.
- Occurrence of unsolicited symptoms after each vaccination over a 30-day follow-up period (day of vaccination and 29 subsequent days) when vaccinees stratified by age at the time of Dose 1.

Immunogenicity

- Anti-HBs antibody titers until 19 months post Dose 1 when vaccinees stratified by age at the time of Dose 1.
- Anti-CS antibody titers until 19 months post Dose 1 when vaccinees stratified by age at the time of Dose 1.

Cell-mediated immunity

- Frequency of CS-specific T-cells one month post final dose and at Month 19.

10.5. Estimated sample size

10.5.1. Sample size for the primary safety endpoint

Safety analyses will be performed on the Total Vaccinated Cohort. A trial of this size has the power to detect only large differences in the frequencies of AEs with reasonable power. A sample size of ***540*** ~~75 evaluable~~ subjects ~~per group~~ has the power to detect differences in the rates of safety endpoints between groups as shown in Table 19. Comparisons between groups will be done using Fisher's Exact test for each AE coded by preferred term (alpha = 0.05, 2 sided). **Amended (04 July 2006)**

Table 19 Differences in safety endpoints (between treatment groups that can be detected with 80% power for varying baseline rates)

~~Frequency of events for *x**~~	~~Frequency of events for *y**~~	~~Power to detect difference (number per group equals 90 75)~~
~~1%~~	~~13%~~	~~80%~~
~~3%~~	~~18%~~	~~80%~~
~~5%~~	~~21%~~	~~80%~~
~~10%~~	~~29%~~	~~80%~~

~~* where *x* and *y* are the study groups to be compared~~

Amended (04 July 2006)

Frequency of events for *x**	Frequency of events for *y** *N1=N2=90*	Frequency of events for *y** *N1=90, N2=45*
1%	12%	13%
3%	16%	18%
5%	20%	22%
10%	27%	31%

* where *x* and *y* are the study groups to be compared
80% Power, 2-sided Fisher's exact test, alpha=0.05

Amended (04 July 2006)

10.5.2. Sample size for the secondary immunogenicity endpoint

Anti-CS titers may be compared between study groups at one month post last dose and ten months post Dose 1. For any comparison between any two study groups a sample size of 75 evaluable subjects per group will have 90% power to demonstrate equivalence of the two vaccine regimens under comparison (i.e. *x* versus *y*) in terms of immune response (95% CI of the GMT ratio *x*/*y* is within the range 0.33 to 3.0) assuming a log standard deviation of 0.9 in both groups, alpha=0.025 (Pass 2000).

10.6. Study cohorts to be evaluated

10.6.1. Total Vaccinated Cohort

The Total Vaccinated Cohort will include all vaccinated subjects for whom data are available. Thus, the total analysis of safety will include all subjects with at least one vaccine administration documented and the total analysis of immunogenicity will include vaccinated subjects for whom data concerning immunogenicity endpoint measures are available. The Total Vaccinated Cohort analysis will be performed per treatment actually administered.

10.6.2. According to protocol (ATP) cohort for analysis of safety

The ATP cohort for analysis of safety will include all evaluable subjects;

- who have received at least one dose of study vaccine according to their random assignment
- have sufficient data to perform an analysis of safety (at least one vaccine dose with safety follow-up)
- for whom administration site of study vaccine is per protocol
- who have not received a vaccine not specified or forbidden in the protocol and for whom elimination criteria were not applied
- for whom the randomization code has not been broken
- who meet all eligibility criteria.

10.6.3. According to protocol (ATP) cohort for analysis of immunogenicity

The ATP cohort for analysis of immunogenicity will include all evaluable subjects (i.e. those meeting all eligibility criteria, complying with the procedures defined in the protocol, with no elimination criteria during the study) for whom data concerning immunogenicity endpoint measures are available.

10.7. Derived and transformed data

- Seroprotection rate for anti-HBsAg is defined as the percentage of subjects with antibody titers greater than or equal to an established cut-off (anti-HBsAg ≥ 10 mIU/mL).
- A subject seropositive for anti-CS antibody is a subject whose antibody titer is greater than or equal to the cut-off value (anti-CS ≥ 0.5 EU/mL).
- The Geometric Mean Titers (GMTs) calculations are performed by taking the anti-log of the mean of the log10 titer transformations. Antibody titers below the cut-off of the assay will be given an arbitrary value of half the cut-off for the purpose of GMT calculation.

For a given subject and a given immunogenicity measurement, missing or non-evaluable measurements will not be replaced. Therefore, an analysis will exclude subjects with missing or non-evaluable measurements.

10.8. Final analyses

The final analysis will be conducted on data collected until Clinic Visit 9, Month 10 (10 months post Dose 1 of vaccines). The study will continue in a single blind manner for additional safety surveillance and immunogenicity assessment. This additional information will be appended to the study report.

10.8.1. Analysis of demographics/baseline characteristics

Demographic characteristics (age, gender) of each study cohort will be tabulated.

The mean age (plus range and standard deviation) by gender of the enrolled subjects, as a whole, and per group, will be calculated.

The distribution of subjects enrolled by study site will be tabulated as a whole and per group.

10.8.2. Analyses of safety

10.8.2.1. Primary endpoint

Analysis of the primary endpoint, the occurrence of SAEs will be performed on the Total Vaccinated Cohort. The proportion of subjects with an SAE, classified by the MedDRA preferred term level, reported from study start until study conclusion (end of the double-blind phase) will be tabulated with exact 95% CI.

10.8.2.2. Secondary safety endpoints

Safety will be analyzed on the Total Vaccinated Cohort. If the percent of enrolled subjects excluded from the ATP cohort for analysis of safety is more than 5%, a second analysis based on this ATP cohort will be performed to complement the Total Vaccinated Cohort analysis.

The percentage of subjects with at least one local adverse event (solicited and unsolicited), with at least one general adverse event (solicited and unsolicited) and with any adverse event during the solicited follow-up period will be tabulated with exact 95% CI after each vaccine dose and overall. The percentage of doses followed by at least one local adverse event (solicited and unsolicited), by at least one general adverse event (solicited and unsolicited) and by any adverse event will be tabulated, overall vaccination course, with exact 95% CI. Similar tables will be generated for Grade 3 events, the relationship of the event to vaccination.

The percentage of subjects reporting each individual solicited local and general adverse event during the solicited follow-up period will be tabulated with exact 95% CI. The percentage of doses followed by each individual solicited local and general adverse event will be tabulated, overall vaccination course, with exact 95% CI. Similar tables will be generated for Grade 3 events, the relationship of the event to vaccination and for fever and temperature in 0.5°C increments.

The proportion of subjects reporting an AE (unsolicited) 30 days post each vaccination, classified by the MedDRA preferred term level, reported from study start until the end of the double-blind phase will be tabulated with exact 95% CI.

Biochemistry (ALT and creatinine) and hematology (hemoglobin, WBC, platelets) values that are outside of the reference ranges will be described for Clinic Visit 9 (end of double-blind phase). Frequency distribution of results by toxicity grades will be tabulated by group.

10.8.3. Analyses of immunogenicity

The primary analysis will be based on the ATP cohort for analysis of immunogenicity. If the percent of enrolled subjects excluded from this ATP cohort is more than 5%, a second analysis based on the Total Vaccinated cohort will be performed to complement the ATP analysis.

10.8.3.1. Anti-CS antibodies

The percentage of subjects seropositive for anti-CS (proportion of subjects with anti-CS antibody titers greater than or equal to 0.5 EU/mL) with 95% CI will be determined at each blood sampling time point. Antibody titers will be summarized by GMT with 95% CI at all time points at which serological samples are taken. Antibody titers 1 month after the last dose will also be investigated using reverse cumulative curves. Equivalence may be evaluated by looking at 95% CI on GMT ratios.

For all study groups, an area under the curve (AUC) will be estimated by trapezoidal rule using the 4 consecutive blood samples. The AUC will be corrected for the residual portion. Standardized AUCs (AUC divided by follow-up time) will also be calculated. Individual profiles of anti-CS responses will be plotted. Descriptive statistics (mean, median, quartiles, minimum and maximum) will be calculated by study group [Chow 1992; Matthews 1990]. Where applicable, study groups may be compared using Wilcoxon Rank Sum tests.

10.8.3.2. Anti-HBs antibodies

The seroprotective level for anti-HBs is ≥ 10 mIU/mL; The percentage of subjects with protective levels of anti-HBs (≥ 10 mIU/mL) with 95% confidence interval (95% CI) will be determined at will be determined at each blood sampling time point. Antibody titers will be summarized by GMT with 95% CI at will be determined at each blood sampling time point. Antibody titers after the third dose will also be investigated using reverse cumulative curves. Equivalence may be evaluated by looking at 95% CI on GMT ratios.

10.9. Annex analysis at study conclusion

10.9.1. Analyses of safety

The analysis of the occurrence of SAEs will be performed on the Total Vaccinated Cohort. The proportion of subjects with an SAE, classified by the MedDRA preferred term level, reported from study start until study conclusion (end of the single-blind phase) will be tabulated with exact 95% CI. Comparisons between groups will be done using Fisher's Exact test for each preferred term.

Serious adverse events and withdrawal due to adverse event(s) will be described in detail.

Biochemistry (ALT and creatinine) and hematology (hemoglobin, WBC, platelets) values that are outside of the reference ranges will be described for Clinic Visit 10 (end of single-blind phase).

10.9.2. Analyses of immunogenicity

The primary analysis will be based on the ATP cohort for analysis of immunogenicity. If the percent of enrolled subjects excluded from this ATP cohort is more than 5%, a second

analysis based on the Total Vaccinated cohort will be performed to complement the ATP analysis.

10.9.2.1. Anti-HBs antibodies

The seroprotective level for anti-HBs is ≥ 10 mIU/mL; The percentage of subjects with protective levels of anti-HBs (≥ 10 mIU/mL) with 95% confidence interval (95% CI) will be determined at Month 19 (Clinic Visit 10). Antibody titers will be summarized by GMT with 95% CI at Month 19 (Clinic Visit 10). Antibody titers after the third dose will also be investigated using reverse cumulative curves. Equivalence may be evaluated by looking at 95% CI on GMT ratios.

10.9.2.2. Anti-CS antibodies

The percentage of subjects seropositive for anti-CS (proportion of subjects with anti-CS antibody titers greater than or equal to 0.5 EU/mL) with 95% CI will be determined at Clinic Visit 10. Antibody titers will be summarized by GMT with 95%CI. Equivalence may be evaluated by looking at 95% CI on GMT ratios.

10.10. Exploratory Analyses

Details for the analysis of exploratory endpoints will be described in the Report Analysis Plan

10.11. Planned interim analysis

No interim analysis is planned in this study. Final analysis will be carried out at Month 10.

11. ADMINISTRATIVE MATTERS

To comply with Good Clinical Practice important administrative obligations relating to investigator responsibilities, monitoring, archiving data, audits, confidentiality and publications must be fulfilled. See Appendix B for details.

12. REFERENCES

Afrane YA, Klinkenberg E, Drechsel P, Owusu-Daaku K, Garms R and Kruppa T. Does irrigated urban agriculture influence the transmission of malaria in the city of Kumasi, Ghana? *Acta Tropica*. 2004: 125-134.

Ajjan N, Soulebot JP, Stellmann C, Biron G, Charbonnier C, Triau R et al. Results of preventive rabies vaccination with a concentrated vaccine of the PM/WI38-1503-3M rabies strain cultured on human diploid cells. Preparation of mixed antirabies-antitetanus hyperimmune immunoglobulin by plasmapheresis of blood taken from vaccinated veterinary students. *Dev Biol Stand* 1978; 40: 89-100.

Alloueche A, Milligan P, Conway DJ, Pinder M, Bojang K, Doherty T et al. Protective efficacy of the RTS,S/AS02 *Plasmodium falciparum* malaria vaccine is not strain specific. *American Journal of Tropical Medicine and Hygiene.* 2003; 68: 97-101.

Alonso PL, Sacarlal J, Aponte JJ, Leach A, Macete E et al. Efficacy of the RTS,S/AS02A vaccine against *Plasmodium falciparum* infection and disease in young African children: randomised controlled trial. *The Lancet*. 2004; 364: 1411-1420.

Alonso PL, Sacarlal J, Aponte JJ, Leach A, Macete E et al. Duration of protection with RTS,S/AS02A malaria vaccine in prevention of *Plasmodium falciparum* disease in Mozambican children: single-blind extended follow-up of a randomised controlled trial. *The Lancet.* 2005; 366: 2012-2018.

Bojang KA, Milligan PJM, Pinder M, Vigneron L, Alloueche A, Kester KE et al. Efficacy of RTS,S/AS02 Malaria Vaccine against *Plasmodium falciparum* Infection in Semi-Immune Adult Men in the Gambia: A Randomized Trial. *The Lancet*. 2001; 358: 1927-34.

Bonhoeffer J, Menkes J, Gold, MS, de Souza-Brito G, Fisher MC et al. Generalized convulsive seizure as an adverse event following immunization: case definition and guidelines for data collection, analysis and presentation. *Vaccine*. 2004; 22: 557-562.

Breman JG, Egan A, Keusch GT. Introduction and Summary. The Intolerable Burden of Malaria: A New Look at the Numbers. *American Journal of Tropical Medicine and Hygiene*. 2001 a; 64 (1, 2) S: iv-vii.

Breman, JG. The Ears of the Hippopotamus: Manifestations, Determinants and Estimates of the Malaria Burden. *American Journal of Tropical Medicine and Hygiene* 2001 b; 64: 1-11.

Chow SC, Liu JP. *Design and Analysis of Bioavailability and Bioequivalence (Statistics: Textbooks and Monographs)*. First Edition. New York: Marcel Dekker Ltd; 1992.

Costy-Berger F. Preventive rabies vaccination using vaccine prepared from human diploid cells. *Dev Biol Stand* 1978; 40: 101-104.

Cox JH, Schneider LG. Prophylactic immunization of humans against rabies by intradermal inoculation of human diploid cell culture vaccine. *J Clin Microbiol* 1976; 3 (2): 96-101.

Dreesen DW, Fishbein DB, Kemp DT, Brown J. Two-year comparative trial on the immunogenicity and adverse effects of purified chick embryo cell rabies vaccine for pre-exposure immunization. *Vaccine* 1989; 7 (5): 397-400.

Epstein JE, Charoenvit Y, Kester KE, Wang R, Newcomer R, Fitzpatrick S et al. Safety, tolerability, and antibody responses in humans after sequential immunization with a PfCSP DNA vaccine followed by the recombinant protein vaccine RTS,S/AS02A. *Vaccine.* 2004; 22: 1592–1603.

Gallup JL, Sachs JD. The Economic Burden of Malaria. *American Journal of Tropical Medicine and Hygiene*. 2001; 64: 85-96.

GSK data on file: Investigator Brochure GlaxoSmithKline Biologicals' Candidate Vaccines Against *Plasmodium falciparum* Malaria (RTS,S/AS02A, RTS,S/AS02D and RTS,S/AS01B Malaria Vaccines). 23 May 2005. Data on File at GlaxoSmithKline Biologicals.

GSK data on file: Safety, Immunogenicity and Efficacy of Three Adjuvant Preparations of a *Plasmodium falciparum* Vaccine (RTS,S) Which Utilizes the Hepatitis B Surface Antigen (HBsAg) as a Carrier for the Malaria Circumsporozoite (CS) Antigen. Report WRMAL-003. 24 August 1996. Data on File at GlaxoSmithKline Biologicals.

GSK data on file: Safety, Immunogenicity, and Preliminary Efficacy of a Lyophilized Formulation of SmithKline Beecham Biologicals' Candidate Malaria Vaccine RTS,S. Report WRMAL-005. 19 August 1999. Data on File at GlaxoSmithKline Biologicals.

GSK data on file: Dosage and Schedule Optimization of RTS,S Malaria Vaccine. Report WRMAL-004. 30 April 1998. Data on File at GlaxoSmithKline Biologicals.

GSK data on file: Phase IIb controlled randomized double-blind study to evaluate the efficacy, safety and immunogenicity of SmithKline Beecham Biologicals' candidate RTS,S malaria vaccine in semi-immune adult males in The Gambia. Protocol 257049/005 (Malaria-005) 12 December 1997. Data on File at GlaxoSmithKline Biologicals.

GSK data on file: Phase I/IIa safety, immunogenicity, and preliminary efficacy of two short-course schedules of SmithKline Beecham Biologicals' candidate malaria vaccine RTS,S in non-immune adult volunteers. Report 257049/012 (Malaria-012) 01 March 2002. Data on File at GlaxoSmithKline Biologicals.

GSK data on file: A Phase I Double Blind, Randomized, Controlled, Staggered Study to Evaluate the Safety, Reactogenicity and Immunogenicity of 1/5, 1/2 and Full Doses of GlaxoSmithKline Biologicals' RTS,S/AS02 Candidate Malaria Vaccine, Administered IM According to a 0, 1, 3-month Vaccination Schedule in Semi-Immune Children aged 6 to 11 Years in The Gambia, a Malaria Endemic Region. Report 257049/015 (Malaria-015) 14 October 2003. Data on File at GlaxoSmithKline Biologicals.

GSK data on file: Phase II b controlled randomized double-blind study to evaluate the efficacy, safety and immunogenicity of GlaxoSmithKline Biologicals' candidate RTS,S malaria vaccine in semi-immune adult males in The Gambia. Report 257049/016, 257049/017 & 257049/018 (Malaria-016, -017 & -018) 12 January 2005. Data on File at GlaxoSmithKline Biologicals.

GSK data on file: A Phase I Double Blind, Randomized, Controlled, Staggered Study to Evaluate the Safety, Reactogenicity and Immunogenicity of 1/5, 1/2 and Full Doses of GlaxoSmithKline Biologicals' RTS,S/AS02 Candidate Malaria Vaccine, Administered IM According to a 0, 1, 3-month Vaccination Schedule in Semi-Immune Children aged 1 to 5 Years in The Gambia, a Malaria Endemic Region. Report 257049/020 (Malaria-020) 27 February 2003. Data on File at GlaxoSmithKline Biologicals.

GSK data on file: A Phase I Double Blind, Randomized, Controlled, Staggered Study to Evaluate the Safety, Reactogenicity and Immunogenicity of GlaxoSmithKline Biologicals' RTS,S/AS02 Candidate Malaria Vaccine, Administered IM According to a 0, 1, 2-month Vaccination Schedule in Children aged 1 through 4 years in Mozambique, a Malaria Endemic Region. Report 257049/025 (Malaria-025) 10 October 2003. Data on File at GlaxoSmithKline Biologicals.

GSK data on file: A double-blind randomised controlled phase IIb study to evaluate the safety, immunogenicity and efficacy of GlaxoSmithKline Biologicals' candidate malaria vaccine RTS,S/AS02A, administered IM according to a 0, 1 and 2 month vaccination schedule in toddlers and children aged 1 to 4 years in a malaria-endemic region of Mozambique. Report 257049/026 (Malaria-026) 19 November 2004. Data on File at GlaxoSmithKline Biologicals.

GSK data on file: Double-blind, randomized phase I/IIa human challenge study, to evaluate the safety, reactogenicity, immunogenicity, and preliminary efficacy after primary sporozoite challenge and rechallenge, of GSK Biologicals' candidate malaria vaccines containing the antigen RTS,S adjuvanted with either AS02A or AS01B and administered intramuscularly at months 0, 1, 2 in healthy malaria-naive volunteers aged 18 - 45 years. Protocol 257049/027 (Malaria-027) 24 July 2003. Data on File at GlaxoSmithKline Biologicals.

GSK data on file: Double-blind, randomized phase I/IIa human challenge study, to evaluate the safety, reactogenicity, immunogenicity, and preliminary efficacy after primary sporozoite challenge and rechallenge, of GSK Biologicals' candidate malaria vaccines containing the antigen RTS,S adjuvanted with either AS02A or AS01B and administered intramuscularly at months 0, 1, 2 in healthy malaria-naive volunteers aged 18 - 45 years. Report 257049/027 (Malaria-027) 01 February 2006. Data on File at GlaxoSmithKline Biologicals.

GSK data on file: A Phase I/II randomized double-blind bridging study to evaluate the safety and immunogenicity of GlaxoSmithKline Biologicals' candidate vaccines RTS,S/AS02D (0.5 mL dose) and RTS,S/AS02A (0.25 mL dose) administered IM according to a 0, 1, 2 month vaccination schedule in children aged 3 to 5 years living in a malaria-endemic region of Mozambique. Report 257049/034 (Malaria-034) 06 December 2004. Data on File at GlaxoSmithKline Biologicals.

Kester KE, McKinney DA, Tornieporth N, Ockenhouse CF, Heppner DG, et al. Efficacy of recombinant circumsporozoite protein vaccine regimens against experimental *Plasmodium falciparum* malaria. *Journal of Infectious Diseases*. 2001; 15:183(4):640-7.

Kuwert EK, Marcus I, Werner J, Iwand A, Thraenhart O. Some experiences with human diploid cell strain-(HDCS) rabies vaccine in pre- and post-exposure vaccinated humans. *Dev Biol Stand* 1978; 40: 79-88.

Lalvani A, Moris P, Voss G, Kester KE, Brookes R, Koutsoukos M. Potent induction of focused Th-1 type cellular and humoral immune responses by RTS,S/SBAS2 recombinant *P. falciparum* malaria vaccine. *Journal of Infectious Diseases*. 1999; 180:1656-64.

Matthews JN, Altman DG, Campbell MJ, Royston P. Analysis of serial measurements in medical research. *British Medical Journal* 1990; 300 (6719): 230-5.

Nicholson KG, Farrow PR, Bijok U, Barth R. Pre-exposure studies with purified chick embryo cell culture rabies vaccine and human diploid cell vaccine: serological and clinical responses in man. *Vaccine* 1987; 5 (3): 208-210.

Smith PG, Morrow RH. *Methods for Field Trials of Interventions against Tropical Diseases: A Toolbox*. Oxford University Press 1991: 152-155; 168-175.

Snow RW, Schellenberg, JR, Peshu, D, Forster, CR, Newton, PA et al. Periodicity and space-time clustering of severe childhood malaria on the coast of Kenya. *Trans. R. Soc. Trop. Med. Hyg*. 1990: 87 (4): 386-390.

Snow RW, de Azevedo Bastos, I, Lowe, EW, Kabiru, CG, Nevill, CG et al. Severe childhood malaria in two areas of markedly different falciparum transmission in east Africa. *Acta Trop*. 1994: 57 (4): 289-300.

Stoute JA, Slaoui M, Heppner DG, Momin P, Kester KE, Desmons P et al. A Preliminary Evaluation of a Recombinant Circumsporozoite Protein Vaccine against *Plasmodium falciparum* Malaria. *N Eng J Med* 1997; 336: 86-91

Stoute JA, Kester KE, Krzych U, Wellde BT, Hall T, White K et al. Long Term Efficacy and Immune Responses Following Immunization with the RTS,S Malaria Vaccine. *Journal of Infectious Diseases*. 1998; 178: 1139-44.

Sun P, Schwenk R, White K, Stoute JA, Cohen J, Ballou WR et al. Protective immunity induced with malaria vaccine, RTS,S, is linked to *Plasmodium falciparum* circumsporozoite protein-specific CD4(+) and CD8(+) T cells producing IFN-gamma. *Journal of Immunology*. 2003; 171 (12): 6961-7.

UNAIDS/WHO. Epidemiological Fact Sheet on HIV/AIDS and Sexually Transmitted Infections. Ghana. 2004 Update. New York: UNAIDS/WHO 2004.

Vodopija I, Sureau P, Lafon M, Baklaic Z, Ljubicic M, Svjetlicic M et al. An evaluation of second generation tissue culture rabies vaccines for use in man: a four-vaccine comparative immunogenicity study using a pre-exposure vaccination schedule and an abbreviated 2-1-1 postexposure schedule. *Vaccine* 1986; 4 (4): 245-248.

Vryheid RE, Kane MA, Muller N, Schatz GC, Bezabeh S. Infant and Adolescent Hepatitis B Immunization up to 1999: A Global Overview. *Vaccine* 2001;19: 1026-1037.

Wasi C, Chaiprasithikul P, Chavanich L, Puthavathana P, Thongcharoen P, Trishanananda M. Purified chick embryo cell rabies vaccine. *The Lancet* 1986; 1 (8471): 40.

World Health Organization. Hepatitis B Fact Sheet WHO/204. http://www.who.int/mediacentre/factsheets/fs204/en/index.html Accessed 02 February 2006.

World Health Organization. Draft Global Immunization Strategy, 28 April 2005. http://www.who.int/vaccines/GIVS/english/full_en.pdf. Accessed 02 February 2006.

Appendix A World Medical Association Declaration of Helsinki

Recommendations guiding physicians in biomedical research involving human subjects

Adopted by the 18th World Medical Assembly
Helsinki, Finland, June 1964

and amended by the
29th World Medical Assembly
Tokyo, Japan, October 1975
35th World Medical Assembly
Venice, Italy, October 1983
41st World Medical Assembly
Hong Kong, September 1989
and the
48th General Assembly
Somerset West, Republic of South Africa, October 1996

INTRODUCTION

It is the mission of the physician to safeguard the health of the people. His or her knowledge and conscience are dedicated to the fulfillment of this mission.

The Declaration of Geneva of the World Medical Association binds the physician with the words, "The health of my patient will be my first consideration," and the International Code of Medical Ethics declares that, "A physician shall act only in the patient's interest when providing medical care which might have the effect of weakening the physical and mental condition of the patient."

The purpose of biomedical research involving human subjects must be to improve diagnostic, therapeutic and prophylactic procedures and the understanding of the etiology and pathogenesis of disease.

In current medical practice most diagnostic, therapeutic or prophylactic procedures involve hazards. This applies especially to biomedical research.

Medical progress is based on research which ultimately must rest in part on experimentation involving human subjects.

In the field of biomedical research a fundamental distinction must be recognized between medical research in which the aim is essentially diagnostic or therapeutic for a patient, and medical research, the essential object of which is purely scientific and without implying direct diagnostic or therapeutic value to the person subjected to the research.

Special caution must be exercised in the conduct of research which may affect the environment, and the welfare of animals used for research must be respected.

Because it is essential that the results of laboratory experiments be applied to human beings to further scientific knowledge and to help suffering humanity, the World Medical Association has prepared the following recommendations as a guide to every physician in biomedical research involving human subjects. They should be kept under review in the future. It must be stressed that the standards as drafted are only a guide to physicians all over the world. Physicians are not relieved from criminal, civil and ethical responsibilities under the laws of their own countries.

I. BASIC PRINCIPLES

3. Biomedical research involving human subjects must conform to generally accepted scientific principles and should be based on adequately performed laboratory and animal experimentation and on a thorough knowledge of the scientific literature.
4. The design and performance of each experimental procedure involving human subjects should be clearly formulated in an experimental protocol which should be transmitted for consideration, comment and guidance to a specially appointed committee independent of the investigator and the sponsor provided that this independent committee is in conformity with the laws and regulations of the country in which the research experiment is performed.
5. Biomedical research involving human subjects should be conducted only by scientifically qualified persons and under the supervision of a clinically competent medical person. The responsibility for the human subject must always rest with a medically qualified person and never rest on the subject of research, even though the subject has given his or her consent.
6. Biomedical research involving human subjects cannot legitimately be carried out unless the importance of the objective is in proportion to the inherent risk to the subject.
7. Every biomedical research project involving human subjects should be preceded by careful assessment of predictable risks in comparison with foreseeable benefits to the subject or to others. Concern for the interests of the subject must always prevail over the interests of science and society.
8. The right of the research subject to safeguard his or her integrity must always be respected. Every precaution should be taken to respect the privacy of the subject and to minimize the impact of the study on the subject's physical and mental integrity and on the personality of the subject.
9. Physicians should abstain from engaging in research projects involving human subjects unless they are satisfied that the hazards involved are believed to be predictable. Physicians should cease any investigation if the hazards are found to outweigh the potential benefits.
10. In publication of the results of his or her research, the physician is obliged to preserve the accuracy of the results. Reports of experimentation not in accordance with the principles laid down in this Declaration should not be accepted for publication.
11. In any research on human beings, each potential subject must be adequately informed of the aims, methods, anticipated benefits and potential hazards of the

study and the discomfort it may entail. He or she should be informed that he or she is at liberty to abstain from participation in the study and that he or she is free to withdraw his or her consent to participation at any time. The physician should then obtain the subject's freely-given informed consent, preferably in writing.

12. When obtaining informed consent for the research project the physician should be particularly cautious if the subject is in a dependent relationship to him or her or may consent under duress. In that case the informed consent should be obtained by a physician who is not engaged in the investigation and who is completely independent of this official relationship.

13. In case of legal incompetence, informed consent should be obtained from the legal guardian in accordance with national legislation. Where physical or mental incapacity makes it impossible to obtain informed consent, or when the subject is a minor, permission from the responsible relative replaces that of the subject in accordance with national legislation.
Whenever the minor child is in fact able to give a consent, the minor's consent must be obtained in addition to the consent of the minor's legal guardian.

14. The research protocol should always contain a statement of the ethical considerations involved and should indicate that the principles enunciated in the present Declaration are complied with.

II. MEDICAL RESEARCH COMBINED WITH PROFESSIONAL CARE (Clinical research)

1. In the treatment of the sick person, the physician must be free to use a new diagnostic and therapeutic measure, if in his or her judgment it offers hope of saving life, re-establishing health or alleviating suffering.
2. The potential benefits, hazards and discomfort of a new method should be weighed against the advantages of the best current diagnostic and therapeutic methods.
3. In any medical study, every patient - including those of a control group, if any - should be assured of the best proven diagnostic and therapeutic method. This does not exclude the use of inert placebo in studies where no proven diagnostic or therapeutic method exists.
4. The refusal of the patient to participate in a study must never interfere with the physician–patient relationship.
5. If the physician considers it essential not to obtain informed consent, the specific reasons for this proposal should be stated in the experimental protocol for transmission to the independent committee (I, 2).
6. The Physician can combine medical research with professional care, the objective being the acquisition of new medical knowledge, only to the extent that medical research is justified by its potential diagnostic or therapeutic value for the patient.

III. NON-THERAPEUTIC BIOMEDICAL RESEARCH INVOLVING HUMAN SUBJECTS (Non-clinical biomedical research)

1. In the purely scientific application of medical research carried out on a human being, it is the duty of the physician to remain the protector of the life and health of that person on whom biomedical research is being carried out.
2. The subjects should be volunteers - either healthy persons or patients for whom the experimental design is not related to the patient's illness.
3. The investigator or the investigating team should discontinue the research if in his/her or their judgment it may, if continued, be harmful to the individual.

In research on man, the interest of science and society should never take precedence over considerations related to the well being of the subject.

Appendix B Administrative Matters

I. Responsibilities of the Investigator

- To ensure that he/she has sufficient time to conduct and complete the study and has adequate staff and appropriate facilities and equipment which are available for the duration of the study and to ensure that other studies do not divert essential subjects or facilities away from the study at hand.
- To submit an up-to-date curriculum vitae or Investigator Biography and other credentials (e.g., medical license number in the United States) to GSK and—where required—to relevant authorities. It is recommended that this documentation indicates any previous clinical research experience and history of training in GCP.
- To acquire the reference ranges for laboratory tests performed locally and, if required by local regulations, obtain the laboratory's current certification or Quality Assurance procedure manual.
- To ensure that no clinical samples (including serum samples) are retained on site or elsewhere without the approval of GSK Biologicals and the express written informed consent of the subject and/or the subject's legally authorized representative.
- To perform no other biological assays at the investigator site except those described in the protocol or its amendment(s).
- To prepare and maintain adequate subject source data or raw data designed to record observations, and other data pertinent to the study.
- To conduct the study in compliance with the protocol any amendment and "Good Clinical Practice" (GCP) and all applicable regulatory requirements.
- To co-operate with a representative of GSK Biologicals in the monitoring process of the study and in resolution of queries about the data.
- To permit drug regulatory agencies and GSK audits.

II. Protocol Amendments and Administrative changes

- No changes to the study protocol will be allowed unless discussed in detail with the GSK Biologicals' Clinical Development Manager/Medical Monitor and filed as an amendment/administrative change to this protocol.
- Any amendment/administrative change to the protocol will be adhered to by the participating centre(s) and will apply to all subjects. Written IRB/IEC approval of protocol amendments is required prior to implementation, except where permitted by all applicable regulatory requirements; administrative changes and amendments not submitted for approval are submitted to IRBs/IECs for information only.
- Submission of protocol amendments to regulatory agencies will occur in accordance with local regulatory requirements. For some countries, submission to the local regulatory authority may not be required. When submission to the local regulatory authority is required, the timing of the submission relative to IEC/IRB submission or approval and whether or not the authority will provide their approval of or

favourable opinion on the amendment before it can be implemented will depend on local regulatory requirements.

III. Sponsor's Termination of Study

GSK Biologicals reserves the right to temporarily suspend or prematurely discontinue this study either at a single site or at all sites at any time for reasons including, but not limited to, safety or ethical issues or severe non-compliance. Reasons for suspension or early termination will be documented in the study file at GSK Biologicals.

If GSK Biologicals determines that suspension or early termination is needed, GSK Biologicals will discuss this with the Investigator (including the reasons for taking such action). When feasible, GSK Biologicals will provide advance notification to the investigator of the impending action prior to it taking effect.

GSK Biologicals will promptly inform, via written communication, all investigators and/or institutions conducting the study, if the study is suspended or terminated for safety reasons, and will also inform the regulatory authorities of the suspension or termination of the study and the reason(s) for the action. If required by applicable regulations, the investigator must inform the IEC/IRB promptly and provide the reason for the suspension or termination.

If the study is prematurely discontinued, all study data must be returned to GSK. In addition, arrangements will be made for all unused investigational product(s) in accordance with the applicable GSK procedures for the study. Financial compensation to investigators and/or institutions will be in accordance with the agreement established between the investigator and/or institutions and GSK.

IV. Case Report Form Instructions

Prior to screening the first potential participant, the investigator will provide the Site Monitor with a list (Site Staff Signature Sheet) showing the name and title, signature and initials of all site staff who have a critical effect on the conduct of the study and to whom the investigator has delegated significant study related duties such as entering data on the CRFs or changing entries on CRFs. If the authorized individuals should change during the study, the investigator is to inform GSK Biologicals GSK Biologicals' representative of the specific change(s).

CRFs (and subject diary cards, if applicable), will be supplied by GSK Biologicals for recording all data. It is the responsibility of the investigator or co-investigator to ensure that study data are legible, accurate, adequately recorded and, when entered on paper copy, completely filled in with a black ink fountain or ballpoint pen.

Errors must be corrected by drawing a single line through the incorrect entry and writing in the new value/data positioned as close to the original as possible. The correction must then be initialed and dated (and justified, whenever possible), where necessary, by the authorized individual making the change. The original entry must not be obliterated, overwritten or erased when a correction is made.

When a subject completes a visit, it is anticipated that relevant sections of the CRF will be completed by the investigator (or designated staff as documented in the Site Staff Signature Sheet) as soon as possible after the last data becoming available. Similarly, when a subject completes a study, it is anticipated that all relevant CRF pages will be completed promptly after the last data becoming available. This also applies to forms for potential study participants who were screened but not randomized to a study group.

As soon as the subject has completed/withdrawn from the study and the CRF is completed, the investigator or medically qualified sub-investigator to whom this task has been delegated will sign the study conclusion pages of the CRF to confirm that they have reviewed the data and that the data are complete and accurate. In all cases the investigator remains accountable for the study data collected.

An original (top copy) CRF or log sheets must be submitted for all subjects who have undergone protocol specific procedures, whether or not the subject completed the study.

While completed CRFs are reviewed by a GSK Biologicals' professional monitor at the study site, errors detected by subsequent in-house CRF review may necessitate clarification or correction of errors with documentation and approval by the investigator or appropriately qualified designee as documented on the Site Staff Signature Sheet. In all cases, the investigator remains accountable for the study data. Wherever possible the investigator should assist in the clarification or correction of errors detected after study finalization promptly after being brought to the attention of the investigator (preferably within 5 working days).

Any questions or comments related to the CRF should be directed to the assigned Site Monitor.

V. Monitoring by GSK Biologicals

To ensure compliance with the protocol, monitoring visits by a professional representative of the sponsor will be scheduled to take place early in the study, during the study at appropriate intervals and after the last subject has completed the study. It is anticipated that monitoring visits will occur at a frequency defined and communicated to the investigator before study start.

These visits are for the purpose of confirming that GSK Biologicals' sponsored studies are being conducted in accordance with the ethical principles that have their origins in the Declaration of Helsinki and that are consistent with Good Clinical practice (GCP) and the applicable regulatory requirement(s) (verifying continuing compliance with the protocol, amendment(s), reviewing the investigational product accountability records, verifying that the site staff and facilities continue to be adequate to conduct the study. Direct access to all study-related site and source data/ documents is mandatory for the purpose of monitoring review. The monitor will perform a CRF review and a Source Document verification (verifying CRF/ RDE entries by comparing them with the source data/documents that will be made available by the investigator for this purpose: any data item for which the CRF will serve as the source must be identified, agreed and documented. Data to be recorded directly into the CRF pages/RDE screens will be specified in writing preferably in the source documentation agreement form that is

contained in both the monitor's and investigator's study file. For RDE, the monitor will mark completed and approved screens at each visit. The investigator must ensure provision of reasonable time, space and adequate qualified personnel for monitoring visits. Source data verification (SDV) must be conducted using a GSK standard SDV sampling strategy (as defined at the study start in the study specific monitoring guidelines) in which monitors will perform partial SDV for all subjects and full SDV for selected subjects.

VI. Archiving of Data

Following closure of the study, the investigator must maintain all site study records in a safe and secure location. The records must be maintained to allow easy and timely retrieval, when needed (e.g., audit or inspection), and, whenever feasible, to allow any subsequent review of data in conjunction with assessment of the facility, supporting systems, and staff. Where permitted by applicable laws/regulations or institutional policy, some or all of these records can be maintained in a validated format other than hard copy (e.g., microfiche, scanned, electronic for studies with an eCRF, for example); however, caution needs to be exercised before such action is taken. The investigator must assure that all reproductions are legible and are a true and accurate copy of the original, and meet accessibility and retrieval standards, including re-generating a hard copy, if required. Furthermore, the investigator must ensure there is an acceptable back-up of these reproductions and that an acceptable quality control process exists for making these reproductions.

GSK will inform the investigator/ institution of the time period for retaining these records to comply with all applicable regulatory requirements. However, the investigator/ institution should seek the written approval of the sponsor before proceeding with the disposal of these records. The minimum retention time will meet the strictest standard applicable to that site for the study, as dictated by ICH GCP E6 Section 4.9, any institutional requirements or applicable laws or regulations, or GSK standards/procedures; otherwise, the minimum retention period will default to 15 years.

The investigator/ institution must notify GSK of any changes in the archival arrangements, including, but not limited to, the following: archival at an off-site facility, transfer of ownership of the records in the event the investigator leaves the site.

VII. Audits

For the purpose of compliance with Good Clinical Practice and Regulatory Agency Guidelines it may be necessary for GSK or a Drug Regulatory Agency to conduct a site audit. This may occur at any time from start to after conclusion of the study.

When an investigator signs the protocol, he agrees to permit drug regulatory agencies and GSK audits, providing direct access to source data/ documents. Furthermore, if an investigator refuses an inspection, his data will not be accepted in support of a New Drug Registration and/or Application, Biologics Licensing Application.

Having the highest quality data and studies are essential aspects of vaccine development. GSK has a Regulatory Compliance staff who audit investigational sites. Regulatory

Compliance assesses the quality of data with regard to accuracy, adequacy and consistency. In addition, Regulatory Compliance assures that GSK Biologicals' sponsored studies are in accordance with GCP and that relevant regulations/guidelines are being followed.

To accomplish these functions, Regulatory Compliance selects investigational sites to audit. These audits usually take 1 to 2 days. GSK's audits entail review of source documents supporting the adequacy and accuracy of CRFs, review of documentation required to be maintained, and checks on vaccine accountability. GSK's audit therefore helps prepare an investigator for a possible regulatory agency inspection as well as assuring GSK Biologicals of the validity of the database across investigational sites.

The Inspector will be especially interested in the following items:

- Log of visits from the sponsor's representatives
- Study personnel
- Study file
- Safety reporting
- IRB/IEC and regulatory authority approvals
- Facilities
- monitoring
- Vaccine accountability
- Approved study protocol and amendments and investigator brochure
- Informed consent of the subjects (written consent [or witnessed oral if applicable])
- Medical records and other source documents supportive of CRF data
- Reports to the IRB/IEC and the sponsor
- Record retention.

GSK Biologicals will gladly help investigators prepare for an inspection.

VIII. Ownership, Confidentiality and Publication

Ownership:

All information provided by GSK and all data and information generated by the site as part of the study (other than a subject's medical records) are the sole property of GSK.

All rights, title, and interests in any inventions, know-how or other intellectual or industrial property rights which are conceived or reduced to practice by site staff during the course of or as a result of the study are the sole property of GSK, and are hereby assigned to GSK.

If a written contract for the conduct of the study which includes ownership provisions inconsistent with this statement is executed between GSK and the study site, that contract's ownership provisions shall apply rather than this statement.

Confidentiality:

Documented evidence that a potential investigator is aware and agrees to the confidential nature of the information related to the study must be obtained by means of a confidentiality agreement.

All information provided by GSK and all data and information generated by the site as part of the study (other than a subject's medical records) will be kept confidential by the investigator and other site staff. This information and data will not be used by the investigator or other site personnel for any purpose other than conducting the study. These restrictions do not apply to: (1) information which becomes publicly available through no fault of the investigator or site staff; (2) information which it is necessary to disclose in confidence to an IEC or IRB solely for the evaluation of the study; (3) information which it is necessary to disclose in order to provide appropriate medical care to a study subject; or (4) study results which may be published as described in the next paragraph. If a written contract for the conduct of the study which includes confidentiality provisions inconsistent with this statement is executed, that contract's confidentiality provisions shall apply rather than this statement.

Publication:

For multicenter studies, the first publication or disclosure of study results shall be a complete, joint multicenter publication or disclosure coordinated by GSK. Thereafter, any secondary publications will reference the original publication(s).

Prior to submitting for publication, presentation, use for instructional purposes, or otherwise disclosing the study results generated by the site (collectively, a "Publication"), the investigator shall provide GSK with a copy of the proposed Publication and allow GSK a period to review the proposed Publication (at least 21 (twenty-one) days [or at least 15 (fifteen) working days for abstracts/posters/presentations]. Proposed Publications shall not include either GSK confidential information other than the study results or personal data on any subject, such as name or initials.

At GSK's request, the submission or other disclosure of a proposed Publication will be delayed a sufficient time to allow GSK to seek patent or similar protection of any inventions, know-how or other intellectual or industrial property rights disclosed in the proposed Publication.

If a written contract for the conduct of the study, which includes publication provisions inconsistent with this statement is executed, that contract's publication provisions shall apply rather than this statement.

Appendix C Overview of the Recruitment Plan

KCCR/SMS

Prior to enrollment, a specific information policy towards the targeted population will be implemented. This will consist of a step-by-step approach, starting with the administrative and senior leaders of the community as well as health personnel from the different health facilities in the study area. Agogo Town, from where the study participants will come, is divided into approximately ten assembly areas and following the meeting with senior leaders it will be decided how best to hold information sessions in each small area. In the past meetings have been held in churches and community centres. The sessions will explain the problem of malaria to this community, the current strategies for its control, as well as the limitations of these strategies. The need and the difficulties of developing a vaccine against malaria will be discussed, as well as an outline of the proposed trials, including the rationale, the background data available and the study objectives. Particular attention will be paid to study procedures, immunization and blood collection. In that respect a full discussion on the purpose of blood collection and the associated risks will be carried out.

A register is currently being made of children from the town; this will include a listing of which children, according to age, will be eligible for the study. The register is being created from children attending the current immunization clinics in the town. A senior field worker is noting the children's name, age and address and following the local information sessions the parents of these children will be invited to attend specific small-group information sessions. Following this, a list of children whose mothers have expressed an interest in enrolling them in the study will be compiled and the SOPs on home follow-up before enrolment will be implemented. Study personnel will seek individual informed consent for each child from the parent(s)/guardian(s) in privacy. Parent(s)/guardian(s) will again be informed about the study objectives and procedures including immunization and blood collection and they will be encouraged to ask and clarify questions about the trial. The parent(s)/guardian(s) understanding of the Informed Consent form will be verified by use of an oral assessment questionnaire (outlined in SOPs). The parent(s)/guardian(s) and the witness will be invited to sign the Informed Consent Form. Children whose parent(s)/guardian(s) consent for them to enter the study will be invited to come to the maternal child health centre and be screened according to the procedures outlined in the Study Protocol.

KHRC

The Kintampo site has a demographic surveillance system (DSS) in place that records the location of all compounds/houses in the area. The residents of each compound are registered at household levels with permanent identification numbers. This registration system enables staff to trace people to their homes whenever required. The records of each resident are updated every six months by specialized centre staff members. Staff visit households to record births, deaths and migrations to and from the area. The DSS will be used to compile a list of potentially eligible children.

Discussion sessions will be held with the local community prior to recruitment. These preliminary sessions will be held with opinion leaders, health personnel from the

different health facilities, chiefs, elders, religious leaders and assembly-men. During these sessions the malaria menace will be discussed as well as the current control strategies, the limitations of these strategies and new innovative strategies being developed.

The need for a vaccine against malaria will be discussed, followed by a careful explanation of what this new malaria vaccine trial plans to achieve. Particular attention will be paid to study procedures, including immunization and blood collection. This will be followed by an open discussion regarding any concerns that people may have.

If the participants at the discussion sessions endorse community participation in the project, field staff from the centre will visit parents/guardians of children on the list compiled from the DSS at their homes. Field staff will have a thorough one-on-one briefing. Following the briefing, staff will register whether or not the parents are willing to allow their child to participate in the study. Results from the one-on-one briefings will be sent to the centre for a new database to be created for eligible children and willing parents. An SOP on home follow-up for invitation for screening/ enrolment will be developed, and staff will be trained in readiness for the recruitment. This SOP will also involve individual informed consent seeking for each child from the parent(s)/guardian(s) in privacy using the detailed consent form of the study.

Children whose parent(s)/guardian(s) consent for them to enter the study will be informed of their screening date; a worker will be sent early on the day of screening to prepare the parent(s)/guardian(s) and their child while a vehicle follows to pick those invited to the screening area in the Kintampo District Hospital. Parent(s)/guardian(s) who consent for their child to take part in the study will be given a summary of the ICF to remind them of the study procedures and requirements.

It will be ensured that staff are adequately trained on their roles in the study, a practice session will be held before recruitment/screening/enrolment commences.

Appendix D Handling of Biological Samples Collected by the Investigator

Instructions for Handling of Serum Samples

When materials are provided by GSK Biologicals, it is MANDATORY that all clinical samples (including serum samples) will be collected and stored using exclusively those materials in the appropriate manner. The use of other materials could result in the exclusion of the subject from the ATP analysis. The investigator must ensure that his/her personnel and the laboratory(ies) under his/her supervision comply with this requirement. However, when GSK Biologicals does not provide material for collecting and storing clinical samples, then appropriate materials from the investigator's site are to be used.

1. Collection

The whole blood should be collected observing appropriate aseptic conditions. It is recommended that Vacutainer® tubes WITH integrated serum separator (e.g. Becton-Dickinson Vacutainer SST or Corvac® Sherwood Medical) be used to minimize the risk of hemolysis and to avoid blood cell contamination of the serum when transferring to standard serum tubes.

2. Serum separation

These guidelines aim to ensure high quality serum by minimizing the risk of hemolysis, blood cell contamination of the serum or serum adverse cell toxicity at testing.

- For separation of serum using Vacutainer tubes, the instructions provided by the manufacturer should be followed. Siliconized tubes should never be used (cell toxicity). Often the manufacturer's instruction states that the relative centrifugal acceleration known also as "G" must be "between 1000 and 1300 G" with tubes spinning for ten minutes. Error in calculation of centrifuge speed can occur when laboratory personnel confuse "G" acceleration with "RPM" (revolutions per minute). The speed of centrifugation must be calculated using the "G" rate provided in the manufacturer's instructions and the radius of the centrifuge head. After measuring the radius of the centrifuge machine, a speed/acceleration nomograph must be employed to determine the centrifuge speed in "RPM".
- Following separation, the serum should be aseptically transferred to the appropriate standard tubes using a sterile disposable pipette. The serum should be transferred as gently as possible to avoid blood cell contamination.
- The tube should not be overfilled (max. 3/4 of the total volume) to allow room for expansion upon freezing.
- The tube should be identified by the appropriate label provided by GSK Biologicals (see point 3).

3. Labeling

- The standard labels provided by GSK Biologicals should be used to label each serum sample.

- If necessary, any hand-written additions to the labels should be made using indelible ink.
- The label should be attached to the tube as follows (see diagram):
  - first attach the paper part of the label to the tube
  - then wrap the label around the tube so that the transparent, plastic part of the label overlaps with the label text and bar code and shields them.

  This will ensure optimal label attachment.

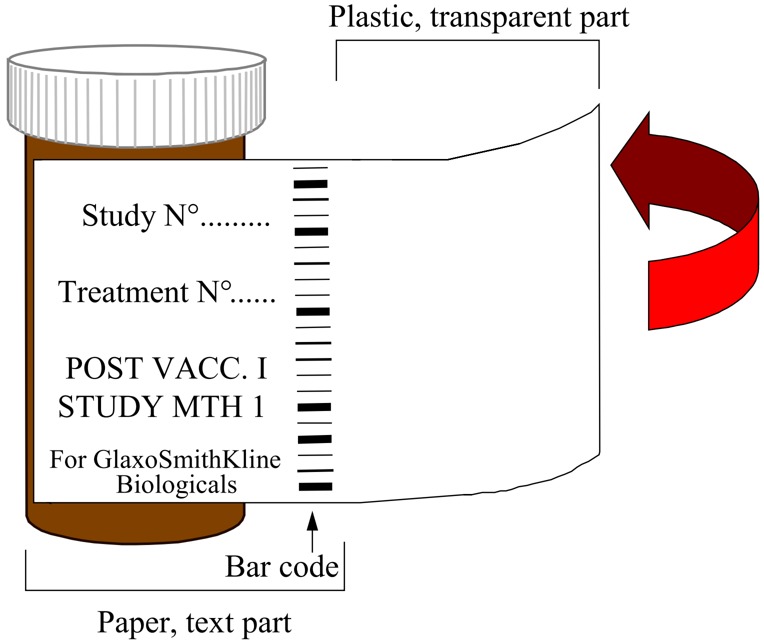

Labels should not be attached to caps.

4. Sorting and storage

- Tubes should be placed in the GSK Biologicals' cardboard boxes in numerical order from left to right, starting from the lower left hand corner, beginning with the pre-vaccination samples series, then with the post-vaccination sample series.
- The tubes of serum should be stored in a vertical position at approximately -20°C (alternatively at approximately -70°/80°C is also acceptable) until shipment to GSK Biologicals. The storage temperature should be checked regularly and documented. Wherever possible, a backup facility for storage of serum samples should be available.
- A standard Serum Listing Form, specifying the samples being shipped for individual subjects at each time point, should be prepared for each shipment. A copy of this list should be retained at the study site, while the original should be sealed in a plastic envelope and shipped with the serum samples.

- Once flight details are known, a standard Specimen Transfer Form must be completed and faxed to GSK Biologicals to the number provided below. A copy of the Specimen Transfer Form must be in the parcel1

GLAXOSMITHKLINE BIOLIGICALS
Attention Biospecimen Reception
Clinical Immunology
R & D Department/Building 44
Rue de l'Institut, 89
B-1330 Rixensart – Belgium

Telephone	+32-2-656 8949 or +32-2-656 6130
	or +32-2-656 8549 or +32-2-656 6108
Fax	+32-2-656 6052
E-mail	rix.ugbiospecimen-reception@gskbio.com

Instructions for Handling Cells for Cell-Mediated Immunity Assay

When materials are provided by GSK Biologicals, it is mandatory that all clinical samples be collected and stored using exclusively those materials in the appropriate manner. The use of other materials could result in the exclusion of the subject from analysis. The investigator must ensure that his/her personnel and the laboratory(ies) under his/her supervision comply with this requirement.

1. Collection of whole blood

Collect blood by venipuncture in Terumo tubes with heparin (mandatory) and record time of collection. The tubes should be kept at constant room temperature and shipped as quickly as possible to a designated clinical site. Use well closed Styrofoam boxes of 5 cm thickness for blood samples transport (see current version of GSK Biologicals SOP RD_HCI_001 for guidance).

2. Labeling of tubes

- If labels are provided by GSK, it is mandatory to use them.
- If necessary, any hand-written additions to the labels should be made using indelible ink.

4. Sorting and storage of blood cell samples

Samples should be stored frozen (<-80°C) until shipment to GSK Biologicals. Wherever possible, a backup facility for storage of samples should be available.

[1] The Serum Listing Form and the Specimen Transfer Form are standard documents used in GSK Biologicals' clinical trials. These documents are provided by GSK Biologicals' Clinical Trials' monitor at study initiation.

A standard Cryotube Listing Form (see current version of GSK Biologicals SOP RD_HCI_009 for guidance), specifying the samples being shipped for individual subjects at each timepoint, should be prepared for each shipment. A copy of this list should be retained at the study site, while the original should be sealed in a plastic envelope and shipped with the samples.

Appendix E Shipment of Biological Samples

Instructions for shipment of serum and cell samples

Serum and cell samples should be sent to GSK Biologicals at regular intervals. The frequency of shipment of samples should be decided upon by the Site Monitor, Central Study Coordinator and the investigator prior to the study start.

Serum and cell samples should always be sent by air, preferably on a Monday, Tuesday or Wednesday, unless otherwise requested by the sponsor.

Serum and cell samples must be placed with dry ice (maximum -20°C) in a container complying with International Air Transport Association (IATA) requirements. The completed standard serum listing form should always accompany the shipment.

The container must be clearly identified with the labels provided by GSK Biologicals specifying the shipment address and the storage temperature (-20°C).

The airway bill should contain the instruction for storage of samples at maximum -20°C.

A 'proforma' invoice, stating a value for customs purposes only, should be prepared and attached to the container. This document should contain the instruction for storage of samples at maximum -20°C.

Details of the shipment, including:

* number of samples
* airway bill
* flight number
* flight departure and arrival times

should be sent by fax or email two days before shipment, to:

GLAXOSMITHKLINE BIOLOGICALS,
Attention Biospecimen Reception
Clinical Immunology
R & D Department/Building 44
Rue de l'Institut, 89
B-1330 Rixensart – Belgium

Telephone +32-2-656 8949 or +32-2-656 6130
or +32-2-656 8549 or +32-2-656 6108
Fax +32-2-656 6052
E-mail rix.ugbiospecimen-receiption@gskbio.com

The central study coordinators, Nathalie Annez and Arlette Simonon should be informed 2 days before any shipment.

Nathalie Annez, Rixensart, Belgium
Telephone: +32.2.656.57.48
Fax: +32.2.656.80.44
email: nathalie.annez@gskbio.com

Arlette Simonon
Telephone: +32.2.656.55.07
Fax: +32.2.656.80.44
email: arlette.simonon@gskbio.com

Appendix F Laboratory Assays

Serology testing

Serological responses will be measured principally by evaluating antibody responses to HBs and to CSP repeats (anti R32LR). Serum for antibody determination will be collected at the time points defined in the flowcharts in protocol Section 5.11.

Antibody levels against CS will be measured at GSK Biologicals (or a designated laboratory) by standard ELISA methodology using plate adsorbed R32LR antigen with a standard reference antibody as a control according to SOPs from the laboratory. Results will be reported in EU/mL.

Antibody to hepatitis B surface antigen will be measured at GSK Biologicals using a commercially available ELISA immunoassay (AUSAB EIA test kit from Abbott) or equivalent according to the assay instructions. Results will be reported in mIU/mL.

Determination of parasitemia

Estimates of asexual *P. falciparum* parasite density will be made at KHRC and KCCR/SMS according to laboratory SOPs. Two slides will be air dried, stained with Giemsa and read on a light microscope with a x50 oil immersion lens and x10 eyepieces. Parasite density will be assessed by counting the number of asexual stage parasites per 200 leukocytes. Slides will be declared negative only after 200 leukocytes have been read. Parasite numbers will be converted to a count/µL by assuming a standard leukocyte count of 8000/µL. All slides will be read twice independently, and a third time if the ratio of densities from the first two is greater than 1.5 or smaller than 0.67 or if there is a discrepancy in positivity. If less than 30 parasites are counted a third reading by a different reader will be done if the difference in the number of parasites is greater than 10. The definitive result will be based on the majority verdict for positivity and the geometric mean of the positive densities for positive slides.

Cell-mediated immunity

CMI will be investigated at two time points in this study, corresponding to one month post last dose of vaccination and at study conclusion.

Intracellular fluorescent staining for cytokines (ICS) is a method for detecting defined populations of cells (for example CD4+ or CD8+ T-cells expressing cytokines and activation markers upon in vitro stimulation).

Blood cells will be stimulated with CS-derived peptides for 20 hours and brefeldin added to prevent cytokine secretion. The cells will then be preserved for subsequent analysis in a classical ICS assay. This analyses will allow the determination of the frequencies of Ag-specific cells and determine the functionalities of these cells.

Biochemical and hematological analyses

Hematological and biochemical testing will be done at KHRC and KCCR/SMS in Ghana, following laboratory SOPs.

Appendix G Vaccine supplies, packaging and accountability

It is NOT permitted to use any of the supplies provided by GSK Biologicals for purposes other than those specified in the protocol. Unused supplies will be collected by GSK Biologicals on completion of the study. Used vaccine vials/pre-filled syringes/containers can be disposed on site according to local biosafety standard for disposal of biological waste material.

1. Vaccine supplies

GSK Biologicals will supply the following amounts of numbered doses of study vaccines, sufficient to administer sufficient doses to all subjects as described in the present protocol.

- Sufficient doses for ~~90~~ ***135*** recipients of the candidate vaccine RTS,S/AS02D on a three dose schedule and 90 recipients on a two dose schedule (doses of RTS,S vaccine in monodose vials and doses of AS02D adjuvant in pre-filled syringes). **Amended (04 July 2006)**
- Sufficient doses for 180 recipients of the candidate vaccine RTS,S/AS01E on a three dose schedule and 90 recipients on a two dose schedule (doses of RTS,S vaccine in monodose vials and doses of AS01E adjuvant in pre-filled syringes).
- Sufficient doses for ~~90~~ ***45*** recipients of Rabies vaccine on a three dose schedule. **Amended (04 July 2006)**

An additional 3% of their respective amounts of RTS,S/AS02D, RTS,S/AS01E and Rabies vaccine will be supplied for replacement in case of breakage, bad storage conditions or any other reason that would make the vaccine unusable (i.e. given by mistake to another subject).

All pre-filled syringes and vials must be accounted for on the form provided.

2. Vaccine packaging

The vaccines will be packed in labeled boxes. The box label will contain, as a minimum, the following information: study number, abbreviated title, treatment number, lot number (or numbers, when double-blind), instructions for vaccine administration.

3. Vaccine accountability

The investigator or pharmacist must sign a statement that he/she has received the clinical supplies for the study. At all times the figures on supplied, used and remaining vaccine doses should match. At the end of the study, it must be possible to reconcile delivery records with those of used and unused stocks. An explanation must be given of any discrepancies.

After approval from GSK Biologicals, used vaccine vials/syringes should be destroyed at the study sites using locally approved biosafety procedures and documentation unless otherwise described in the protocol. If no adequate biosafety procedures are available at the study sites, the used vaccine vials/syringes are to be returned to an appropriate GSK

Biologicals site for destruction in accordance with current GSK SOP WWD-1102. Unused vaccine vials/syringes will be disposed at the local GSK Biologicals site in accordance with GSK SOP WWD-1102. If no processes for destruction of unused vaccines are in place in the local GSK Biologicals site; the unused vials/syringes must be returned to GSK Biologicals in Rixensart, Belgium.

4. Transfers of clinical vaccines or products from country medical department or dispatch center to study sites or between sites

Storage temperatures must be maintained during transport and deviations must be reported to Logistics and Packaging for guidance. All transfers of clinical vaccines or products must be documented using the Clinical Supply Transfer Form. If the duration of the transfer is less than four hours, a transportable fridge or any suitable container (e.g. Styrofoam container) with a maximum of eight refrigerated cold packs (cooling elements) must be used in order to maintain the vaccines at 2°to 8°C during transport. If the duration is more than four hours, a transportable fridge or any suitable container (e.g. Styrofoam container) with a minimum of ~~eight~~ ***thirteen*** cold packs (cooling elements) must be used as well as a temperature monitoring system that must be placed as close as possible to the doses and checked upon reception at the final destination. Never place frozen cold packs or dry ice inside vaccine/product boxes for vaccine that must be kept at +4°C in order to avoid cold-chain deviation (e.g. frozen vaccines). Exceptions to these instructions are detailed in product-specific transport guidelines. **Amended (04 July 2006)**

5. Labels for sample identification

The investigator will receive labels from GSK Biologicals to identify samples taken from each subject at each time point. Each label will contain the following information: study number, treatment number, sampling time point (e.g., post vaccination 3), timing (e.g., study Month 7).

6. Other supplies provided by GSK Biologicals

In addition to the vaccines, the study documentation and the sample labels, the investigator will receive the following supplies:

- tubes with screw caps for serum samples,
- racks for the tubes of serum.

Appendix H Amendments and Administrative Changes to the Protocol

GlaxoSmithKline Biologicals<br>Clinical Research & Development<br>**Protocol Amendment Approval**	
**eTrack study number**	106367
**eTrack abbreviated title**	Malaria-047
**IND number**	BB-IND 12937
**Protocol title:**	A Phase II randomized, controlled, partially-blind study of the safety and immunogenicity of GlaxoSmithKline Biologicals' candidate *Plasmodium falciparum* vaccines RTS,S/AS02D and RTS,S/AS01E, when administered IM according to one of three dose schedules in children aged 5 to 17 months living in Ghana.
**Amendment number:**	Amendment 1
**Amendment date:**	04 July 2006
**Co-ordinating author:**	Marie-Sylvie Remacle, Scientific Writer

Rationale/background for changes:

1. **At the Kumasi site (KCCR/SMS), children in the 0, 1, 2-schedule group will receive RTS,S/AS02D as an active comparator, as opposed to Rabies vaccine. Only the children in the Kintampo site (KHRC) will receive the Rabies vaccine as a control.**

The changes are:

- **Synopsis:**

One group of children on the 0, 1, 2-schedule will receive a Rabies vaccine as a control. One group on the same schedule will receive the RTS,S/AS02D experimental vaccine as an active comparator.

~~RTS,S/AS02D will not be assessed on a 0, 1, 2-schedule in this study as it has already been assessed in children during the trials Malaria-025, -026, -034 and is being assessed in Malaria-038 and -040.~~

Experimental design: Phase II, controlled, partially blind (blind to vaccine administration, open to vaccination schedule), randomized (1: 1: 1: 1: 1: 1) trial with six treatment groups ***at each study site***.

In the Kintampo Health Research Center (KHRC), on the 0, 1, 2-schedule, subjects will receive the Rabies vaccine as a control. In the Kumasi Center for Collaborative Research / School of Medical Sciences (KCCR/SMS), on the 0, 1, 2-schedule, subjects will receive the RTS,S/AS02D experimental vaccine as an active comparator. Randomization to each of the ***other*** study groups will be balanced between the two study sites.

- ## Synopsis and Section 2:

For RTS,S/AS01E (on a 0, 1-month and a 0, 1, 2-month and a 0, 1, 7-month schedule) and RTS,S/AS02D (on a 0, 1-month, ***0, 1, 2-month*** and a 0, 1, 7-month schedule) when administered intramuscularly to children aged 5 to 17 months living in a malaria-endemic area.

- ## Synopsis and Section 3:

540 subjects will be enrolled. It is expected that approximately ~~450~~ ***455*** subjects will be evaluable at study end.

- ## Section 1.6:

~~To date, RTS,S/AS02A has been extensively evaluated on a 0, 1, 2-month schedule in children between the ages of 1 and 4 years in Mozambique and found to provide good immunogenicity [GSK data on file, Malaria-025, GSK data on file, Malaria-034] and efficacy [GSK data on file, Malaria-026] with an acceptable safety profile. Because of this extensive prior evaluation, RTS,S/AS02D will not be assessed on a 0, 1, 2-schedule in this trial.~~

- ## Section 3:

Figure 2: Study Design Overview: children to receive RTS,S/AS01E or Rabies vaccine ***or RTS,S/AS02D*** on a 0, 1, 2-month schedule:

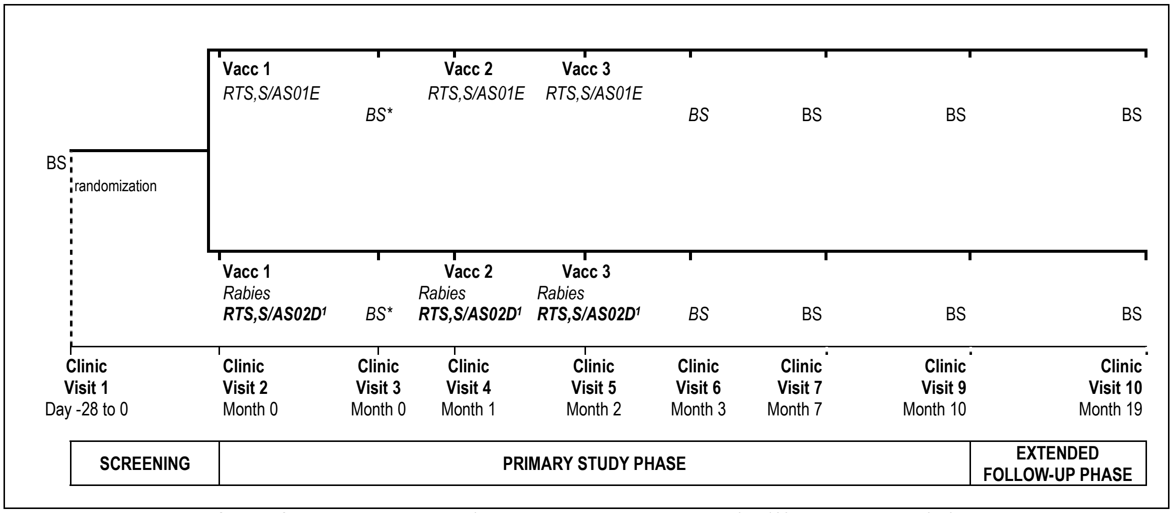

KEY: BS; Blood Sample. **Vacc;** Vaccination. * this blood sample only carried out on first 100 children to present for follow-up

1 The Rabies vaccine will be used only at Kintampo-KHRC; The RTS,S/AS02D vaccine will be used at Kumasi-KCCR/SMS.

Experimental design: Phase II, controlled, partially blind (blind to vaccine administration, open to vaccination schedule), randomized (1: 1: 1: 1: 1: 1) trial with six treatment groups ***at each study site***.

Vaccine	Schedule (Months)	Number of children to be enrolled	Estimated number evaluable
RTS,S/AS01E (0.5 mL dose)	0, 1, 2	90	75
Rabies vaccine (0.5 mL dose)[a]	0, 1, 2	~~90~~ ***45***	~~75~~ ***40***
RTS,S/AS02D (0.5 mL dose)[b]	***0, 1, 2***	***45***	***40***
RTS,S/AS02D (0.5 mL dose)	0, 1, 7	90	75
RTS,S/AS01E (0.5 mL dose)	0, 1, 7	90	75
RTS,S/AS02D (0.5 mL dose)	0, 1	90	75
RTS,S/AS01E (0.5 mL dose)	0, 1	90	75

[a] The Rabies vaccine will only be used at Kintampo – KHRC
[b] Enrollment will occur at Kumasi – KCCR/SMS

- ## Section 5.1.5:

After completion of the study, Rabies immunization on a 0, 1, 28-day schedule will be offered for all children ***enrolled at the KHRC and*** that did not receive it during the study (i.e. those subjects that received RTS,S/AS02D or RTS,S/AS01E).

- ## Section 5.3.2:

Subject numbers will be allocated to all volunteers who are consented for screening by their parent(s)/guardian(s). Subject numbers will be issued consecutively ***at each study site and there will be no overlap of subject numbers between sites. Screening CRFs will therefore be provided by GSK Biologicals to the study sites with prefilled subject numbers***.

- ## Section 5.10:

Table 6: List of study procedures: children to receive RTS,S/AS01E or Rabies vaccine ***or RTS,S/AS02D*** on a 0, 1, 2-month schedule

Table 6, the cell "Administer RTS,S/AS01E or Rabipur Rabies vaccine ***or RTS,S/AS02D***".

Table 6: the footnote "***1 The Rabies vaccine will be used only at Kintampo-KHRC; The RTS,S/AS02D vaccine will be used at Kumasi-KCCR/SMS.***"

Table 9: Intervals between study stages/visits for children to receive RTS,S/AS01E or Rabies vaccine ***or RTS,S/AS02D*** on a 0, 1, 2-month schedule

Table 12: Summary of blood sampling timepoints and volumes to be collected; children to receive RTS,S/AS01E or Rabies vaccine ***or RTS,S/AS02D*** on a 0, 1, 2-month schedule.

- ## Section 5.11.2:

Title of the section: Study visits for children to receive RTS,S/AS01E or Rabies vaccine ***or RTS,S/AS02D*** on a 0, 1, 2-month schedule

Clinic visits 2, 4 and 5: Administer the first dose of RTS,S/AS01E or Rabies Vaccine ***or RTS,S/AS02D*** intramuscularly in the left deltoid.

- ## Section 6.4.1:

In this randomization process, volunteers will be allocated randomly to one of six study groups ***at each study site***, each defining which vaccine should be given ~~(i.e. RTS,S/AS01E, RTS,S/AS02D or Rabies vaccine)~~ and at which schedule (0, 1-month or; 0, 1, 2-month or 0, 1, 7-month). ***For the schedule 0, 1, 2-month at the Kintampo study site (KHRC), this means: RTS,S/AS01E or Rabies vaccine. For the same schedule at the Kumasi study site (KCCR/SMS), this means: RTS,S/AS01E or RTS,S/AS02D. For the other schedules at both study sites, this means either RTS,S/AS01E or RTS,S/AS02D. At each study site,*** all study groups will have the same number of subjects. Each investigator will be supplied with a list that indicates which Treatment Numbers correspond to which vaccination schedules. This procedure will mean that investigators will be blinded to vaccine administered to subjects, but not to the vaccination schedule.

- ## Section 10.5.1:

Safety analyses will be performed on the Total Vaccinated Cohort. A trial of this size has the power to detect only large differences in the frequencies of AEs with reasonable power. A sample size of ***540*** ~~75 evaluable~~ subjects ~~per group~~ has the power to detect differences in the rates of safety endpoints between groups as shown in Table 19:

~~Frequency of events for *x**~~	~~Frequency of events for *y**~~	~~Power to detect difference (number per group equals 90 75)~~
~~1%~~	~~13%~~	~~80%~~
~~3%~~	~~18%~~	~~80%~~
~~5%~~	~~21%~~	~~80%~~
~~10%~~	~~29%~~	~~80%~~

~~* where *x* and *y* are the study groups to be compared~~

**Frequency of events for *x****	**Frequency of events for *y** *N1=N2=90***	**Frequency of events for *y** *N1=90, N2=45***
1%	12%	13%
3%	16%	18%
5%	20%	22%
10%	27%	31%

* where *x* and *y* are the study groups to be compared
80% Power, 2-sided Fisher's exact test, alpha=0.05

- **Appendix G, section 1:**

Sufficient doses for ~~90~~ ***135*** recipients of the candidate vaccine RTS,S/AS02D on a three dose schedule and 90 recipients on a two dose schedule (doses of RTS,S vaccine in monodose vials and doses of AS02D adjuvant in pre-filled syringes).

Sufficient doses for ~~90~~ ***45*** recipients of Rabies vaccine on a three dose schedule.

2. **Since the issue of the first version of the protocol, the IND Study number has been received from the FDA and has been added to the cover pages and in the Investigators' Agreements: BB-IND 12937.**

3. **Other changes have been made to the protocol to correct text, typographical errors or omissions.**

A. Text:

- **Sybopsis:** The information about the Malaria-040 and the Malaria-046 trials have been updated

The Malaria-040 trial, due to start in ~~Q1/Q2~~ ***Q3*** 2006 in Tanzania will assess RTS,S/AS02D when coadministered with DTPw/Hib vaccine.

The first study in which RTS,S/AS01 vaccines ~~will be~~ ***is*** assessed in children is Malaria-046. That study ~~will take~~ ***takes*** place in children in Gabon in children between the ages of 18 months and 4 years in order to investigate safety, reactogenicity and immunogenicity of RTS,S/AS01E.

- **Synopsis and Section 3:** The schedule for vaccination was corrected because vaccination does not start at screening but at study month 0.

PRIMARY STUDY PHASE													EXTENDED FOLLOW-UP PHASE								
Study Month	Scr	0	1	2	3	4	5	6	7	8	9	10	11	12	13	14	15	16	17	18	19
0, 1-month vaccination schedule																					
CS/HBs	**HBs**			**HBs**																	**HBs**
	**CS**			**CS**					**CS**			**CS**									**CS**
CMI				**CMI**																	**CMI**
VACC		**X**	**X**																		
0, 1, 2-month vaccination schedule																					
CS/HBs	**HBs**				**HBs**																**HBs**
	**CS**				**CS**				**CS**			**CS**									**CS**
CMI					**CMI**																**CMI**
VACC	~~**X**~~	**X**	**X**	**X**																	
0, 1, 7-month vaccination schedule																					
CS/HBs	**HBs**									**HBs**											**HBs**
	**CS**								**CS**	**CS**		**CS**									**CS**
CMI										**CMI**											**CMI**
VACC	~~**X**~~	**X**	**X**						**X**												

Scr: Screening VACC: Vaccination

- **Section 6.9:** the timelines for collection of specific medications / treatments have been corrected

Table 16:

**3 months prior to Dose 1 → Dose 1**	All treatments listed as elimination criteria in Section 4.5*
Dose 1 ~~**Screening**~~ **→** ***Study month 10*** ~~**30 Days post Dose 3**~~	All antipyretic, analgesic, antibiotic and any treatments listed as elimination criteria in Section 4.5*<br>All vaccinations
1 Day post Study month 10 ~~**31 Days post Dose 3**~~ **→ Final Study Visit**	All treatments listed as elimination criteria in Section 4.5*

* e.g. any immunoglobulins, other blood products and any immune modifying drugs

- **Section 8.5.1: Definitions for Acceptable limits / normal range for ALT and Grade 1 for Hemoglobin were corrected**

Table 18:

	Acceptable limit/normal range	Toxicity grading scale			
		Grade 1	Grade 2	Grade 3	Grade 4
Hemoglobin	≥ 8.0 g/dL	< ***8.0 g/dL*** ~~ULN~~	< 6.0 g/dL	< 5.0 g/dL	< 5.0 g/dL & clinical signs of heart failure
Total white cell count†	≥ 4.0 x 10^3/µL<br>< 17 x 10^3/µL	2.5 to 4.0 x 10^3 /µL	1.5 to 2.4 x 10^3 /µL	1.0 to 1.4 x 10^3 /µL	< 1.0 x 10^3/µL
Platelets†	≥ 100 x 10^3/µL	50 to 99 x 10^3 /µL	25 to 49 x 10^3/µL	< 25 x 10^3/µL	< 25 x 10^3/µL & clinical signs of bleeding
ALT*	≤ 60 ***IU/L*** ~~µmol/L~~	1.1 to 2.5 x ULN	2.6 to 5.0 x ULN	5.1 to 10.0 x ULN	> 10.0 x ULN
Creatinine*	≤ 60 µmol/L (or 0.6 mg/dL)	1.1 to 1.5 x ULN	1.6 to 3.0 x ULN	3.1 to 6.0 x ULN	> 6.0 ULN or requires dialysis

†: Grading scale adapted from Division of AIDS table for grading severity of adult and pediatric adverse events December 2004
*: Grading scale adapted from WHO Toxicity Grading Scale for Determining Severity of Adverse Events, February 2003.
ULN: Upper Limit of Normal LLN: Lower Limit of Normal

- **Appendix G, section 4: A new SOP has been issued for the number of cold packs for transportation of vaccines**

If the duration is more than four hours, a transportable fridge or any suitable container (e.g. Styrofoam container) with a minimum of ~~eight~~ ***thirteen*** cold packs (cooling elements) must be used as well as a temperature monitoring system that must be placed as close as possible to the doses and checked upon reception at the final destination.

B. Typographical errors:

- **Section 1.6**:

This trial is one of a series of age de-escalation steps with the aim of assessing whether RTS,S/AS01E and RTS,S/AS02~~A~~ ***D*** may be suitable for inclusion in an EPI vaccination program.

- **Section 9.1:**

A subject that comes for Clinic Visit 9 has completed the primary study phase. A subject that comes for Clinic Visit ***10*** ~~11~~ has completed the extended follow-up phase of the study.

- **Section 9.2.1:**

From an analysis perspective, a 'withdrawal' from the study is any subject who was not available for the concluding contact foreseen in the protocol. A subject that comes for Clinic Visit 9 has completed the primary study phase. A subject that comes for Clinic Visit ***10*** ~~11~~ has completed the extended follow-up phase of the study.

C. Omissions:

- ## Investigator's agreements

The sentence "***To ensure that all persons assisting me with the study are adequately informed about the GSK Biologicals investigational product(s) and other study-related duties and functions as described in the protocol.***" has been added in the Investigators' Agreements, to be in accordance with the GSK Biologicals' Protocol Template v12.2.

GlaxoSmithKline Biologicals

Clinical Research & Development

Protocol Amendment Approval

**eTrack study number**	106367
**eTrack abbreviated title**	Malaria-047
**IND number**	BB-IND 12937
**Protocol title:**	A Phase II randomized, controlled, partially-blind study of the safety and immunogenicity of GlaxoSmithKline Biologicals' candidate *Plasmodium falciparum* vaccines RTS,S/AS02D and RTS,S/AS01E, when administered IM according to one of three dose schedules in children aged 5 to 17 months living in Ghana.
**Amendment number:**	Amendment 1
**Amendment date:**	04 July 2006
**Approved by:**<br>Sponsor Signatory title	______________________ ________

GlaxoSmithKline Biologicals

Clinical Research & Development

Protocol Amendment Approval

**eTrack study number**	106367
**eTrack abbreviated title**	Malaria-047
**IND number**	BB-IND 12937
**Protocol title:**	A Phase II randomized, controlled, partially-blind study of the safety and immunogenicity of GlaxoSmithKline Biologicals' candidate *Plasmodium falciparum* vaccines RTS,S/AS02D and RTS,S/AS01E, when administered IM according to one of three dose schedules in children aged 5 to 17 months living in Ghana.
**Amendment number:**	Amendment 1
**Amendment date:**	04 July 2006
**Agreed by:** **Investigator:**	Tsiri Agbenyega
**Investigator signature:**	____________________________________
**Date:**	____________________________________

GlaxoSmithKline Biologicals

Clinical Research & Development

Protocol Amendment Approval

**eTrack study number**	106367
**eTrack abbreviated title**	Malaria-047
**IND number**	BB-IND 12937
**Protocol title:**	A Phase II randomized, controlled, partially-blind study of the safety and immunogenicity of GlaxoSmithKline Biologicals' candidate *Plasmodium falciparum* vaccines RTS,S/AS02D and RTS,S/AS01E, when administered IM according to one of three dose schedules in children aged 5 to 17 months living in Ghana.
**Amendment number:**	Amendment 1
**Amendment date:**	04 July 2006
**Agreed by:**	
**Investigator:**	Jennifer Evans
**Investigator signature:**	______________________________
**Date:**	______________________________

GlaxoSmithKline Biologicals

Clinical Research & Development

Protocol Amendment Approval

**eTrack study number**	106367
**eTrack abbreviated title**	Malaria-047
**IND number**	BB-IND 12937
**Protocol title:**	A Phase II randomized, controlled, partially-blind study of the safety and immunogenicity of GlaxoSmithKline Biologicals' candidate *Plasmodium falciparum* vaccines RTS,S/AS02D and RTS,S/AS01E, when administered IM according to one of three dose schedules in children aged 5 to 17 months living in Ghana.
**Amendment number:**	Amendment 1
**Amendment date:**	04 July 2006
**Agreed by:**	
**Investigator:**	Seth Owusu Agyei
**Investigator signature:**	___________________________
**Date:**	___________________________

GlaxoSmithKline Biologicals

Clinical Research & Development

Protocol Amendment Approval

**eTrack study number**	106367
**eTrack abbreviated title**	Malaria-047
**IND number**	BB-IND 12937
**Protocol title:**	A Phase II randomized, controlled, partially-blind study of the safety and immunogenicity of GlaxoSmithKline Biologicals' candidate *Plasmodium falciparum* vaccines RTS,S/AS02D and RTS,S/AS01E, when administered IM according to one of three dose schedules in children aged 5 to 17 months living in Ghana.
**Amendment number:**	Amendment 1
**Amendment date:**	04 July 2006
**Agreed by:**<br>**Investigator:**<br><br>**Investigator signature:**<br><br><br>**Date:**	 Kwaku Poku Asante<br><br><br>______________________________<br><br>______________________________
